# Light-Mediated Transformation of Renieramycins and Semisynthesis of 4′-Pyridinecarbonyl-Substituted Renieramycin-Type Derivatives as Potential Cytotoxic Agents against Non-Small-Cell Lung Cancer Cells

**DOI:** 10.3390/md21070400

**Published:** 2023-07-13

**Authors:** Suwimon Sinsook, Koonchira Buaban, Iksen Iksen, Korrakod Petsri, Bhurichaya Innets, Chaisak Chansriniyom, Khanit Suwanborirux, Masashi Yokoya, Naoki Saito, Varisa Pongrakhananon, Pithi Chanvorachote, Supakarn Chamni

**Affiliations:** 1 Department of Pharmacognosy and Pharmaceutical Botany, Faculty of Pharmaceutical Sciences, Chulalongkorn University, Bangkok 10330, Thailand; nhamsuwimon1997@gmail.com (S.S.); koonchira.buaban@gmail.com (K.B.); chaisak.c@pharm.chula.ac.th (C.C.); khanit.s@chula.ac.th (K.S.); 2 Pharmaceutical Sciences and Technology Program, Faculty of Pharmaceutical Sciences, Chulalongkorn University, Bangkok 10330, Thailand; 3 Natural Products and Nanoparticles Research Unit (NP2), Chulalongkorn University, Bangkok 10330, Thailand; 4 Department of Pharmacology and Physiology, Faculty of Pharmaceutical Sciences, Chulalongkorn University, Bangkok 10330, Thailand; ikseniksen08@gmail.com (I.I.); korrakod.petsri@gmail.com (K.P.); 6481004120@student.chula.ac.th (B.I.); varisa.p@pharm.chula.ac.th (V.P.); pithi.c@chula.ac.th (P.C.); 5 Center of Excellence in Cancer Cell and Molecular Biology, Faculty of Pharmaceutical Sciences, Chulalongkorn University, Bangkok 10330, Thailand; 6 Graduate School of Pharmaceutical Sciences, Meiji Pharmaceutical University, 2-522-1 Noshio, Tokyo 204-8588, Japan; yokoya@my-pharm.ac.jp (M.Y.); naoki@my-pharm.ac.jp (N.S.); 7 Preclinical Toxicity and Efficacy, Assessment of Medicines and Chemicals Research Unit, Chulalongkorn University, Bangkok 10330, Thailand

**Keywords:** *Xestospongia* sp., cytotoxicity, renieramycins, semisynthesis, light-induced intramolecular photoredox reaction, non-small-cell lung cancer, bis-tetrahydroisoquinoline, molecular docking, molecular dynamics

## Abstract

The semisynthesis of renieramycin-type derivatives was achieved under mild and facile conditions by attaching a 1,3-dioxole-bridged phenolic moiety onto ring A of the renieramycin structure and adding a 4′-pyridinecarbonyl ester substituent at its C-5 or C-22 position. These were accomplished through a light-induced intramolecular photoredox reaction using blue light (4 W) and Steglich esterification, respectively. Renieramycin M (**4**), a bis-tetrahydroisoquinolinequinone compound isolated from the Thai blue sponge (*Xestospongia* sp.), served as the starting material. The cytotoxicity of the 10 natural and semisynthesized renieramycins against non-small-cell lung cancer (NSCLC) cell lines was evaluated. The 5-*O*-(4′-pyridinecarbonyl) renieramycin T (**11**) compound exhibited high cytotoxicity with half-maximal inhibitory concentration (IC_50_) values of 35.27 ± 1.09 and 34.77 ± 2.19 nM against H290 and H460 cells, respectively. Notably, the potency of compound **11** was 2-fold more than that of renieramycin T (**7**) and equal to those of **4** and doxorubicin. Interestingly, the renieramycin-type derivatives with a hydroxyl group at C-5 and C-22 exhibited weak cytotoxicity. In silico molecular docking and dynamics studies confirmed that the mitogen-activated proteins, kinase 1 and 3 (MAPK1 and MAPK3), are suitable targets for **11**. Thus, the structure–cytotoxicity study of renieramycins was extended to facilitate the development of potential anticancer agents for NSCLC cells.

## 1. Introduction

**Figure 1:**
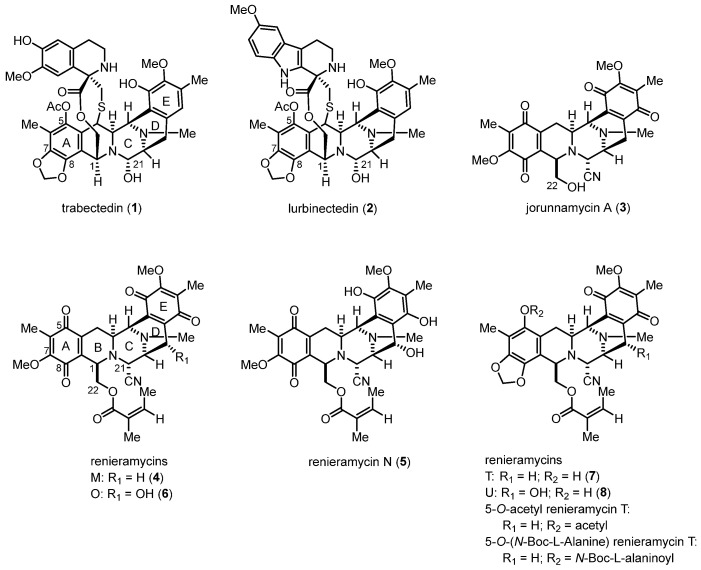
Structures of the bis-tetrahydroisoquinoline alkaloids as anticancer drugs and promising drug leads.

Renieramycins, bis-tetrahydroisoquinolinequinone marine alkaloids, along with ecteinascidins and jorunnamycins, have been reported to exhibit significant anticancer activity (Figure 1) [1,2]. Trabectedin (also known as ecteinascidin 743, **1**), which was isolated from the Caribbean tunicate (*Ecteinascidia turbinate*), was approved by the United States Food and Drug Administration in 2015 for the treatment of advanced soft tissue sarcoma and ovarian carcinoma [3,4,5]. Lurbinectedin (PM01183, **2**), a bis-tetrahydroisoquinoline analog of **1**, was approved for the second-line treatment of metastatic small-cell lung cancer in 2020 [6,7]. Compounds **1** and **2** are chemotherapeutic drugs that covalently bind specifically to the N2 position of guanine in the DNA minor groove, leading to double-strand breaks and apoptosis in cancer cells [3,6].

Renieramycins are isolated from marine sponges, such as genera *Reniera* [8,9], *Cribrochalina* [10], and *Xestospongia* [11,12], and they possess a pentacyclic bis-tetrahydroisoquinolinequinone moiety as the core scaffold (Figure 1). Several renieramycins containing an angelate ester at the C-22 position display potent bioactivities, including antimicrobial [8], antileishmanial [13], and anticancer [11,12,14,15] activities. Interestingly, renieramycins M (**4**) and N (**5**) have been successfully isolated in the gram scale of the Thai blue sponge (*Xestospongia* sp.) [11]. Renieramycins M, N, and O (**4**–**6**) have been demonstrated to exhibit potent cytotoxicity against colon (HCT116) and lung carcinoma (QG56) [11,12]. Additionally, compound **4** has been reported to display strong cytotoxicity against various human cancer cell lines, including colon (DLD1), lung (NCI-H460), pancreatic adenocarcinoma (AsPC1), and ductal breast epithelial (T47D) cells [11,12,14]. Renieramycins T (**7**) and U (**8**), renieramycin–ecteinascidin hybrid marine natural alkaloids containing a 1,3-dioxole ring that is similar to the left-side carbon framework of trabectedin, exhibit strong in vitro anticancer activity against non-small-cell lung cancer (NSCLC) cells [14]. Furthermore, the cytotoxicity mechanisms of the renieramycin–ecteinascidin hybrid derivatives against NSCLC cells have been revealed. Compound **7** was discovered to enhance apoptosis induction via the degradation of the myeloid cell leukemia 1 (Mcl-1) protein in NSCLC [16,17]. Further, it suppressed mouse melanoma (B16F10) cell metastasis and migration through the downregulation of NF-E2-related factor 2 [18]. Moreover, the 5-*O*-ester derivatives of **7**, which possess acetyl and *N*-Boc-L-alaninoyl substituents, induced apoptosis and suppressed cancer stem cell markers via protein kinase B (AKT) inhibition in NSCLC cells [19,20].

**Figure 2:**
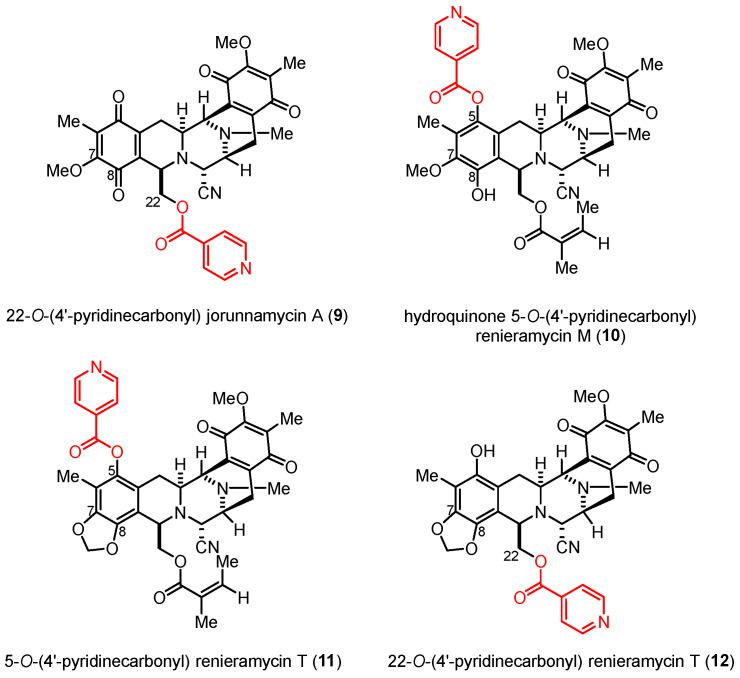
Semisynthetic derivatives of the renieramycin-type derivatives containing a 4′-pyridinecarbonyl ester substituent.

According to the structure–activity relationship (SAR) study, nitrogen-containing heterocyclic ester substituents, particularly 4′-pyridinecarbonyl ester derivatives, as side chains on the renieramycin framework have a remarkable effect on the cytotoxicity of renieramycin against various cancer cells (Figure 2). The 22-*O*-(4′-pyridinecarbonyl) jorunnamycin A compound (**9**) exhibited significantly better cytotoxicity than jorunnamycin A (**3**), its parent compound, against human colon (HCT116), breast (MDA-MB-435), and NSCLC (H292 and H460) cell lines [21,22]. Furthermore, hydroquinone 5-*O*-(4′-pyridinecarbonyl) renieramycin M (**10**) was prepared from **4** by a two-step process, including hydrogenation and Steglich esterification. The resulting derivative (**10**) exhibited cytotoxicity against the highly metastatic H292 and H460 NSCLC cell lines at inhibitory concentration (IC_50_) values in the nanomolar range [15].

Regarding the continuous structure–cytotoxicity relationship study of the renieramycin-type derivatives, the semisynthesis of the renieramycin–ecteinascidin hybrid derivatives of natural bis-tetrahydroisoquinolinequinones (**4**–**6**) was conducted via a facile, light-induced intramolecular photoredox reaction to transform quinone ring A into the 1,3-dioxole-bridged phenolic moiety. The mild photoredox reaction enables the selective one-step modification of the methoxy-substituted quinone unit located on ring A of renieramycins, resulting in the formation of 5-hydroxy-tetrahydroisoquinol-1,3-dioxoles with excellent yields [23]. This transformation yields a series of renieramycin–ecteinascidin derivatives that closely resemble the structure of ring A found in Trabectedin and Lurbinectedin, well-known tetrahydroisoquinoline-based chemotherapeutic drugs. Next, a series of 4′-pyridinecarbonyl esters for the renieramycin-type derivatives were prepared by mild and selective Steglich esterification. The new 4′-pyridinecarbonyl esters, **11** and **12**, were obtained (Figure 2). The in vitro cytotoxicity of the compounds against human H292 and H460 NSCLC cell lines was evaluated using the 3-(4,5-dimethylthiazol-2-yl)-2,5-diphenyltetrazolium bromide (MTT) assay. Additionally, the in silico prediction of the target genes and molecular pathways associated with the activity of the compounds was achieved by virtual network pharmacology study, including molecular docking and molecular dynamics studies.

## 2. Results and Discussion

### 2.1. Light-Induced Intramolecular Photoredox Reaction of Renieramycins

**Scheme 1:**
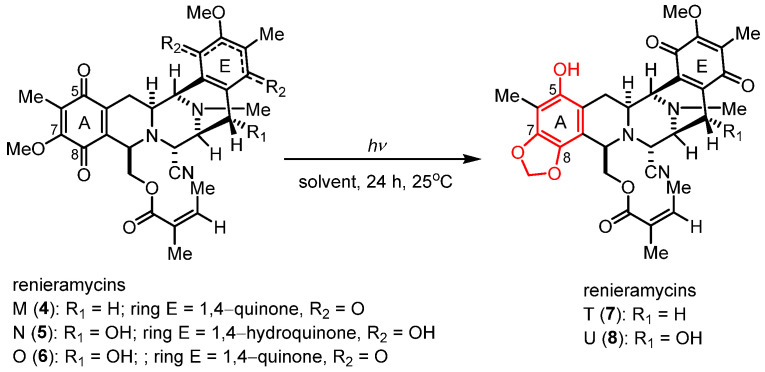
Photoredox reaction of the natural renieramycins.

Renieramycins **4**–**6** were isolated from the Thai blue sponge (*Xestospongia* sp.) by pretreatment with 10% potassium cyanide following a previously reported protocol [11,12]. The photoredox reactions occurring through the light-induced radical formation and intramolecular cyclization of the natural bis-tetrahydroisoquinolinequinone alkaloids (**4**–**6**) were investigated (Scheme 1 and Table 1) [23]. To determine the optimal reaction conditions, we experimented with compound **4**, along with various solvents and light sources. Through irradiation by an 18-W fluorescent lamp (white light) in dichloromethane (CH_2_Cl_2_) for 24 h, compound **4** was smoothly transformed into compound **7** in 64% yield (Table 1, entry 1). The application of chloroform (CHCl_3_) and tetrahydrofuran (THF) as solvents for the photoredox reaction of compound **4** furnished compound **7** in 46% and 47% yields, respectively (Table 1, entries 2 and 3). Further improvement in the yield was achieved when the reaction solution was exposed to irradiation by a 4-W light-emitting diode (LED) lamp (blue light) in CH_2_Cl_2_, affording **7** in excellent yield (81% yield, entry 4). However, using CHCl_3_ and THF with blue light irradiation decreased the yields of **7** to 51% and 54%, respectively (Table 1, entries 5 and 6). Thus, optimized photoredox reactions using blue light and CH_2_Cl_2_ were further performed with natural alkaloids **5** and **6** (Table 1, entries 7 and 8).

**Scheme 2:**
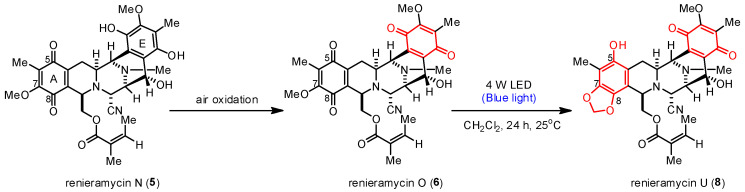
Proposed transformation mechanism of **5** into **6** and **8**.

Unexpectedly, the photoredox reaction of compound **5** yielded a mixture of bis-tetrahydroisoquinolinequinones **6** and **8** (Table 1, entry 7). Compound **8** was obtained as the major product in 37% yield, two times higher than the yield of compound **6** (18% yield). Meanwhile, the photoredox reaction of compound **6** under the optimized condition yielded compound **8** in 48% yield (Table 1, entry 8). Therefore, the light-induced radical formation and intramolecular cyclization of compound **5** to yield compounds **6** and **8** were proposed to occur via stepwise transformations. The mechanism involved air oxidation to convert the unstable 1,4-hydroquinone into the 1,4-quinone moiety, followed by the photoredox reaction [24] (Scheme 2). The results of the light-mediated transformation highlight the chemical diversity of the natural renieramycins found around the blue *Xestospongia* sponge habitat.

### 2.2. Semisynthesis of 4′-Pyridinecarbonyl-Substituted Renieramycin-Type Derivatives

**Scheme 3:**
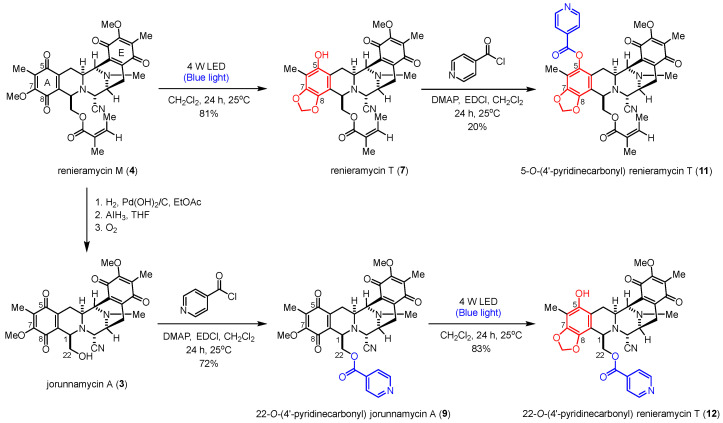
Semisynthesis of the 4′-pyridinecarbonyl-substituted renieramycin-type derivatives.

The semisynthesis of the 4′-pyridinecarbonyl-substituted renieramycin–ecteinascidin hybrid derivative (**11**) started with the photoredox transformation and esterification (Scheme 3). The irradiation of **4** under the 4-W LED light in CH_2_Cl_2_ furnished **7**. Subsequently, a 4′-pyridinecarbonyl motif of **7** was installed by Steglich esterification using isonicotinoyl chloride as an acylating agent, 1-ethyl-3-(3-dimethylaminopropyl) carbodiimide (EDCI) as a coupling reagent, and 4-dimethylaminopyridine (DMAP) as a nucleophilic base catalyst to obtain the desired product (**11**) in acceptable yield. The semisynthesis protocol of the renieramycin-type derivative (**12**) was envisioned. Compound **3** was prepared from compound **4** by hydrogenation, hydride reduction, and air oxidation [21,24]. Ester compound **12** was obtained in excellent yield by attaching the 4′-pyridinecarbonyl substituent to the C-22 position of renieramycin by Steglich esterification, followed by the formation of the 1,3-dioxole-bridged phenolic moiety via light-induced radical formation and intramolecular cyclization.

The chemical structures of all semisynthetic renieramycin-type derivatives were characterized by spectroscopic techniques (see Supporting Information for the spectra, Figures S1–S15). The spectral data of compounds **7**–**9** were consistent with previous reports [14,22]. Regarding the 4′-pyridinecarbonyl-substituted renieramycin–ecteinascidin hybrid derivatives (**11** and **12**), the characteristic chemical shift of the methylene moiety (–CH_2_–) at the newly constructed 1,3-dioxole ring showed a proton signal as a pair of doublets at 5.95 ± 0.07 ppm and a carbon signal at 101.6 ± 0.3 ppm. Moreover, the C-7 and C-8 quaternary carbon signals of both compounds **11** and **12** shifted upfield, compared with the signals of the parent compound **4**. The carbonyl carbon signals of the resulting 4′-pyridinecarbonyl ester were confirmed at 167.0 and 164.2 ppm for **11** and **12**, respectively. Moreover, the chemical shifts of the carbonyl carbon at C-15 and C-18 for both **11** and **12** are located at 186.0 ± 0.2 ppm and 182.5 ± 0.1 ppm, respectively, indicating the presence of the quinone group on ring E.

**Figure 3:**
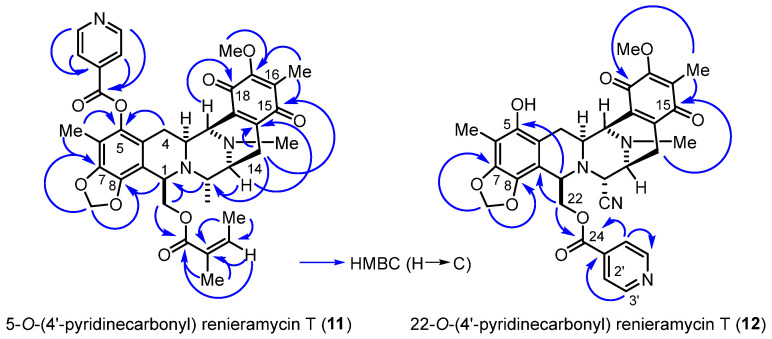
HMBCs (blue arrows) for the 4′-pyridinecarbonyl-substituted derivatives (**11** and **12**).

The heteronuclear multiple bond correlations (HMBCs) between the methylene proton at the 1,3-dioxole ring and the quaternary aromatic carbons at C-7 and C-8 clearly confirmed the fused ring structure at ring A in compounds **11** and **12** (Figure 3). The key HMBCs of compound **11** included the correlations between the C-5 quaternary carbon and both the C-4 methylene protons and the C-6 methyl protons, along with those of the C-1′ pyridine carbon, all the protons at the C-2′ and C-3′ position of pyridine, and C-6 methyl motifs. Compound **12** exhibited significant HMBCs between the C-24 carbonyl carbon and both the C-22 methylene protons and the C-2′ pyridine proton.

### 2.3. Cytotoxicity of Compounds ***3**–**12*** against NSCLC Cell Lines

The in vitro cytotoxic activities of the series of semisynthesized renieramycin-type derivatives and the natural renieramycins analogs were analyzed by MTT assay against the human H292 and H460 NSCLC cell lines (Table 2). The positive controls were cisplatin and doxorubicin, which were the first-line chemotherapeutic drugs. The results showed that compound **3**, which has a primary alcohol at the C-22 position, and renieramycins **5**–**8**, which possess unique structures (1,4-quinone or 1,3-dioxole-bridged phenol at ring A, methylene or secondary alcohol at C-14 of ring D, and 1,4-quinone or 1,4-hydroquinone at ring E), exhibited weak cytotoxicity against both H292 and H460 NSCLC cell lines at the IC_50_ values of 72.64–183.37 nM (Table 2, entries 1 and 3–6). However, compound **4** exhibited the strongest cytotoxicity among the series of natural renieramycins at the IC_50_ values of 35.36 and 33.86 nM against H292 and H460 cells, respectively (Table 2, entry 2). The semisynthesized renieramycin-type derivatives (**9**–**11**) exhibited substantially high cytotoxic activity at nanomolar concentrations against both cell lines (Table 2, entries 7–9). The known semisynthesized alkaloid (**9**), which possesses bis-tetrahydroisoquinolinequinone and 4′-pyridinecarbonyl moieties, exhibited the strongest cytotoxicity among all the renieramycin derivatives in this study. The compound **9** was approximately 10-fold and 9-fold more potent than the parent compound (**4**) against H292 and H460 NSCLC cell lines, respectively [22]. Both compound **10**, the known 8-hydroquinone, and compound **11**, the renieramycin–ecteinascidin hybrid derivative, contained the 5-*O*-(4′-pyridinecarbonyl) ester. They exhibited significant cytotoxicity, similar to those of **4** and docxorubicin, at the same IC_50_ level. Moreover, compound **11** was 3-fold and 2-fold more potent than **7**, the parent compound, against the H292 and H460 cell lines. Interestingly, compound **12**, a renieramycin-type derivative with a hydroxyl group at C-5, lost its cytotoxic activity and inhibited both the H292 and H460 cell lines at micromolar IC_50_ values (Table 2, entry 10). However, compound **12** exhibited 3-fold and 2-fold stronger potency than cisplatin against the H292 and H460 NSCLC cell lines, respectively. Therefore, the presence of the 4′-pyridinecarbonyl ester and the chemical structure of ring A constitute the essential pharmacophore controlling the cytotoxic potency [15,22,25]. The cytotoxicity of the renieramycin-type derivatives having a hydroxyl group at either the C-5 or C-22 position was weak.

### 2.4. In Silico Prediction of the Target Genes and Molecular Pathways

The series of the 4′-pyridinecarbonyl ester of the renieramycin-type derivatives (**10** and **11**) exhibited significant cytotoxicity against NSCLC cells. The anticancer mechanisms of compounds **10** and **11** were not investigated because of their limited quantity. Regarding data mining from several databases, pharmaco-mapping, and computational strategies, network pharmacology has recently emerged as a comprehensive tool for elucidating the putative biomolecular targets, signaling pathway complexities, and protein–protein interactions (PPIs) of potential leads [26,27]. Therefore, the inhibition mechanism and molecular pathways of both **10** and **11** against NSCLC were investigated by molecular docking and molecular dynamics studies, enabling the identification of the primary target genes.

#### 2.4.1. Investigation of the Potential Targets against NSCLC

**Figure 4:**
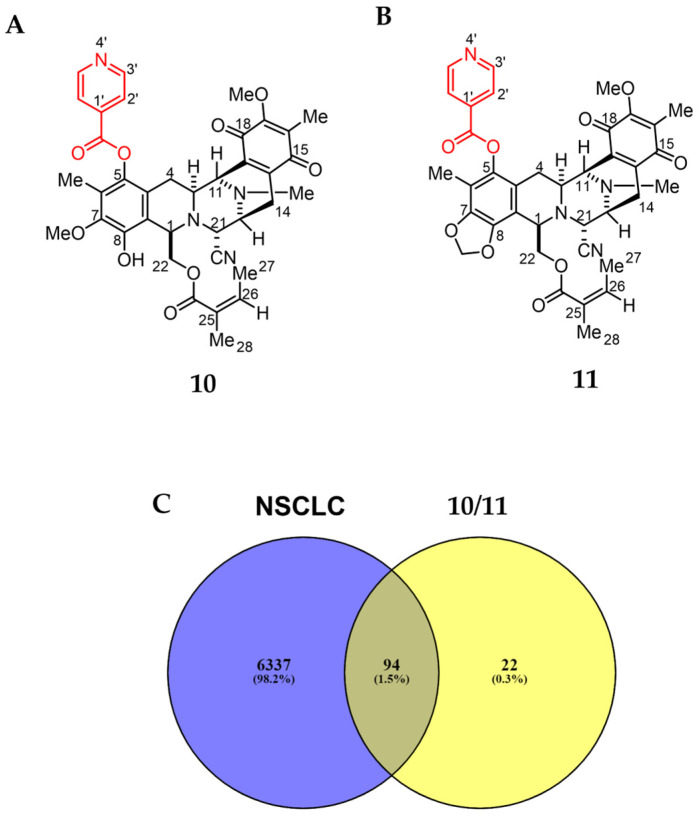
Structure and putative analysis of **10** and **11** against NSCLC. (**A**) Structure of **10**. (**B**) Structure of **11**. (**C**) Venn diagram analysis showing both **10** and **11** associated with 116 targets, whereas the NSCLC cells are associated with 6431 targets. The interception analysis showing 94 overlapping targets between **10**/**11** and NSCLC.

The Swiss Target Prediction online tool was employed to compare the renieramycin-type derivatives (**10** and **11**) to a massive internal pharmacophore model database to identify prospective drug targets using reverse pharmacophore alignment (Figure 4) [28]. One hundred and sixteen potential targets related to **10** and **11** were found. From the following four databases: GeneCards, Therapeutic Targets Database (TTD), Online Mendelian Inheritance in Man (OMIM), and DisGeNET, 6431 genes were found in association with NSCLC. Overlapping the corresponding genes using a Venn diagram, 94 prospective targets of **10** and **11** against NSCLC were generated (Figure 4C).

#### 2.4.2. Protein–Protein Interaction Network Construction and Core Target Identification

**Figure 5:**
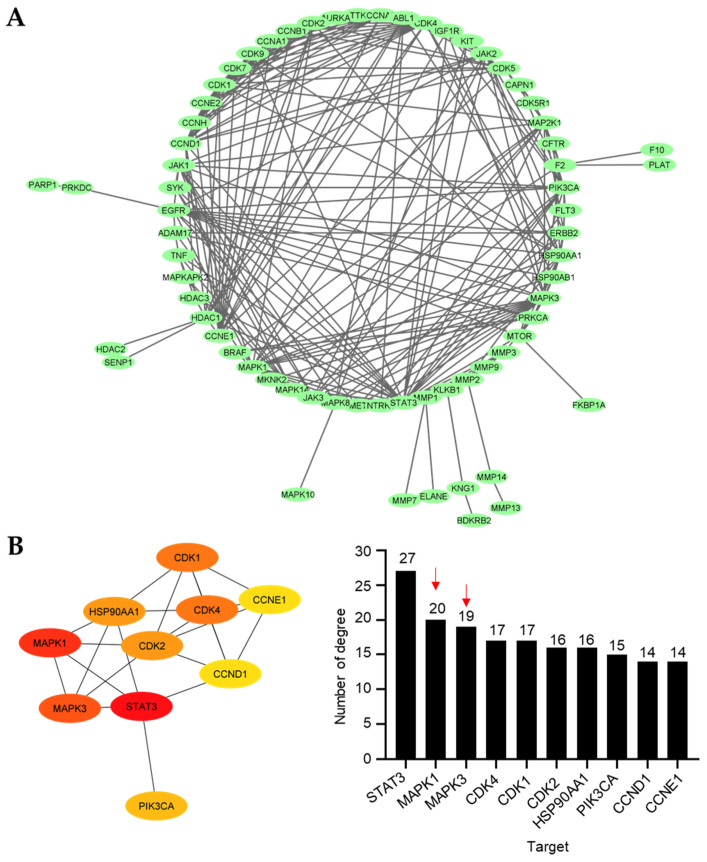
Protein–protein interaction (PPI). (**A**) The main cluster of the PPI obtained from the 94 overlapping targets and visualized using Cytoscape 3.9.1. (**B**) Top 10 targets based on the number of degrees visualized by the CytoHubba plugin. More than 10 degrees are displayed for all the main targets. The intensity of the colors represents the degree values, where red, orange, and yellow correspond to large, medium, and small values, respectively. Red arrow indicates the MAPK family.

To envision the relationship between the 94 prospective targets matched with **10** and **11** against NSCLC, a PPI network was retrieved and visualized using Cytoscape v3.9.1 after the resulting putative targets were loaded into the STRING platform (Figure 5A). The PPI network illustrates the interactions between the targets, with highly linked proteins in the network having a comparatively high number of interactions. CytoHubba, a plugin for Cytoscape, was employed to perform a topological analysis, which revealed the top 10 proteins linked to the number of degrees (Figure 5B). The colors from yellow to red represent small to large degrees. The results revealed several proteins, including signal transducer and activator of transcription 3 (STAT3), mitogen-activated protein kinase 1 (MAPK1), mitogen-activated protein kinase 3 (MAPK3), cyclin-dependent kinase 4 (CDK4), cyclin-dependent kinase 1 (CDK1), cyclin-dependent kinase 2 (CDK2), 90 kDa heat shock protein (HSP90AA1), phosphatidylinositol-4,5-bisphosphate 3-kinase catalytic subunit alpha (PIK3CA), cyclin D1 (CCND1), and cyclin E1 (CCNE1), as the 10 core hub proteins serving as potential therapeutic targets against NSCLC. Regarding the previous virtual study, gene ontology and KEGG pathway enrichment analyses depicted that MAPKs were potential targets of **9** toward the induction of NSCLC apoptosis [29]. Thus, we focused on MAPK1 and MAPK3 for further analysis due to the structural similarities between **9** and **11**, which are the 4′-pyridinecarbonyl esters of the tetrahydroisoquinolinequinone alkaloids.

#### 2.4.3. Molecular Docking of MAPK1 and MAPK3

**Figure 6:**
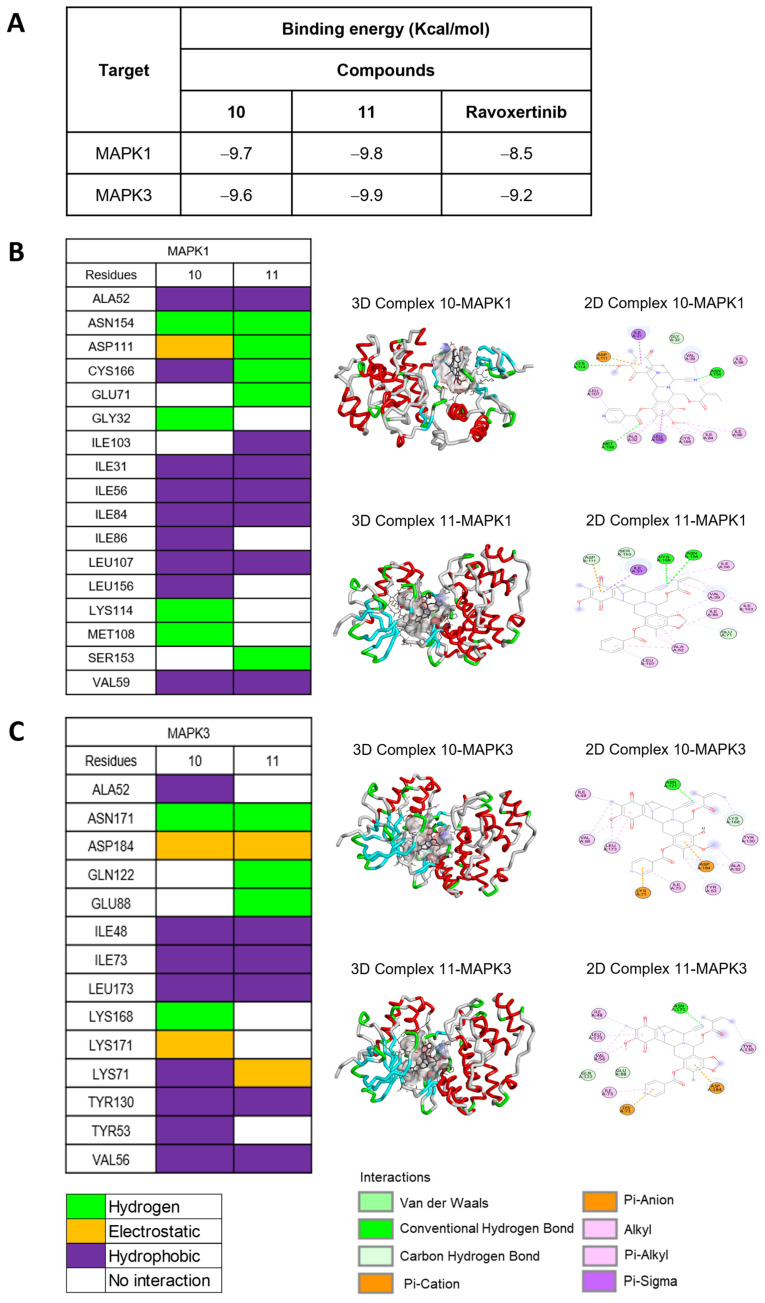
Molecular docking of **10** and **11** with both MAPK1 (ERK2) and MAPK3 (ERK1). (**A**) Binding energies indicate that the interactions of **10** and **11** with both MAPK1 and MAPK3, compared to Ravoxertinib, a known inhibitor. (**B**) Interactions between the amino acid residues of MAPK1 and both **10** and **11**. (**C**) Interactions between the amino acid residues of MAPK3 and both **10** and **11**.

To explore the binding relationships between the MAPK1 and MAPK3 proteins, compounds **10** and **11** were docked in comparison with Ravoxertinib, a known inhibitor of both MAPK1 and MAPK3 (see Supporting Information, Figure S16). From the binding energy calculations, **11** exhibited the lowest binding energies of −9.8 kcal/mol and −9.9 kcal/mol, respectively, for MAPK1 and MAPK3, which was better than those of **10** and Ravoxertinib (Figure 6A). Furthermore, docking studies were conducted for the protonated forms of compounds **10** and **11**, specifically protonated at the nitrogen at position 12, as shown in the Supporting Information (Figure S17). Both the neutral and protonated forms of compounds **10** and **11** exhibited similar binding energy values. Docking with MAPK-1 revealed that both the neutral and protonated forms of compounds **10** and **11** predominantly displayed comparable binding interactions with amino acid residues. Interestingly, the protonated forms of compounds **10** and **11** exhibited hydrogen bonding interactions rather than hydrophobic interactions. Docking with MAPK-3 demonstrated that the protonated forms of compounds **10** and **11** could engage in additional interactions at the enzyme site. However, these additional interactions primarily resulted from hydrophobic interactions, which are generally weaker compared to hydrogen bonding interactions. Upon considering the binding interactions of amino acid residues at the enzyme active site, the neutral forms of compounds **10** and **11** exhibited a closer match with the known ligand, Ravoxertinib, compared to the protonated forms. Specifically, for MAPK-1, the neutral forms of compounds **10** and **11** demonstrated interactions with amino acid residues, including ASP111, ILE31, ILE103, and LEU156. For MAPK-3, the neutral forms of compounds 10 and 11 displayed interactions with LEU173 and VAL56. The number of interacting bonds involving hydrogen, hydrophobic interactions, and electrostatic interactions demonstrated the stability of both **10** and **11** in the binding pocket of both MAPK1 and MAPK3. However, compound **11** interacted with both MAPK1 and MAPK3 with stronger hydrogen binding than that of **10** (Figure 6B,C). Regarding the amino acid residues of MAPK1 that interacted with **11**, the key binding site of MAPK1 comprised ALA52, ASN154, ASP111, CYS166, GLU71, and ILE103. The interactions between **11** and MAPK3 at the specific amino acid residues involved ASN171, GLN122, ILE48, LEU173, LYS71, and TYR130. Interestingly, compounds **10** and **11**, along with compound **9**, had similar binding sites, which confirmed that MAPK1 and MAPK3 are the biomolecular targets. The docking conformations of **11** with MAPK1 and MAPK3 demonstrated stable ligand–receptor binding and increased interaction, thereby affirming the necessity of validating the interaction stability through molecular dynamics simulation.

#### 2.4.4. Molecular Dynamics Analysis of MAPK1 and MAPK3 with **11**

**Figure 7:**
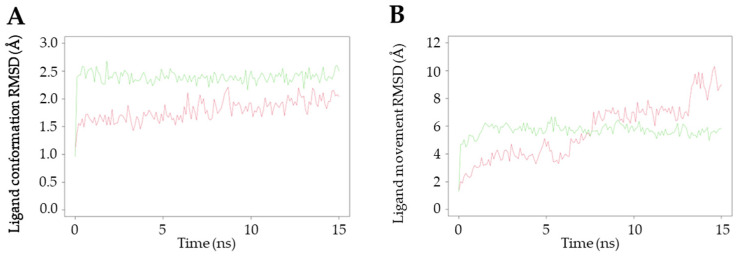
Molecular dynamics study of the interactions between **11** and MAPK1 and MAPK3. Green line shows the interaction with MAPK1, whereas the red line shows the interaction with MAPK3. (**A**) The ligand conformation and (**B**) ligand movement interaction of **11** with MAPK1 and MAPK3.

To gain an in-depth understanding of the protein–ligand interaction of **11**, a molecular dynamics study was conducted on the core proteins of MAPK1 and MAPK3, utilizing the protein–ligand complexes obtained from the results of the molecular docking simulations. When the trajectory exhibits steep changes, it indicates that the system has undergone a significant transition, whereas a smooth trajectory signifies that the system has reached equilibrium. One crucial parameter in molecular dynamics simulations is the root mean square deviation (RMSD), which serves as a measure of the stability of the trajectory. In this simulation, the conformation and movement of the ligand were analyzed using the RMSD value. The RMSD (Å) values of the ligand conformations for MAPK1 and MAPK3 were found to be 2.38 ± 0.15 and 1.79 ± 0.19, respectively, as depicted in Figure 7A. The ligand conformation analysis revealed that both MAPK1 and MAPK3 maintained consistent proximity throughout the entire 15 ns duration of the experiment. However, when comparing the RMSD values, the interaction between **11** and MAPK3 exhibited greater stability in the ligand conformation, and both protein targets did not show significant differences in terms of their interactions. Additionally, the RMSD (Å) of the ligand movement between **11** and these targets was assessed. The results indicated that the average RMSD values for MAPK1 and MAPK3 were 5.68 ± 0.52 and 5.63 ± 2.04, respectively (Figure 7B). A relatively small RMSD of the ligand movement indicates good proximity between the ligand and the targets. The stability of the interaction between compound **11** and MAPK3 showed a greater tendency to fluctuate at 5, 7.5, and 13.2 ns compared to the interaction with MAPK1, particularly at 5 ns intervals. 

Considering all these findings, we hypothesized that **11** interacts with multiple targets in the MAPK family. Among these targets, MAPK1 demonstrated the highest potential and consistently engaged in strong interactions with **11**, aligning with our previous investigation. According to previous studies investigating the structure-cytotoxicity relationship, which employed both in vitro and in silico approaches, the mechanistic pathways underlying the anti-lung cancer effects of marine alkaloids from the tetrahydroisoquinolinequinone family have been elucidated. Specifically, compounds such as 5-*O*-(*N*-Boc-L-alanine)-renieramycin T, a renieramycin-ecteinascidin derivative [20], (1R,4R,5S)-10-(benzyloxy)-9-methoxy-8,11-dimethyl-3-(thiazol-5-ylmethyl)-1,2,3,4,5,6-hexahydro-1,5-epimi- nobenzo [d]azocine-4-carbonitrile (DH_25), a right-half C–E ring analog of renieramycins [30], and 22-*O*-(4′-pyridinecarbonyl) jorunnamycin A, a 4′-pyridinecarbonyl substituted renieramycin-type derivative [29], have demonstrated efficacy against non-small cell lung cancer (NSCLC) cells through modulation of the protein kinase B (Akt), myeloid cell leukemia-1 (MCL-1), and mitogen-activated protein kinase (MAPK) signaling pathways, leading to the induction of apoptosis. The proteins Akt, MCL-1, and MAPK are interconnected targets of cellular signaling networks involved in the regulation of apoptosis. While the MAPK pathway exerts pro-apoptotic effects, Akt signaling generally promotes cell survival. Furthermore, MCL-1, functioning as an anti-apoptotic protein, is influenced by both the MAPK and Akt pathways, thereby contributing to the delicate balance between cell survival and apoptosis in cancer cells [31,32]. Therefore, targeting these signaling pathways and their interactions holds promise for therapeutic interventions aimed at inducing apoptosis in cancer therapy.

## 3. Materials and Methods

### 3.1. General Experimental Procedures

All commercial reagents were purchased from Tokyo Chemical Industry, Tokyo, Japan. The glassware was dried in an oven (105 °C) before the experiment. Photochemical reactions were carried out using a commercially supplied LED (4W LED Filament GLS Blue, 220–240 V, EVE) and 18-W fluorescent light (100 V, HATAYA, Tokyo, Japan). All reactions were monitored by thin-layer chromatography (TLC) using aluminum silica gel 60 F254 (Merck, Darmstadt, Germany), and the TLC chromatogram was recorded under UV light at 254 and 365 nm. Flash column chromatographic purification was performed with silica gel (230–400 mesh) using a mixture of ethyl acetate and hexane as the mobile phase. Moreover, ^1^H and ^13^C NMR spectra were recorded on a Bruker ADVANCE NEO NMR spectrometer (Bruker, Massachusetts, MA, USA) at 400 and 100 MHz, respectively. Deuterated chloroform (CDCl_3_) was used as the internal standard for the ^13^C (77.0 ppm) and ^1^H (7.27 ppm) spectroscopic analyses. Infrared spectra were measured using a Perkin Frontier Fourier-Transform Infrared Spectrometer (PerkinElmer, Massachusetts, MA, USA). High-resolution mass spectrometry was conducted using a microTOF Bruker Daltonics mass spectrometer. Optical rotations were measured with a JASCO P-2000 polarimeter using a 1-mL cell with a cell path length of 1 dm. The spectra of electronic circular dichroism were obtained using a JASCO J-815 CD spectrometer (JASCO, Maryland, MD, USA).

### 3.2. Extraction and Isolation

With assistance from the Aquatic Resources Research Institute, Chulalongkorn University and approval from the Department of Fisheries, Ministry of Agriculture and Cooperatives, Thailand (0510.2/8234, Date: 28 October 2019), a Thai blue sponge (*Xestospongia* sp.) was collected during a SCUBA diving mission at a depth of 3–5 m at Sichang Island, the Gulf of Thailand. The fresh blue sponge was mashed and subsequently pre-treated with 10% potassium cyanide solution in a phosphate-buffered solution at pH 7. Next, the suspension was macerated with methanol. The extraction process was repeated three times. The methanolic extracts were filtered, combined, and concentrated at reduced pressure. The condensed aqueous-methanolic extract was partitioned with hexane and ethyl acetate. The ethyl acetate extract was further concentrated and purified by silica gel column chromatography using hexane and ethyl acetate as the eluent. Natural renieramycins, including renieramycin M (**4**), renieramycin N (**5**) [11], and renieramycin O (**6**) [12], were obtained. Thereafter, compound **4** was transformed into Jorunnamycin A (**3**) under a three-step semisynthesis including hydrogenation, hydride reduction, and air oxidation [21,24].

### 3.3. Screening of the Photoredox Reaction Conditions with the Renieramycins

Natural renieramycins **4**–**6** were employed as the starting material for the light-induced intramolecular photoredox reaction. A solution of the renieramycin alkaloid (15 mg) with 20 mL of the selected solvent (CH_2_Cl_2_, CHCl_3_, and THF) was stirred vigorously under a light source comprising white light (18 W fluorescent) and blue light (4 W LED) for 24 h at room temperature under a nitrogen atmosphere. The reaction was monitored by TLC using a solution of ethyl acetate and hexane (1:1 *v*/*v*) as the mobile phase. After the completion of the reaction, the solvent was evaporated under reduced pressure. The resultant crude product was purified by flash column chromatography using silica gel as the stationary phase and the mixed solvents of ethyl acetate and hexane as the mobile phase to afford the corresponding products, **7** and **8**.

### 3.4. Procedure for the Semisynthesis of Compound ***11***

Compound **4** (15 mg, 0.03 mmol) in dry CH_2_Cl_2_ (20 mL) was irradiated by a 4 W LED lamp (blue light) at room temperature under a nitrogen atmosphere for 24 h. The reaction was monitored by TLC using a hexane and ethyl acetate solution (1:1 *v*/*v*) as the mobile phase. After the completion of the reaction, the solvent was removed under reduced pressure. The crude product was precipitated using hexane, affording **7** as a yellow amorphous solid. Compound **7** was employed in the next step without further purification. Next, compound **7** (21 mg, 0.04 mmol), 4-dimethylaminopyridine (DMAP) (5 mg, 0.04 mmol), 1-(3-dimethylaminopropyl)-3-ethylcarbodiimide hydrochloride (EDCI) (8 mg, 0.04 mmol), and isonicotinoyl chloride hydrochloride (32 mg, 0.18 mmol) were dissolved in CH_2_Cl_2_ (20 mL). The mixture was stirred for 24 h at room temperature under a nitrogen atmosphere. After the completion of the reaction, as determined by TLC, the reaction mixture was quenched by adding distilled H_2_O (20 mL) and extracted with CH_2_Cl_2_ (20 mL, three times). The organic layers were combined and dried over anhydrous magnesium sulfate (MgSO_4_), filtered, and concentrated under reduced pressure. After purification by silica gel flash chromatography using the hexane and ethyl acetate solution as the eluent, 5-*O*-(4′-pyridinecarbonyl) renieramycin T (**11**) was obtained in 20% yield as a yellow amorphous powder.

### 3.5. Procedure for the Semisynthesis of Compound ***12***

Jorunnamycin A (**3**) (25 mg, 0.05 mmol) was placed into an oven-dried, round-bottomed flask and dissolved in dry CH_2_Cl_2_ (10 mL). Thereafter, DMAP (31 mg, 0.25 mmol), EDCI (49 mg, 0.25 mmol), and isonicotinoyl chloride hydrochloride (45 mg, 0.25 mmol) were added. The mixture was stirred for 24 h at room temperature under a nitrogen atmosphere and monitored by TLC using a mixture solvent of ethyl acetate and hexane (1:1 *v*/*v*) as the mobile phase. After completion, the solution was concentrated under reduced pressure. Next, the resultant crude product was purified by flash column chromatography using silica gel as the stationary phase and the mixed solvent of ethyl acetate and hexane as the mobile phase to yield a yellow product (**9**) in 72% yield. Subsequently, compound **9** (6 mg, 0.01 mmol) was dissolved in CH_2_Cl_2_ (15 mL) and irradiated by a 4 W LED lamp (blue light) at room temperature under a nitrogen atmosphere for 24 h. The reaction mixture was monitored by TLC and further purified following the procedure described above to obtain 22-*O*-(4′-pyridinecarbonyl) renieramycin T (**12**) as an amorphous yellow powder in 83% yield.

### 3.6. Cytotoxic Evaluations against NSCLC Cell Lines

The in vitro cytotoxicity of compounds **3**–**12** against H292 and H460 NSCLC cell lines was determined by MTT colorimetric assay. The H292 and H460 cultured cell lines were obtained from the American Type Culture Collection (Manassas, VA, USA). The Roswell Park Memorial Institute (RPMI) medium 1640, fetal bovine serum (FBS), L-glutamine, a penicillin/streptomycin solution, and Albumax I were purchased from Gibco (Gaithersburg, MA, USA). Dimethyl sulfoxide (DMSO) was acquired from Merck Millipore (Billerica, MA, USA) or Sigma-Aldrich. All cell lines were cultured in the RPMI 1640 medium, supplemented with 10% FBS, 2 mM L-glutamine, and 100 units/mL of penicillin/streptomycin, and the temperature was maintained at 37 °C under 5% CO_2_. Cells were trypsinized and seeded at a density of 5000 cells/well with the RPMI media for 24 h in a 96-well plate. A 10 mm stock solution of each test compound in DMSO was prepared and diluted into the serial concentrations at 1–250 nM, with a DMSO concentration of less than 0.2% *v*/*v*. The cells were treated with various concentrations of each derivative for 72 h. Next, the cells were incubated with 0.5 mg/mL of MTT for 2 h. After solubilizing the resulting formazan salt with 100 µL of DMSO, the absorbance of the formed formazan was measured by a spectrophotometric microtiter plate reader (Perkin Elmer Victor 3 1420 Multilabel Plate Counter, Massachusetts, MA, USA) at 570 nm. The experiment was performed in three replicate wells with at least five concentrations of the tested compounds. The cell viability was determined using the GraphPad Prism software (version 5), calculated as a percentage of non-treated control cells and the mean of the half-maximal inhibitory concentration (IC_50_) values. Cisplatin and doxorubicin were used as the positive controls, and 0.2% DMSO was used as the negative control.

### 3.7. Network Pharmacology Study

#### 3.7.1. Database Mining and Identification of **10**, **11**, and NSCLC-Related Targets

The Swiss Target Prediction database (http://www.swisstargetprediction.ch), which was accessed on 14 January 2023, was utilized to gather information on the probable targets of **10** and **11**. To identify potential targets associated with NSCLC cells, various human genetic databases, such as GeneCards, TTD, OMIM, and DisGeNET, were screened. A Venn diagram illustrating the overlapping targets between **10** and **11** with respect to NSCLC can be viewed at this link (https://bioinfogp.cnb.csic.es/tools/venny/, accessed on 15 March 2023). The Venn diagram was generated using Venny 2.1.0 (Centro Nacional de Biotecnología, Madrid, Spain), https://bioinfogp.cnb.csic.es/tools/venny/, accessed on 15 March 2020.

#### 3.7.2. Construction of Protein–Protein Interaction Network and Identification of Potential Core Targets

The overlapping targets derived from the Venn diagram were inputted into the STRING database. For STRING analysis, the organism parameter was set to Homo sapiens with the highest confidence level of 0.900. The obtained results were gathered and imported into Cytoscape v3.9.1 (The Cytoscape Consortium, San Diego, CA, USA) for visualization and further analysis. To examine the top 10 core targets, the CytoHubba plugin was employed, using the number of degrees as the primary parameter.

### 3.8. Molecular Docking Study

The ligand structures (**10**, **11**, and Ravoxertinib) were prepared using ChemDraw Ultra 15.0 (Perkin Elmer, MA, USA). The protonation state of **10** and **11** were prepared using Open Babel GUI and Command Prompt with pH 7.4. To predict the potential binding interactions between the compounds and the core targets, an in silico assay was conducted using PyRx Virtual Screening Tools v0.8. The X-ray crystal structures of the proteins were obtained from the Protein Data Bank (https://www.rcsb.org/, accessed on 16 March 2023), e.g., 6GES for MAPK3 (ERK1) and 1WZY for MAPK1 (ERK2). Prior to the analysis, the proteins were prepared by removing water molecules and any other ligands attached to the main protein using Pymol v2.5 (https://pymol.org/2/, accessed on 16 March 2023). The free binding energies (ΔG), interaction types, and amino acid residues were calculated and analyzed using BIOVIA Discovery Studio Visualizer 2022 (Biovia, CA, USA).

### 3.9. Molecular Dynamics Study

The stability of the interactions between ligand **11** and the proteins was assessed using Yasara Dynamic, following the previously described methodology [29]. In summary, the optimal binding between ligand **11** and the proteins was selected for further analysis, incorporating the AMBER14 force field with standard parameters. The simulation was conducted at a temperature of 298 K and a pH of 7.4 for 15 ns. Two RMSD parameters were employed: RMSD ligand conformation and RMSD ligand movement. The evaluation utilized the default scripts: md_run.mcr and md_analyse.mcr. The RMSD plots were generated using the Rstudio program (https://posit.co/products/open-source/rstudio/, accessed on 17 March 2023).

## 4. Conclusions

Here, 4′-pyridinecarbonyl-substituted renieramycin-type derivatives were prepared from compound **4** using light-induced intramolecular photoredox reactions and Steglich esterification. The optimal conditions for the photoredox reaction were achieved by employing 4 W blue light and dichloromethane. The natural renieramycins (**4**–**6**) were successfully transformed into their corresponding derivatives (**7** and **8**, which contained a 1,3-dioxole-bridged phenolic moiety on ring A) in excellent–moderate yields (yields: 81%–37%). The attachment of the 4′-pyridinecarbonyl ester at the C-5 and C-22 positions was accomplished smoothly. Cytotoxicity study revealed that compounds **4** and **9** exhibited the most potent cytotoxicity against the human H292 and H460 NSCLC cell lines among the natural renieramycins and semisynthesized renieramycin-type derivatives, respectively. Interestingly, compound **11**, which contained both the 4′-pyridinecarbonyl ester at C-5 and the 1,3-dioxole-bridged phenolic motifs on ring A, showed better cytotoxicity than its parent compound (**7**). In addition, the renieramycin-type derivatives with a hydroxyl group at C-5 and C-22 lost their cytotoxicity against the NSCLC cells. Thus, the conformation of ring A featuring the 4′-pyridinecarbonyl ester at C-5 is crucial for the cytotoxic activity of renieramycins. Due to the limited quantity of the compound, a virtual network pharmacology study was conducted to simulate the target genes and molecular pathways. Molecular docking and dynamics studies predicted that the target proteins of compound **11** were MAPK1 and MAPK3. In future studies, the renieramycin-type derivatives will be further explored for their potential as anti-lung cancer agents.

## Figures and Tables

**Table 1:** Optimization of the conditions for the photoredox of natural renieramycins.

Entry	Starting Material	Light Source ^a^	Solvent	Renieramycin Product	Isolated Yield (%)
1	4	18 W	CH_2_Cl_2_	7	64
2	4	18 W	CHCl_3_	7	46
3	4	18 W	THF	7	47
4	4	4 W	CH_2_Cl_2_	7	81
5	4	4 W	CHCl_3_	7	51
6	4	4 W	THF	7	54
7	5	4 W	CH_2_Cl_2_	6:8 = 1:2 ^b^	18:37 ^c^
8	6	4 W	CH_2_Cl_2_	8	48

^a^ 18 W refers to the white light fluorescent lamp, and 4 W refers to the blue LED lamp. ^b^ Ratio was determined based on the proton signal of H-3 by nuclear magnetic resonance (NMR) spectroscopy analysis. ^c^ The yield of the major product was determined after column chromatographic purification.

**Table 2:** Cytotoxicity of the natural renieramycins and semisynthesized renieramycin derivatives against non-small-cell lung cancer cell lines.

Entry	Compound	5-*O*-Substituents	22-*O*-Substituents	IC_50_ ± S.D. (nM)	
				H292	H460
1	**3**	carbonyl	OH	97.85 ± 6.75	157.53 ± 6.65
2	**4**	carbonyl	angeloyl	35.36 ± 4.51	33.86 ± 2.16
3	**5**	carbonyl	angeloyl	170.03 ± 10.07	104.36 ± 22.02
4	**6**	carbonyl	angeloyl	111.74 ± 12.94	99.74 ± 0.13
5	**7**	OH	angeloyl	72.64 ± 2.55	83.32 ± 4.72
6	**8**	OH	angeloyl	183.37 ± 37.04	167.97 ± 3.53
7	**9**	carbonyl	4′-pyridinecarbonyl	3.52 ± 0.62	3.98 ± 0.38
8	**10**	4′-pyridinecarbonyl	angeloyl	33.79 ± 0.40	35.77 ± 2.11
9	**11**	4′-pyridinecarbonyl	angeloyl	35.27 ± 1.09	34.77 ± 2.19
10	**12**	OH	4′-pyridinecarbonyl	1.27 ± 0.20 µM	1.83 ± 0.83 µM
11	Cisplatin			4.23 ± 0.40 µM	3.86 ± 0.46 µM
12	Doxorubicin			37.54 ± 4.41	43.37 ± 5.60

## Data Availability

The data described and analyzed in this study are available from the corresponding author upon reasonable request.

## Supplementary Materials

The following supporting information can be downloaded at: https://www.mdpi.com/article/10.3390/md21070400/s1, Figures S1–S15: ^1^H NMR, ^13^C NMR and 2D NMR spectra of compounds **7**–**9** and **11**–**12**, Figures S16 and S17: Molecular docking of Ravoxertinib and the protonated **10** and **11** with both MAPK1 (ERK2) and MAPK3 (ERK1), Tables S1 and S2: Theoretical level of minimization of the 3D structures of **10** and **11**.
