# Supplementary material for: Light-Mediated Transformation of Renieramycins and Semisynthesis of 4′-Pyridinecarbonyl-Substituted Renieramycin-Type Derivatives as Potential Cytotoxic Agents against Non-Small-Cell Lung Cancer Cells
Source: Mar Drugs. 2023 Jul 13;21(7):400. doi: 10.3390/md21070400 (PMC10381490; doi:10.3390/md21070400)
Supplement: Supplementary file 1 [file marinedrugs-21-00400-s001.zip › marinedrugs-2495178-supplementary.pdf]

Supporting Information

Light-Mediated Transformation of Renieramycins and Semisynthesis of 4′-Pyridinecarbonyl-Substituted Renieramycin-Type Derivatives as Potential Cytotoxic Agents against Non-Small-Cell Lung Cancer Cells

*Suwimon Sinsook [1,2], Koonchira Buaban [1,3], Iksen Iksen [4], Korrakod Petsri [4,5], Bhurichaya Innets [4,5], Chaisak Chansriniyom [1,3], Khanit Suwanborirux [1,3], Masashi Yokoya [6], Naoki Saito [6], Varisa Pongrakhananon [4,7], Pithi Chanvorachote [4,5] and Supakarn Chamni [1,3,]**

[1] Department of Pharmacognosy and Pharmaceutical Botany, Faculty of Pharmaceutical Sciences, Chulalongkorn University, Bangkok 10330, Thailand; nhamsuwimon1997@gmail.com (S.S.); koonchi-ra.buaban@gmail.com (K.B.); chaisak.c@pharm.chula.ac.th (C.C.); khanit.s@chula.ac.th (K.S.)

[2] Pharmaceutical Sciences and Technology Program, Faculty of Pharmaceutical Sciences, Chulalongkorn University, Bangkok 10330, Thailand

[3] Natural Products and Nanoparticles Research Unit (NP2), Chulalongkorn University, Bangkok 10330, Thailand

[4] Department of Pharmacology and Physiology, Faculty of Pharmaceutical Sciences, Chulalongkorn University, Bangkok, 10330, Thailand; ikseniksen08@gmail.com (I.I.); korrakod.petsri@gmail.com (K.P.); 6481004120@student.chula.ac.th (B.I.); varisa.p@pharm.chula.ac.th (V.P.); pithi.c@chula.ac.th (P.C.)

[5] Center of Excellence in Cancer Cell and Molecular Biology, Faculty of Pharmaceutical Sciences, Chulalongkorn University, Bangkok, 10330, Thailand

[6] Graduate School of Pharmaceutical Sciences, Meiji Pharmaceutical University, 2-522-1 Noshio, Kiyose, Tokyo 204-8588, Japan; yokoya@my-pharm.ac.jp (M.Y.); naoki@my-pharm.ac.jp (N.S.)

[7] Preclinical Toxicity and Efficacy, Assessment of Medicines and Chemicals Research Unit, Chulalongkorn University, Bangkok, 10330 Thailand

* Correspondence: supakarn.c@pharm.chula.ac.th; Tel.: +662-218-8357

Table of Contents

Physical and spectroscopic data of **7** 5

Physical and spectroscopic data of **8** 6

Physical and spectroscopic data of **9** 7

Physical and spectroscopic data of **11** 11

Physical and spectroscopic data of **12** 15

Theoretical level of minimization of the 3D structures of **10**.......... 18

Theoretical level of minimization of the 3D structures of **11**.......... 29

Molecular docking of Ravoxertinib with both MAPK1 (ERK2).......... 41
and MAPK3 (ERK1)

Molecular docking of the protonation state of **10** and **11** 42
with both MAPK1 (ERK2) and MAPK3 (ERK1)

List of Figures

Figure S1. ^{1}H NMR (400 MHz) spectrum of **9** in $CDCl_3$. 8

Figure S2. ^{13}C NMR (100 MHz) spectrum of **9** in $CDCl_3$. 8

Figure S3. COSY (400 MHz) spectrum of **9** in $CDCl_3$ 9

Figure S4. HSQC (400 MHz) spectrum of **9** in $CDCl_3$ 9

Figure S5. HMBC (400 MHz) spectrum of **9** in $CDCl_3$ 10

Figure S6. ^{1}H NMR (400 MHz) spectrum of **11** in $CDCl_3$ 12

Figure S7. ^{13}C NMR (100 MHz) spectrum of **11** in $CDCl_3$ 12

Figure S8. COSY (400 MHz) spectrum of **11** in $CDCl_3$ 13

Figure S9. HSQC (400 MHz) spectrum of **11** in $CDCl_3$ 13

Figure S10. HMBC (400 MHz) spectrum of **11** in $CDCl_3$ 14

Figure S11. ^{1}H NMR (400 MHz) spectrum of **12** in $CDCl_3$ 16

Figure S12. ^{13}C NMR (100 MHz) spectrum of **12** in $CDCl_3$ 16

Figure S13. COSY (400 MHz) spectrum of **12** in $CDCl_3$ 17

Figure S14. HSQC (400 MHz) spectrum of **12** in $CDCl_3$ 17

Figure S15. HMBC (400 MHz) spectrum of **12** in $CDCl_3$ 18

Figure S16. Molecular docking Ravoxertinib 40
with both MAPK1 (ERK2) and MAPK3 (ERK1).

Figure S17. Molecular docking of the protonation state of **10** and **11** 41
with both MAPK1 (ERK2) and MAPK3 (ERK1)

List of Table

Table S1. Theoretical level of minimization of the 3D structures of **10**........ 18

Table S2. Theoretical level of minimization of the 3D structures of **11**……………………29

Physical and spectroscopic data of **7**

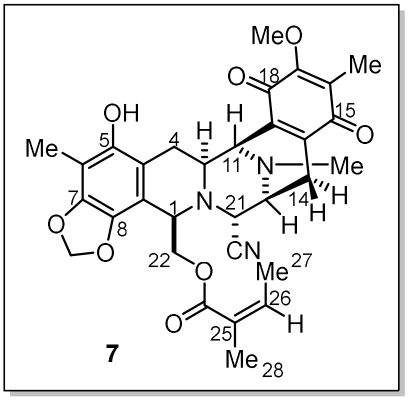

renieramycin T (**7**): The compound **7** was synthesized from **4** (15 mg, 0.03 mmol) in dry CH_2Cl_2 (20 mL) was irradiated under 4 W LED lamp (Blue light) to obtain **7**; yield 81%; yellow amorphous powder; UV (MeOH) λ_{max} (log ε) 268 (3.80), 370 (2.72) nm; ^{1}H NMR ($CDCl_3$, 400 MHz) δ in ppm: 6.00 (1H, qq, J = 7.2, 1.5 Hz, 26-H), 5.88 (2H, d, J = 26.8 Hz, OCH_2O), 4.46 (1H, br s, 5-OH), 4.42 (1H, dd, J = 11.2, 3.6 Hz, 22-H_β), 4.16 (1H, overlapped, 1-H), 4.11 (1H, d, J = 2.4 Hz, 21-H), 4.01 (1H, overlapped, 11-H), 3.99 (3H, s, 17-OCH_3), 3.98 (1H, overlapped, 22-H_α), 3.38 (1H, d, J = 6.8 Hz, 13-H), 3.25 (1H, br d, J = 11.6 Hz, 3-H), 2.87 (1H, br d, J = 15.2 Hz, 4-H_α), 2.77 (1H, dd, J = 17.2, 6.8 Hz, 14-H_α), 2.33 (1H, overlapped, 14-H_β), 2.29 (3H, s, NCH_3), 2.11 (3H, s, 6-CH_3), 1.94 (3H, s, 16-CH_3), 1.85 (3H, dq, J = 7.2, 1.5 Hz, 27-CH_3), 1.69 (3H, s, 28-CH_3), 1.67 (1H, overlapped, 4-H_β); ^{13}C NMR ($CDCl_3$, 100 MHz) δ in ppm: 186.1 (C-15), 182.8 (C-18), 167.1 (C-24), 155.4 (C-17), 144.9 (C-7), 144.7 (C-5), 141.7 (C-20), 139.8 (C-26), 136.9 (C-8), 135.8 (C-19), 129.0 (C-16), 126.8 (C-25), 117.5 (21-CN), 113.1 (C-10), 112.2 (C-9), 106.1 (C-6), 101.1 (OCH_2O), 64.6 (C-22), 61.0 (17-OCH_3), 59.7 (C-21), 56.4 (C-1), 56.3 (C-3), 55.0 (C-11), 54.8 (C-13), 41.5 (NCH_3), 26.8 (C-4), 21.2 (C-14), 20.5 (28-CH_3), 15.8 (27-CH_3), 8.8 (6-CH_3), 8.7 (16-CH_3); HRESIMS *m/z* 576.2344 ($[M+H]^+$, calculated for $C_{31}H_{34}N_3O_8$, 576.2347).

Physical and spectroscopic data of **8**

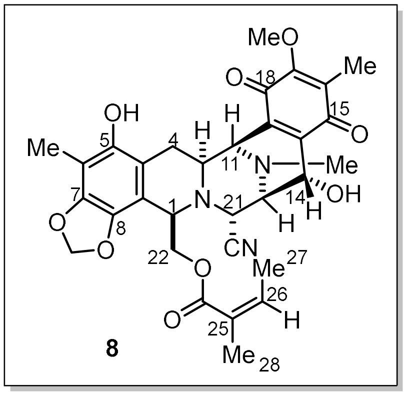

renieramycin U (**8**): The compound **8** was synthesized from **6** (40 mg, 0.07 mmol) in dry CH_2Cl_2 (20 mL) was irradiated under 4 W LED lamp (Blue light) to obtain **8**; yield 48%; yellow amorphous powder; $[\alpha]_D^{25}$ −5.4 (*c* 0.71, $CHCl_3$) (lit. $[\alpha]_D^{23}$ −51.3 (*c* 0.07, $CHCl_3$)[(1)]; ECD Δ*ε* (*c* 240.02 μM, methanol, 20 °C) −0.5 (296), −5.5 (280), −12.7 (273), +5.9 (264), +5.0 (260), −10.5 (245), +18.2 (230), +26.2 (219), +6.3 (214), +13.9 (209), +7.6 (198) nm; IR (ATR) ν_{max} 3384 (br), 2926, 1714, 1651, 1455, 1230, 1147, 1093, 1045, 759 cm^{-1}; UV (MeOH) λ_{max} (log *ε*) 225 (4.11), 269 (4.04) nm; ^{1}H NMR ($CDCl_3$, 400 MHz) δ in ppm: 6.04 (1H, qq, *J* = 7.0, 1.0 Hz, 26-H), 5.95 (1H, d, *J* = 1.0 Hz, OCH_2O), 5.88 (1H, d, *J* = 1.0 Hz, OCH_2O), 4.43 (1H, dd, *J* = 11.3, 3.2 Hz, 22-H_β), 4.39 (1H, br d, *J* = 7.0 Hz, 14-H), 4.24 (1H, dd, *J* = 7.0, 2.4 Hz, 21-H), 4.16 (1H, dd, *J* = 4.9, 3.2 Hz, 1-H), 4.11 (1H, br d, *J* = 2.3 Hz, 11-H), 4.06 (3H, s, 17-OCH_3), 3.96 (1H, dd, *J* = 11.3, 5.2 Hz, 22-H_α), 3.52 (1H, br s, 14-OH), 3.43 (1H, dd, *J* = 7.0, 2.4 Hz, 13-H), 3.22 (1H, dt, *J* = 12.1, 2.3 Hz, 3-H), 2.88 (1H, dd, *J* = 15.0, 2.3 Hz, 4-H_α), 2.49 (3H, s, NCH_3), 2.13 (3H, s, 6-CH_3), 1.98 (3H, s, 16-CH_3), 1.91 (3H, dq, *J* = 7.0, 1.0 Hz, 27-CH_3), 1.71 (3H, s, 28-CH_3), 1.62 (1H, overlapped, 4-H_β); ^{13}C NMR ($CDCl_3$, 100 MHz) δ in ppm: 188.0 (C-15), 183.0 (C-18), 167.1 (C-24), 155.8 (C-17), 144.7 (C-7), 141.0 (C-5), 140.7 (C-20), 140.0 (C-8), 140.0 (C-26), 136.0 (C-19), 129.0 (C-16), 126.7 (C-25), 119.4 (C-10), 116.9 (21-CN), 112.0 (C-6), 112.0 (C-9), 101.2 (OCH_2O), 64.6 (C-22), 62.7 (C-13), 62.3 (C-14), 61.1 (17-OCH_3), 57.6 (C-21), 56.6 (C-1), 55.8 (C-11), 55.5 (C-3), 42.4 (NCH_3), 26.7 (C-4), 20.5 (28-CH_3), 15.8 (27-CH_3), 8.8 (6-CH_3), 8.5 (16-CH_3); HRESIMS *m/z* 592.2290 ($[M+H]^+$, calculated for $C_{31}H_{34}N_3O_9$, 592.2290).

Physical and spectroscopic data of **9**

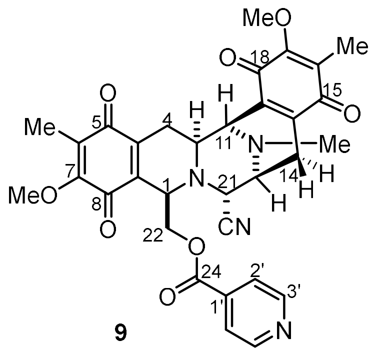

9

22-O-(4'-pyridinecarbonyl) jorunnamycin A (**9**): The compound **9** was synthesized from **3** (25 mg, 0.05 mmol), DMAP (31 mg, 0.25 mmol), EDCI (49 mg, 0.25 mmol) and isonicotinoyl chloride hydrochloride (45 mg, 0.25 mmol) in dry CH_2Cl_2 (10 mL) to obtain **9**; yield 72%; yellow amorphous powder; ^{1}H NMR ($CDCl_3$, 400 MHz) δ in ppm: 8.68 (2H, d, J = 4.4 Hz, 2'-H), 7.47 (2H, d, J = 4.4 Hz, 3'-H), 4.99 (1H, br d, J = 11.2 Hz, 22-H_α), 4.16 (1H, d, J = 11.2 Hz, 22-H_β), 4.09 (2H, s, 1-H & 21-H), 4.04 (3H, s, 7-OCH_3), 3.95 (1H, s, 11-H), 3.76 (3H, s, 17-OCH_3), 3.40 (1H, d, J = 7.2 Hz, 13-H), 3.10 (1H, d, J = 11.2 Hz, 3-H), 2.92 (1H, d, J = 17.2 Hz, 4-H_α), 2.71 (1H, dd, J = 20.8, 7.2 Hz, 14-H_α), 2.34 (1H, d, J = 20.8 Hz, 14-H_β), 2.23 (3H, s, NCH_3), 1.99 (3H, s, 6-CH_3), 1.73 (3H, s, 16-CH_3), 1.24 (1H, overlapped, 4-H_β); ^{13}C NMR ($CDCl_3$, 100 MHz) δ in ppm: 185.9 (C-15), 185.4 (C-5), 182.1 (C-18), 181.1 (C-8), 164.0 (C-24), 155.6 (C-7), 154.9 (C-17), 150.2 (2 × C-2'), 142.1 (C-10), 142.1 (C-20), 136.7 (C-1'), 135.1 (C-9), 134.5 (C-19), 128.9 (C-6), 128.1 (C-16), 122.7 (2 × C-3'), 116.8 (21-CN), 62.9 (C-22), 61.2 (17-OCH_3), 60.9 (7-OCH_3), 58.4 (C-21), 56.5 (C-1), 54.4 (C-13), 54.3 (C-3), 54.1 (C-11), 41.4 (NCH_3), 25.5 (C-4), 21.0 (C-14), 8.9 (6-CH_3), 8.7 (16-CH_3).

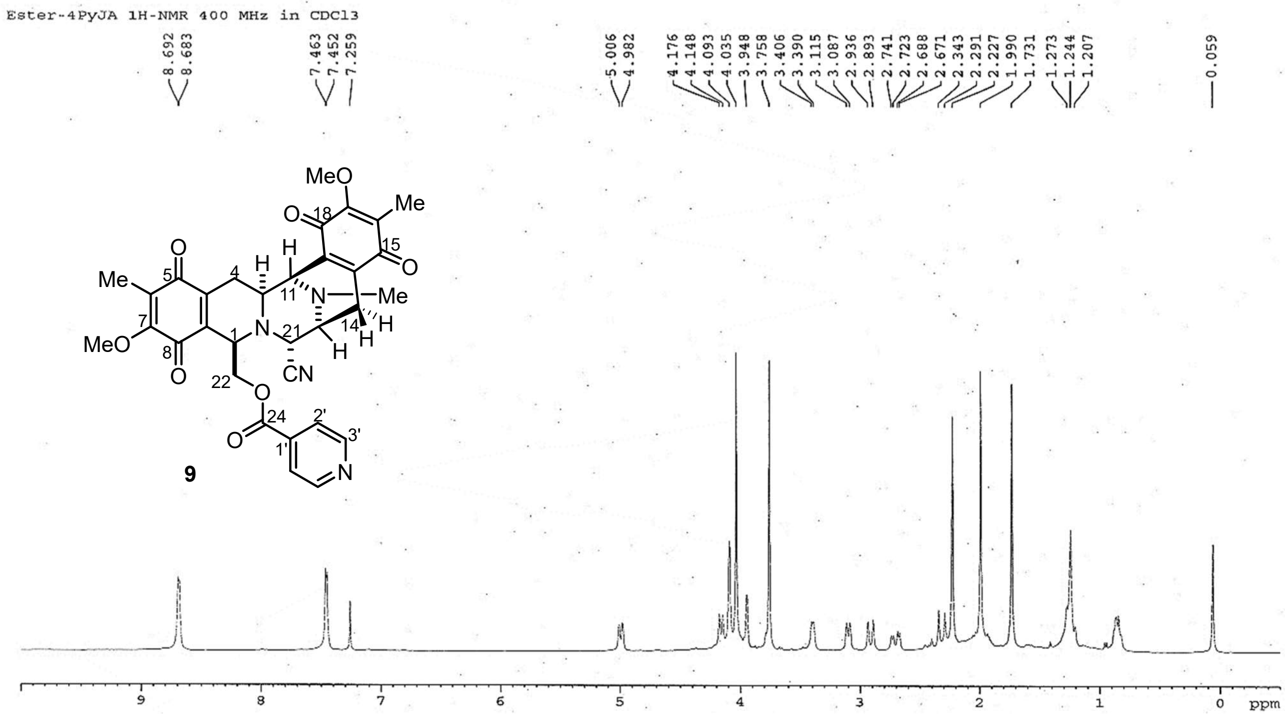

Figure S1. ^{1}H NMR (400 MHz) spectrum of **9** in $CDCl_3$.

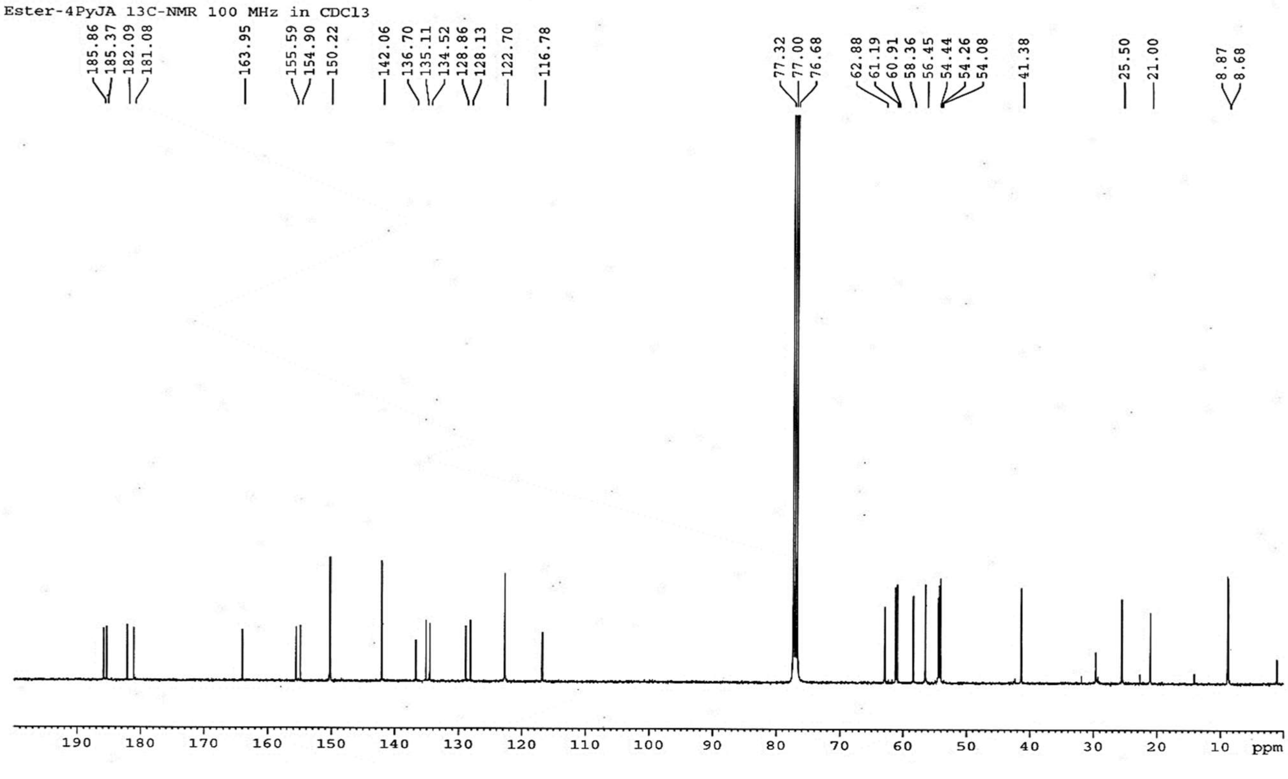

Figure S2. ^{13}C NMR (100 MHz) spectrum of **9** in $CDCl_3$.

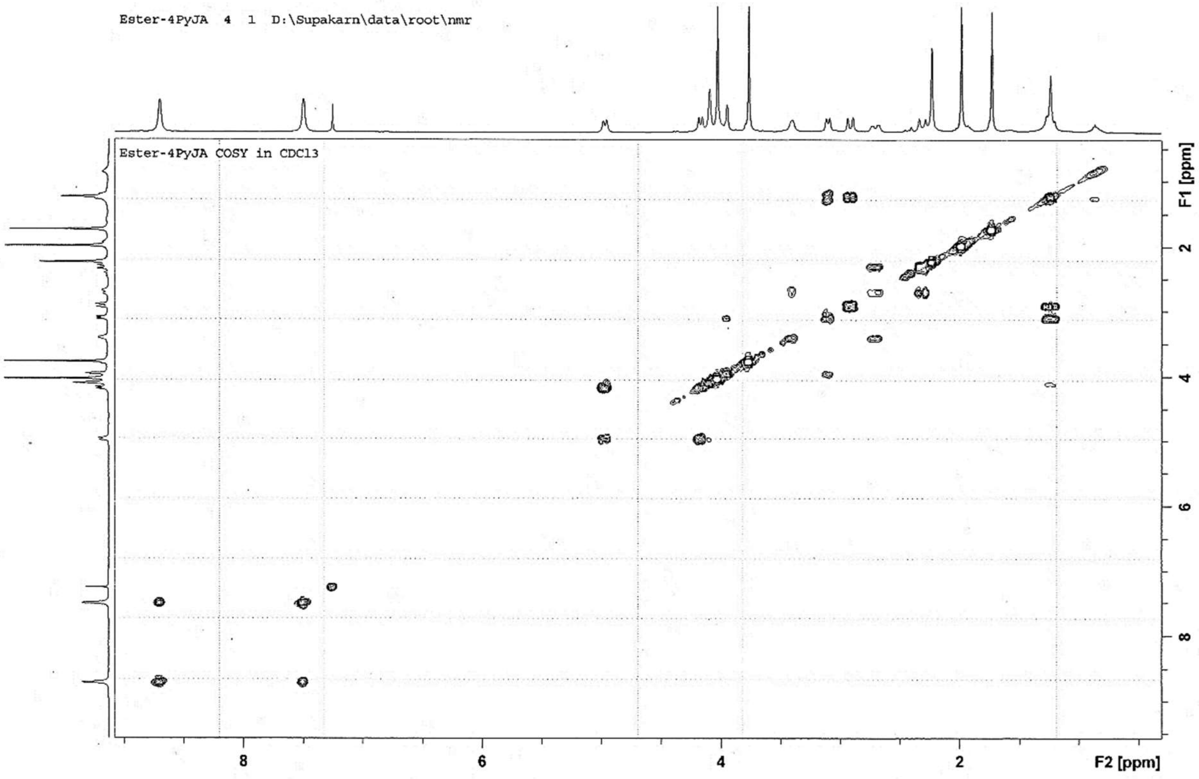

Figure S3. COSY (400 MHz) spectrum of **9** in $CDCl_3$

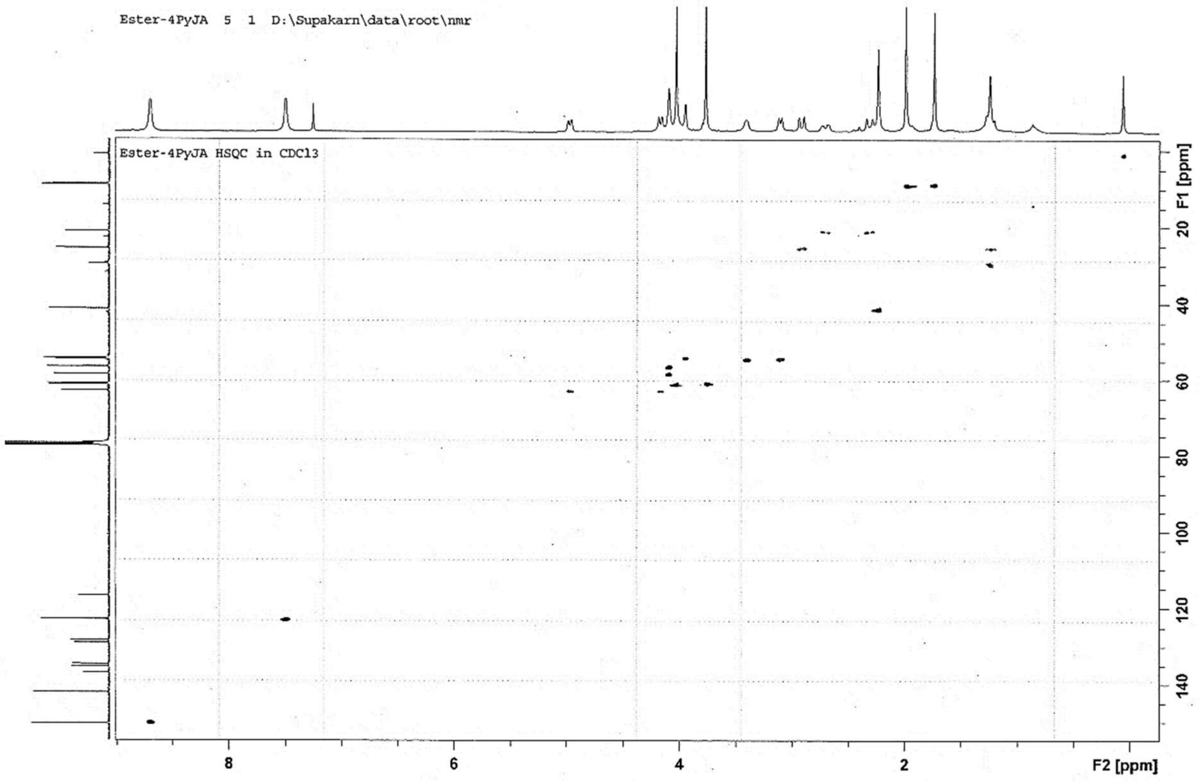

Figure S4. HSQC (400 MHz) spectrum of **9** in $CDCl_3$

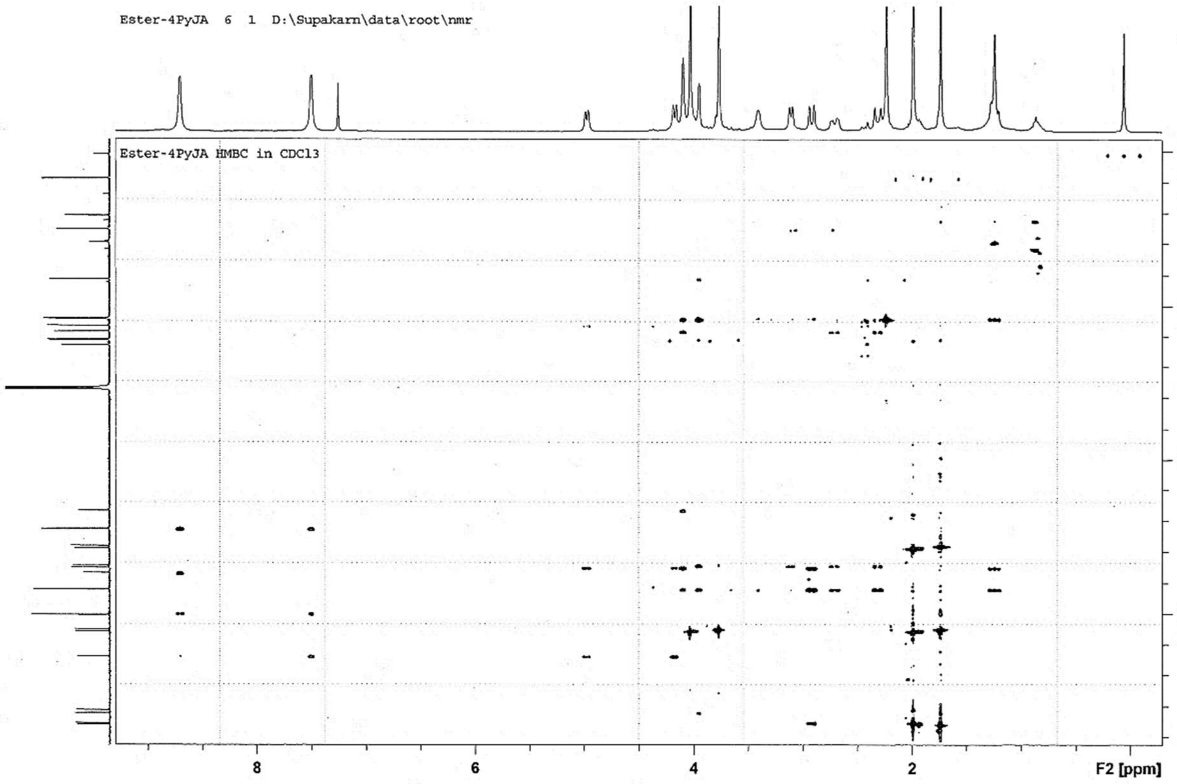

Figure S5. HMBC (400 MHz) spectrum of **9** in $CDCl_3$

Physical and spectroscopic data of **11**

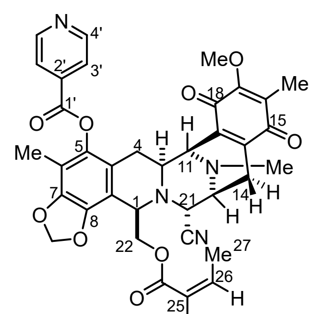

11

5-O-(4'-pyridinecarbonyl) renieramycin T (**11**): The compound **11** was synthesized from **7** (21 mg, 0.04 mmol), DMAP (5 mg, 0.04 mmol), EDCI (8 mg, 0.04 mmol) and isonicotinoyl chloride hydrochloride (32 mg, 0.18 mmol) in dry CH_2Cl_2 (20 mL) to obtain **11**; yield 20%; yellow amorphous powder; $[\alpha]_D^{25}$ +6.1 (*c* 0.56, $CHCl_3$); ECD $\Delta\varepsilon$ (*c* 15.28 μM, methanol, 20 °C) −3.0 (302), −13.0 (290), +9.4 (278), +50.7 (264), +0.5 (236), −59.7 (224), −84.5 (219), +41.3 (210), −50.9 (205), +47.6 (203) nm; IR (ATR) ν_{max} 3446 (br), 2929, 1747, 1709, 1652, 1455, 1234, 1150, 1108, 1026, 755 cm^{-1}; ^{1}H NMR ($CDCl_3$, 400 MHz) δ in ppm: 8.94 (2H, d, *J* = 5.6 Hz, 4'-H), 8.06 (2H, d, *J* = 5.6 Hz, 3'-H), 6.02 (2H, d, *J* = 26.8 Hz, OCH_2O), 6.01 (1H, overlapped, 26-H), 4.61 (1H, dd, *J* = 11.6, 2.8 Hz, 22-H_α), 4.20 (1H, br t, *J* = 4.0 Hz, 1-H), 4.14 (1H, d, *J* = 2.4 Hz, 21-H), 4.05 (1H, dd, *J* = 11.6, 4.0 Hz, 22-H_β), 3.92 (1H, d, *J* = 2.4 Hz, 11-H), 3.73 (3H, s, 17-OCH_3), 3.38 (1H, dd, *J* = 7.2, 2.4 Hz, 13-H), 3.26 (1H, dt, *J* = 12.0, 2.4 Hz, 3-H), 2.73 (1H, dd, *J* = 20.8, 7.2 Hz, 14-H_α), 2.53 (1H, dd, *J* = 15.2, 1.6 Hz, 4-H_α), 2.33 (1H, d, *J* = 20.8 Hz, 14-H_β), 2.25 (3H, s, NCH_3), 2.05 (3H, s, 6-CH_3), 1.89 (3H, s, 16-CH_3), 1.88 (3H, dq, *J* = 7.2, 1.2 Hz, 27-CH_3), 1.68 (3H, dq, *J* = 1.4, 1.2 Hz, 28-CH_3), 1.61 (1H, overlapped, 4-H_β); ^{13}C NMR ($CDCl_3$, 100 MHz) δ in ppm: 185.8 (C-15), 182.6 (C-18), 167.0 (C-24), 162.7 (C-1'), 155.2 (C-17), 150.0 (2 × C-4'), 145.1 (C-7), 141.6 (C-20), 141.2 (C-8), 140.4 (C-26), 140.0 (C-5), 137.1 (C-2'), 135.2 (C-19), 128.8 (C-16), 126.6 (C-25), 123.5 (2 × C-3'), 120.1 (C-6), 117.2 (21-CN), 112.6 (C-10), 111.9 (C-9), 101.9 (OCH_2O), 63.0 (C-22), 60.4 (17-OCH_3), 58.9 (C-21), 56.5 (C-1), 55.3 (C-3), 54.7 (C-11), 54.5 (C-13), 41.4 (NCH_3), 28.0 (C-4), 21.0 (C-14), 20.5 (28-CH_3), 15.9 (27-CH_3), 9.5 (6-CH_3), 8.6 (16-CH_3); HRESIMS *m/z* 681.2554 ($[M+H]^+$, calculated for $C_{37}H_{37}N_4O_9$, 681.2555).

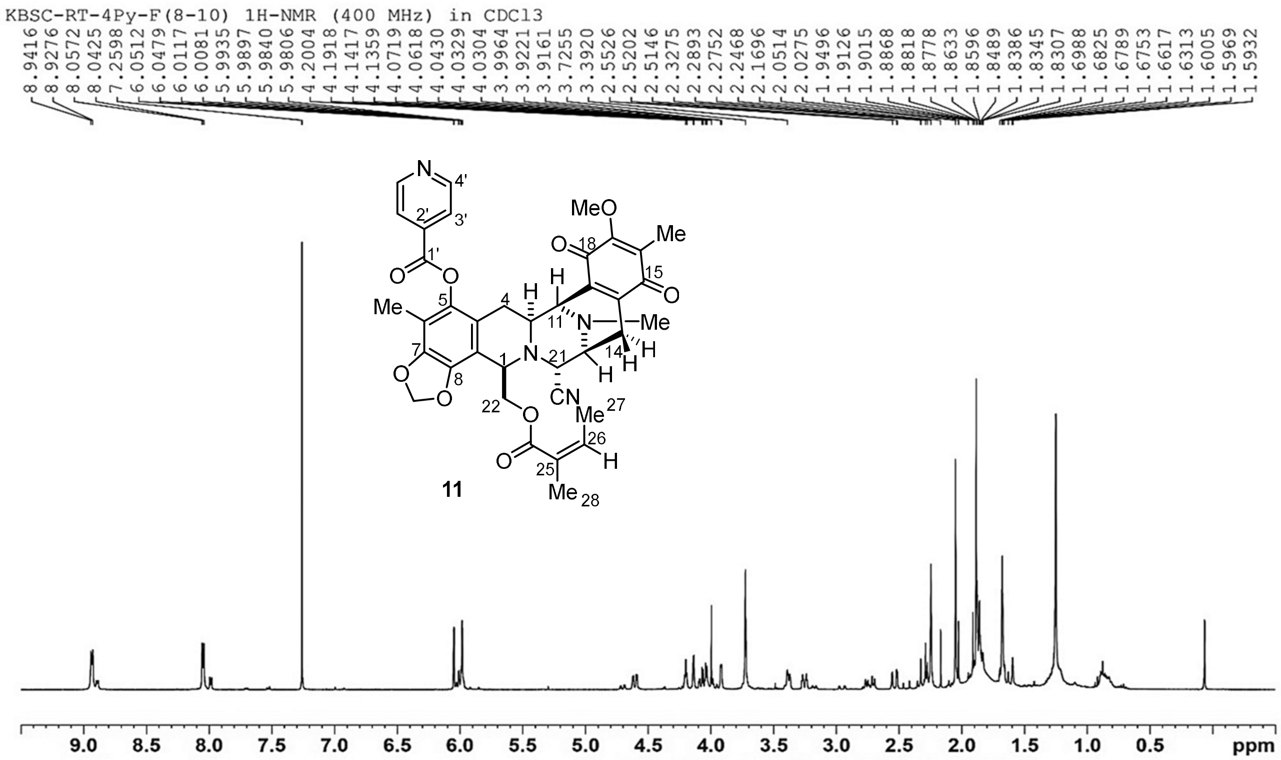

Figure S6. ^{1}H NMR (400 MHz) spectrum of **11** in $CDCl_3$

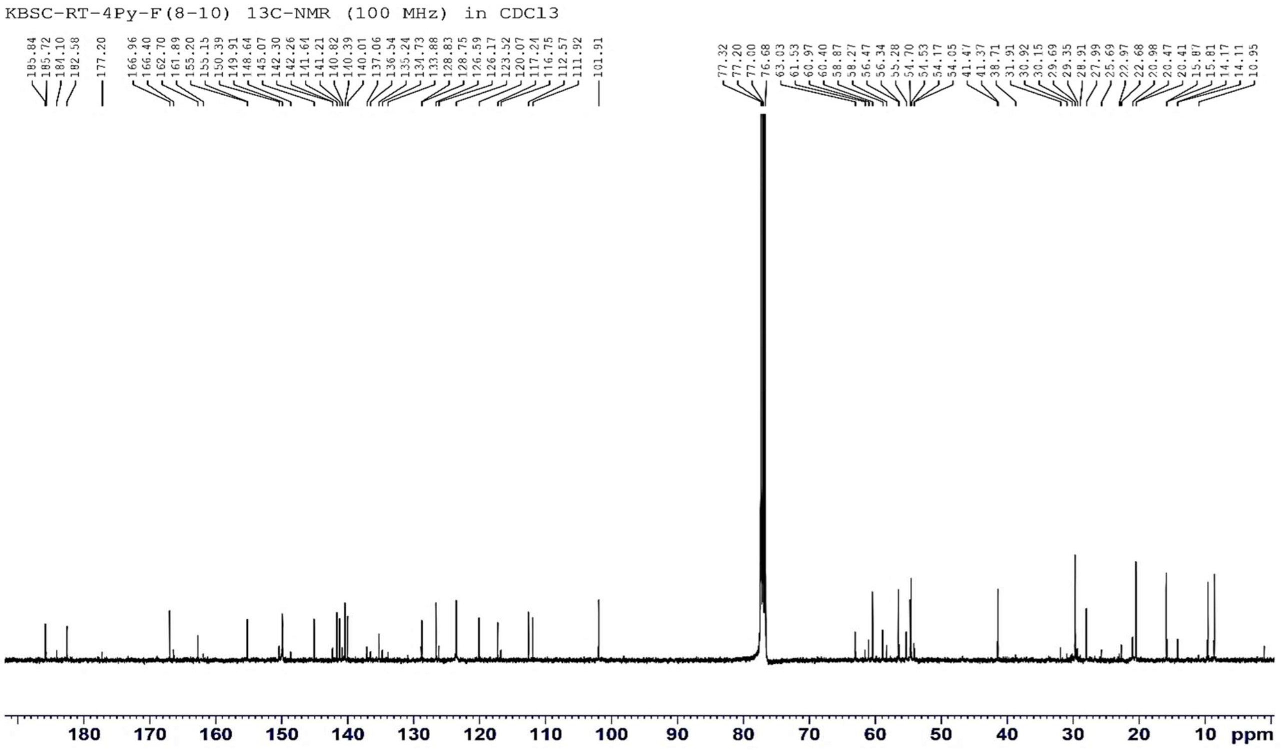

Figure S7. ^{13}C NMR (100 MHz) spectrum of **11** in $CDCl_3$

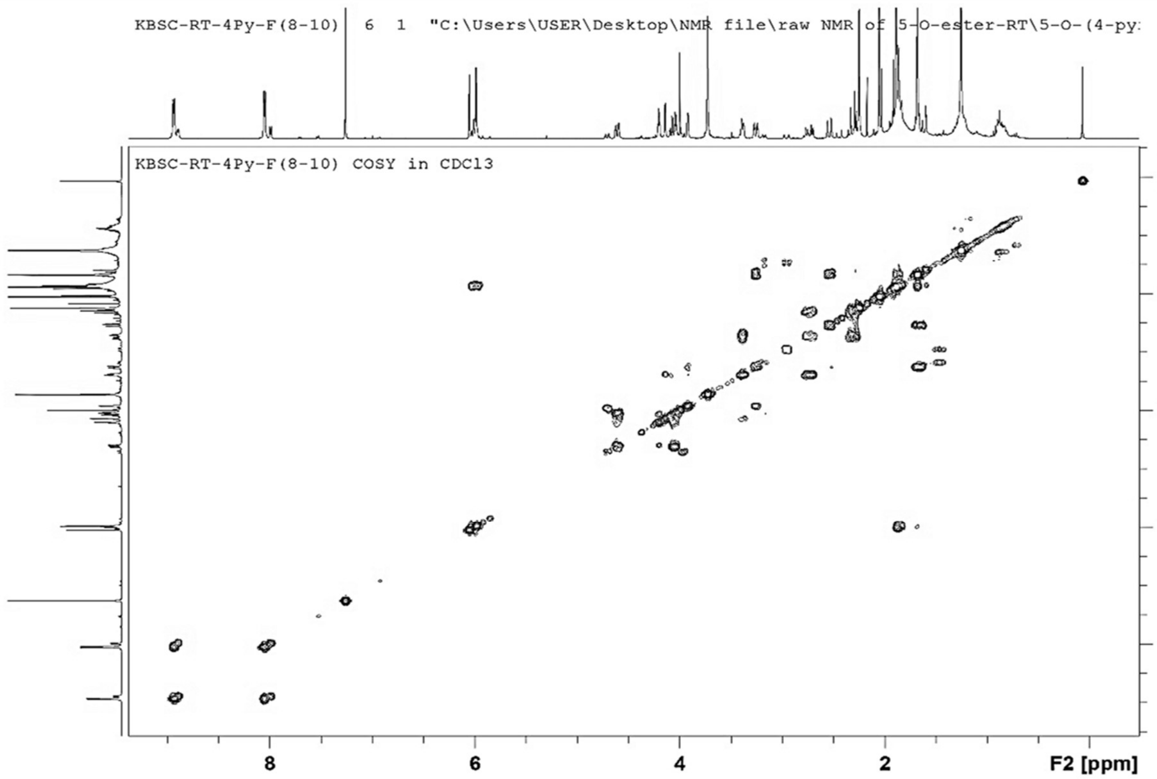

Figure S8. COSY (400 MHz) spectrum of **11** in $CDCl_3$

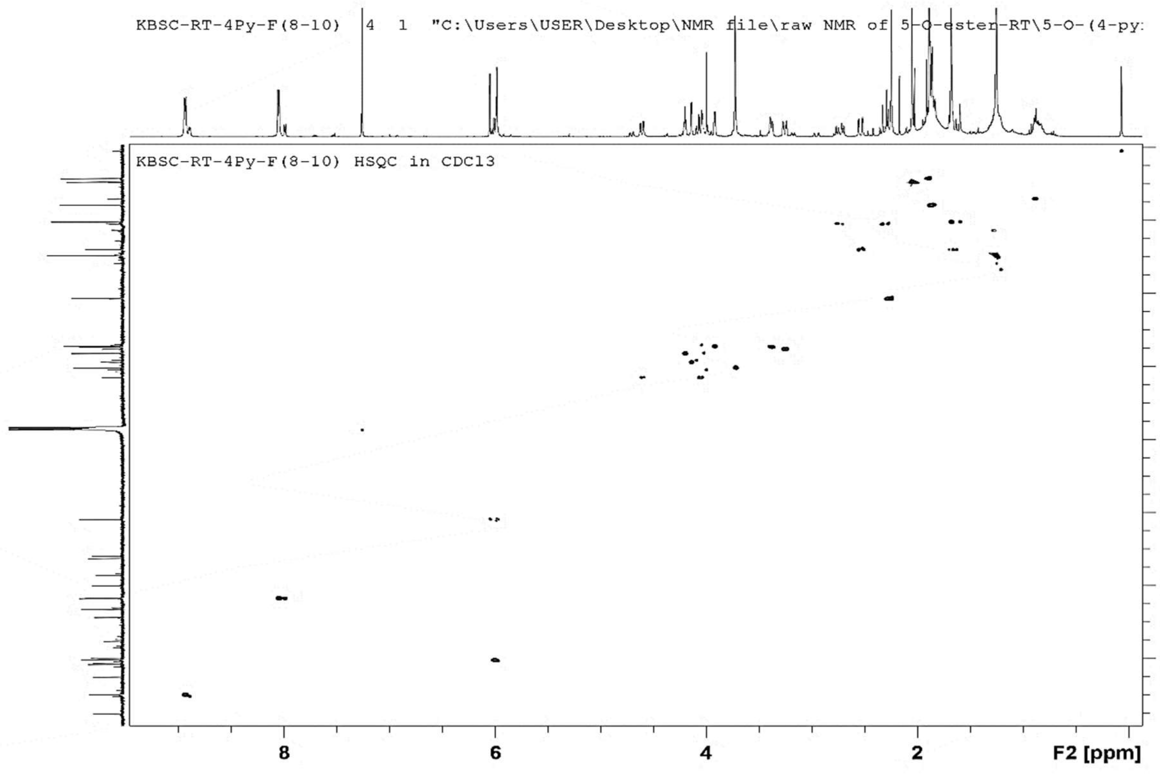

Figure S9. HSQC (400 MHz) spectrum of **11** in $CDCl_3$

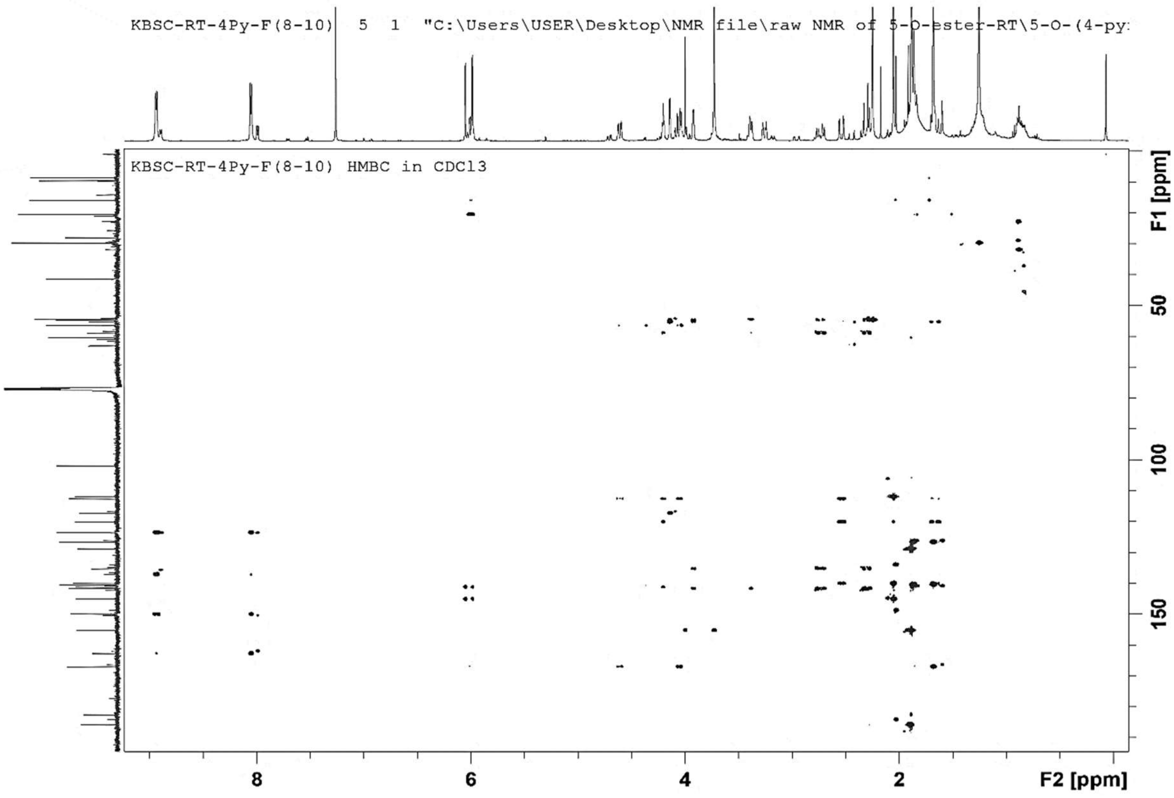

Figure S10. HMBC (400 MHz) spectrum of **11** in $CDCl_3$

Physical and spectroscopic data of **12**

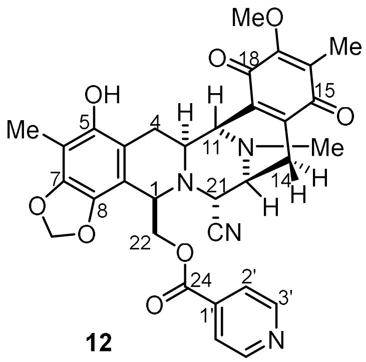

12

22-O-(4'-pyridinecarbonyl) renieramycin T (**12**): The compound **12** was synthesized from **9** (6 mg, 0.01 mmol) in dry CH_2Cl_2 (15 mL) was irradiated under 4 W LED lamp (Blue light) to obtain **12**; yield 83%; yellow amorphous powder; $[\alpha]_D^{25}$ −20.4 (*c* 0.37, $CHCl_3$); ECD $\Delta\varepsilon$ (*c* 247.24 μM, methanol, 20 °C) −8.1 (345), −6.5 (307), +7.3 (285), +11.5 (282), +2.0 (264), −3.9 (252), −5.5 (242), +0.3 (228), −10.9 (210) nm; IR (ATR) ν_{max} 3279 (br), 2923, 1731, 1651, 1615, 1409, 1374, 1277, 1260, 1233, 1091, 1026, 953, 703 cm^{-1}; UV (MeOH) λ_{max} (log ε) 211 (4.02), 272 (3.81) nm; ^{1}H NMR ($CDCl_3$, 400 MHz) δ in ppm: 8.71 (2H, dd, *J* = 4.6, 1.4 Hz, 3'-H), 7.56 (2H, dd, *J* = 4.6, 1.4 Hz, 2'-H), 5.88 (2H, d, *J* = 18.2 Hz, OCH_2O), 4.83 (1H, dd, *J* = 11.2, 3.2 Hz, 22-H_α), 4.24 (1H, overlapped, 21-H), 4.20 (1H, dd, *J* = 11.2, 3.2 Hz, 22-H_β), 4.07 (1H, d, *J* = 2.6 Hz, 11-H), 3.96 (1H, d, *J* = 3.2 Hz, 1-H), 3.80 (3H, s, 17-OCH_3), 3.38 (1H, br d, *J* = 7.6 Hz, 13-H), 3.24 (1H, dt, *J* = 12.1, 2.6 Hz, 3-H), 2.89 (1H, dd, *J* = 15.1, 1.8 Hz, 4-H_α), 2.70 (1H, dd, *J* = 20.8, 7.6 Hz, 14-H_α), 2.29 (1H, dd, *J* = 20.8, 1.4 Hz, 14-H_β), 2.26 (3H, s, NCH_3), 2.16 (3H, s, 16-CH_3), 1.79 (3H, s, 6-CH_3), 1.55 (1H, dd, *J* = 15.1, 12.1 Hz, 4-H_β); ^{13}C NMR ($CDCl_3$, 100 MHz) δ in ppm: 186.1 (C-15), 182.5 (C-18), 164.2 (C-24), 155.1 (C-17), 149.8 (2 × C-3'), 145.2 (C-20), 144.8 (C-8), 141.8 (C-5), 137.7 (C-1'), 136.8 (C-7), 135.3 (C-19), 128.6 (C-16), 123.1 (2 × C-2'), 117.2 (21-CN), 113.2 (C-6), 111.8 (C-9), 111.8 (C-10), 101.2 (OCH_2O), 64.4 (C-22), 60.9 (17-OCH_3), 59.2 (C-11), 56.9 (C-21), 56.1 (C-3), 54.7 (C-1), 54.7 (C-13), 41.4 (NCH_3), 27.0 (C-4), 21.1 (C-14), 8.9 (6-CH_3), 8.8 (16-CH_3); HRESIMS *m/z* 599.2134 ($[M+H]^+$, calculated for $C_{32}H_{31}N_4O_8$, 599.2136).

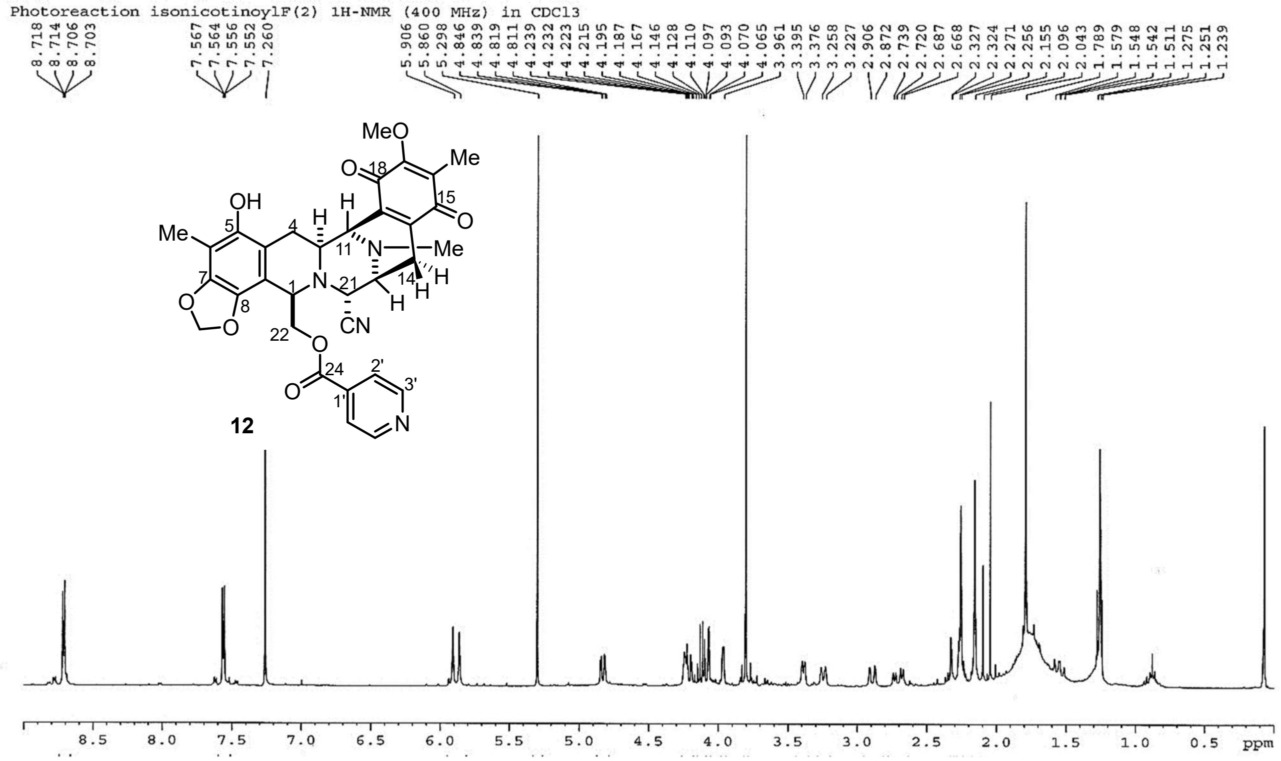

Figure S11. ^{1}H NMR (400 MHz) spectrum of **12** in $CDCl_3$

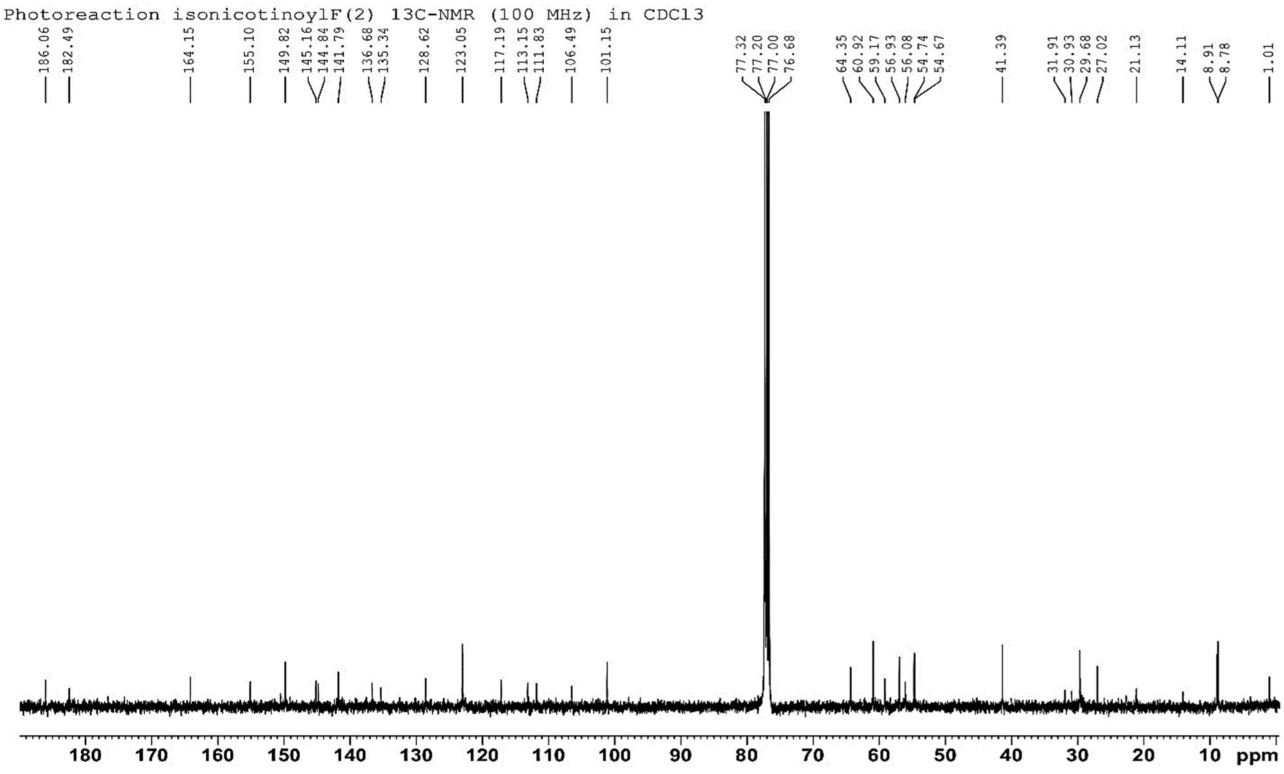

Figure S12. ^{13}C NMR (100 MHz) spectrum of **12** in $CDCl_3$

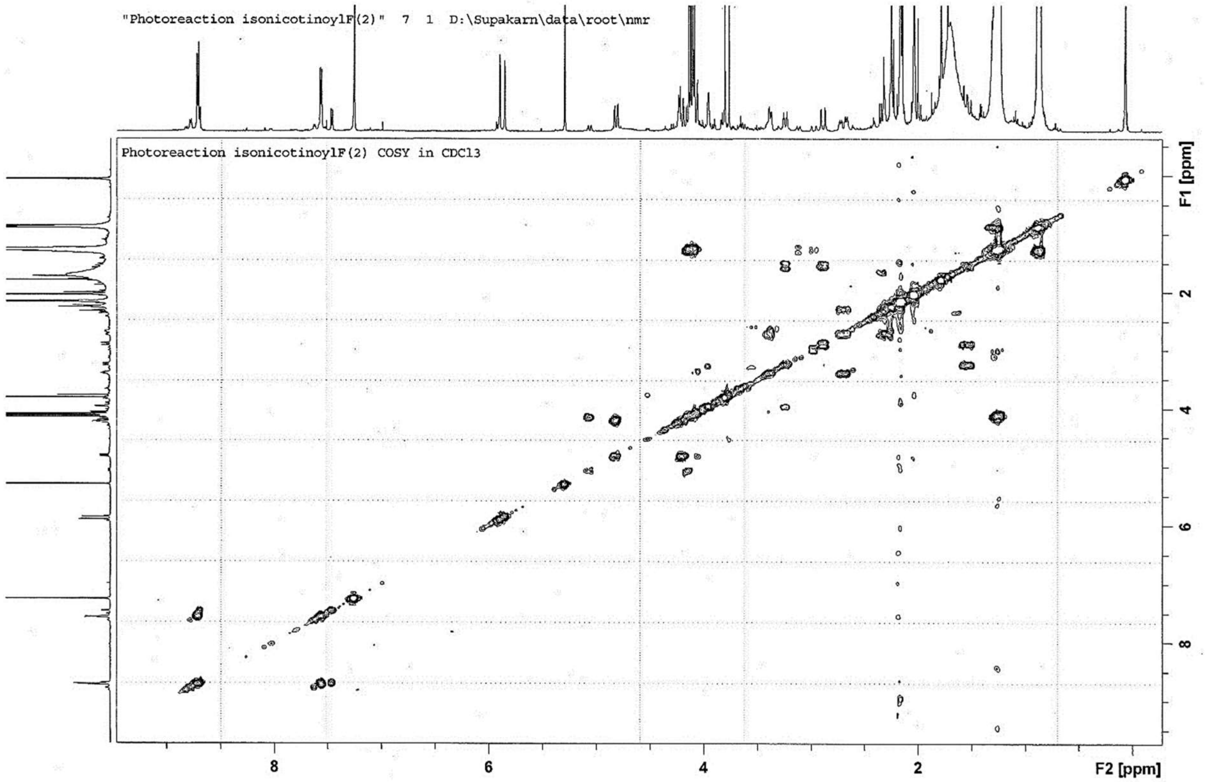

Figure S13. COSY (400 MHz) spectrum of **12** in $CDCl_3$

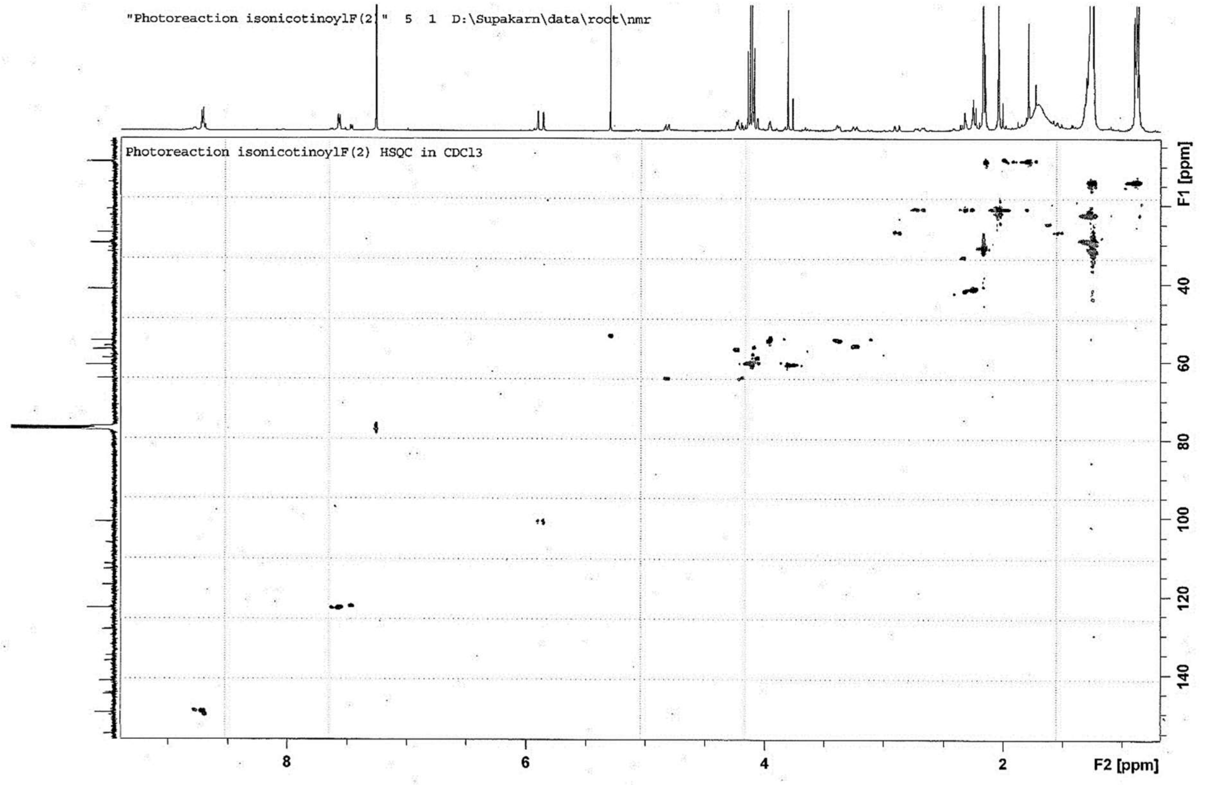

Figure S14. HSQC (400 MHz) spectrum of **12** in $CDCl_3$

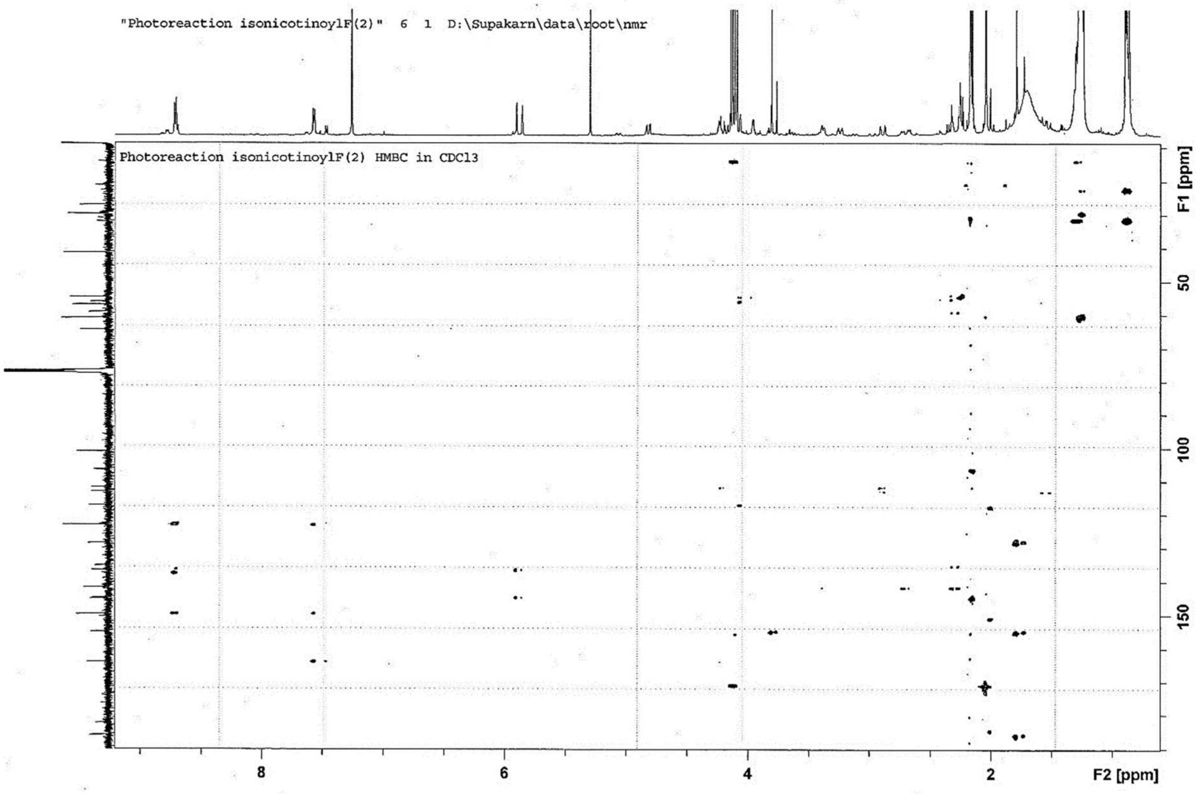

Figure S15. HMBC (400 MHz) spectrum of **12** in $CDCl_3$

Table S1. Theoretical level of minimization of the 3D structures of **10**

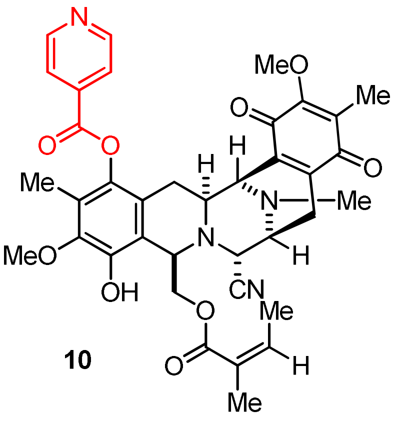

10

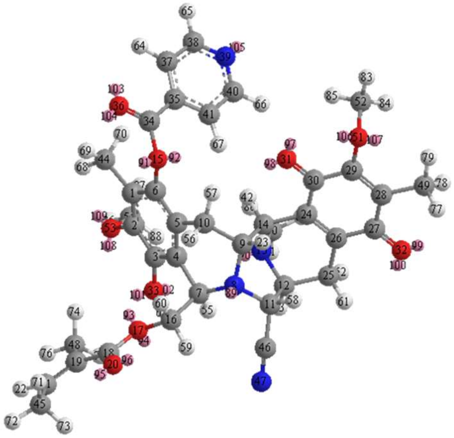

Atom	Atom Type (MM2)	Charge (MM2)	Charge (Huckel)	Bond lengths			Bond Angles			Dihedral angles		Close contacts	
				Atoms	Actual (° / Å)	Optimal (° / Å)	Atoms	Actual (° / Å)	Optimal (° / Å)	Atoms	Actual (° / Å)	Atoms	Actual (° / Å)
C(1)	C Alkene	0	-0.0800861	O(53)-Lp(109)	0.5955	0.6000	H(85)-C(53)-H(84)	110.0456	109.0000	C(29)-O(52)-C(53)-H(83)	-178.7248	Lp(109)-H(82)	1.9605
C(2)	C Alkene	0	0.166602	O(53)-Lp(108)	0.5999	0.6000	H(85)-C(53)-H(83)	109.0366	109.0000	C(29)-O(52)-C(53)-H(84)	-59.6448	C(28)-Lp(109)	2.2884
C(3)	C Alkene	0	0.138961	O(51)-Lp(107)	0.5972	0.6000	H(85)-C(53)-O(52)	110.5144	106.7000	C(29)-O(52)-C(53)-H(85)	62.0239	C(30)-Lp(108)	2.2607
C(4)	C Alkene	0	-0.037348	O(51)-Lp(106)	0.5987	0.6000	H(84)-C(53)-H(83)	109.1725	109.0000	Lp(108)-O(52)-C(53)-H(83)	69.434	C(26)-Lp(100)	2.2802
C(5)	C Alkene	0	-0.0522545	N(39)-Lp(105)	0.6017	0.6000	H(84)-C(53)-O(52)	109.9346	106.7000	Lp(108)-O(52)-C(53)-H(84)	-171.486	H(22)-H(73)	2.2664
C(6)	C Alkene	0	0.163587	O(36)-Lp(104)	0.6003	0.6000	H(83)-C(53)-O(52)	108.0951	106.7000	Lp(108)-O(52)-C(53)-H(85)	-49.8172	C(24)-Lp(98)	2.2799
C(7)	C Alkane	0	0.0927996	O(36)-Lp(103)	0.5977	0.6000	Lp(109)-O(52)-Lp(108)	126.8037	131.0000	Lp(109)-O(52)-C(53)-H(83)	-67.6846	O(31)-O(52)	2.6397
N(8)	N Amine	0	0.418904	O(33)-Lp(102)	0.6005	0.6000	Lp(109)-O(52)-C(53)	105.5050	105.3600	Lp(109)-O(52)-C(53)-H(84)	51.3954	O(32)-C(51)	2.7037
C(9)	C Alkane	0	0.168202	O(33)-Lp(101)	0.5999	0.6000	Lp(109)-O(52)-C(29)	101.8466	103.2600	Lp(109)-O(52)-C(53)-H(85)	173.0641	C(25)-O(32)	2.7163
C(10)	C Alkane	0	-0.0732595	O(32)-Lp(100)	0.5990	0.6000	Lp(108)-O(52)-C(53)	106.7526	105.3600	C(2)-O(49)-C(50)-H(77)	-179.33	O(17)-C(45)	2.7276
C(11)	C Alkane	0	0.11456	O(32)-Lp(99)	0.5994	0.6000	Lp(108)-O(52)-C(29)	101.8916	103.2600	C(2)-O(49)-C(50)-H(78)	-60.169	H(42)-O(31)	2.4710
C(12)	C Alkane	0	0.164665	O(31)-Lp(98)	0.5978	0.6000	C(53)-O(52)-C(29)	114.3011	110.8000	C(2)-O(49)-C(50)-H(79)	61.426	C(14)-O(31)	2.7185
N(13)	N Amine	0	0.457761	O(31)-Lp(97)	0.5992	0.6000	H(82)-C(51)-H(81)	106.0029	109.0000	Lp(106)-O(49)-C(50)-H(77)	68.1522	C(24)-C(28)	2.6947
C(14)	C Alkane	0	0.151903	O(20)-Lp(96)	0.5977	0.6000	H(82)-C(51)-H(80)	106.2949	109.0000	Lp(106)-O(49)-C(50)-H(78)	-172.6868	H(42)-H(67)	2.0445
O(15)	O Carboxyl	0	-0.121838	O(20)-Lp(95)	0.5956	0.6000	H(82)-C(51)-C(28)	114.6502	110.0000	Lp(106)-O(49)-C(50)-H(79)	-51.0918	C(26)-C(29)	2.7080

Atom	Atom Type (MM2)	Charge (MM2)	Charge (Huckel)	Bond lengths			Bond Angles			Dihedral angles		Close contacts	
				Atoms	Actual (° / Å)	Optimal (° / Å)	Atoms	Actual (° / Å)	Optimal (° / Å)	Atoms	Actual (° / Å)	Atoms	Actual (° / Å)
C(16)	C Alkane	0	0.203196	O(17)-Lp(94)	0.5995	0.6000	H(81)-C(51)-H(80)	108.7651	109.0000	Lp(107)-O(49)-C(50)-H(77)	-67.03	C(30)-C(27)	2.7384
O(17)	O Carboxyl	0	-0.129563	O(17)-Lp(93)	0.5983	0.6000	H(81)-C(51)-C(28)	110.4214	110.0000	Lp(107)-O(49)-C(50)-H(78)	52.131	O(20)-C(21)	2.7767
C(18)	C Carbonyl	0	0.548583	O(15)-Lp(92)	0.5941	0.6000	H(80)-C(51)-C(28)	110.4344	110.0000	Lp(107)-O(49)-C(50)-H(79)	173.726	C(16)-O(20)	2.6500
C(19)	C Alkene	0	-0.0102227	O(15)-Lp(91)	0.5973	0.6000	Lp(100)-O(32)-Lp(99)	123.7418	131.0000	C(35)-C(41)-C(40)-N(39)	-0.1503	C(54)-H(42)	2.5408
O(20)	O Carbonyl	0	-0.663833	N(13)-Lp(90)	0.5996	0.6000	Lp(100)-O(32)-C(27)	118.0222	120.0000	C(35)-C(41)-C(40)-H(66)	179.8452	C(47)-H(43)	2.5382
C(21)	C Alkene	0	0.044475	N(8)-Lp(89)	0.6002	0.6000	Lp(99)-O(32)-C(27)	118.2357	120.0000	H(67)-C(41)-C(40)-N(39)	-179.9451	C(25)-H(58)	2.5108
H(22)	H	0	0.0216876	C(54)-H(88)	1.1127	1.1110	H(76)-C(46)-H(75)	106.3649	109.0000	H(67)-C(41)-C(40)-H(66)	0.0504	C(37)-C(40)	2.5989
H(23)	H	0	0.0180569	C(54)-H(87)	1.1130	1.1110	H(76)-C(46)-H(74)	110.4109	109.0000	C(41)-C(40)-N(39)-C(38)	0.0975	C(35)-N(39)	2.7143
C(24)	C Alkene	0	-0.0439434	C(54)-H(86)	1.1128	1.1110	H(76)-C(46)-C(21)	111.4032	110.0000	C(41)-C(40)-N(39)-Lp(105)	-179.9026	C(35)-Lp(103)	2.2817
C(25)	C Alkane	0	-0.0240229	O(53)-C(54)	1.4104	1.3960	H(75)-C(46)-H(74)	106.6518	109.0000	H(66)-C(40)-N(39)-C(38)	-179.8981	C(38)-C(41)	2.5909
C(26)	C Alkene	0	-0.11643	C(52)-H(85)	1.1123	1.1110	H(75)-C(46)-C(21)	110.6109	110.0000	H(66)-C(40)-N(39)-Lp(105)	0.1017	N(13)-C(26)	2.8086
C(27)	C Carbonyl	0	0.174341	C(52)-H(84)	1.1129	1.1110	H(74)-C(46)-C(21)	111.1847	110.0000	C(40)-N(39)-C(38)-C(37)	0.0045	N(8)-N(13)	2.6081
C(28)	C Alkene	0	-0.12622	C(52)-H(83)	1.1127	1.1110	H(73)-C(45)-H(72)	106.5662	109.0000	C(40)-N(39)-C(38)-H(65)	-179.9846	C(24)-C(12)	2.8103
C(29)	C Alkene	0	0.130624	O(51)-C(52)	1.4102	1.3960	H(73)-C(45)-H(71)	106.5799	109.0000	Lp(105)-N(39)-C(38)-C(37)	-179.9954	H(55)-C(47)	2.5943
C(30)	C Carbonyl	0	0.13768	C(50)-H(82)	1.1118	1.1130	H(73)-C(45)-C(19)	113.3953	110.0000	Lp(105)-N(39)-C(38)-H(65)	0.0155	O(36)-C(37)	2.6995
O(31)	O Carbonyl	0	-0.924738	C(50)-H(81)	1.1139	1.1130	H(72)-C(45)-H(71)	109.0033	109.0000	C(35)-C(37)-C(38)-N(39)	-0.0516	Lp(91)-O(36)	2.1315
O(32)	O Carbonyl	0	-0.923221	C(50)-H(80)	1.1148	1.1130	H(72)-C(45)-C(19)	110.5941	110.0000	C(35)-C(37)-C(38)-H(65)	179.9371	H(69)-O(36)	2.4295
O(33)	O Enol	0	-0.309786	C(49)-H(79)	1.1104	1.1130	H(71)-C(45)-C(19)	110.4970	110.0000	H(64)-C(37)-C(38)-N(39)	-179.9503	H(23)-C(24)	2.4963
C(34)	C Carbonyl	0	0.607333	C(49)-H(78)	1.1140	1.1130	Lp(98)-O(31)-Lp(97)	123.4265	131.0000	H(64)-C(37)-C(38)-H(65)	0.0384	O(15)-H(57)	2.3913
C(35)	C Alkene	0	0.0342873	C(49)-H(77)	1.1140	1.1130	Lp(98)-O(31)-C(30)	118.1628	120.0000	C(34)-C(35)-C(41)-C(40)	-179.8635	C(11)-H(55)	2.4376
O(36)	O Carbonyl	0	-0.643808	C(48)-H(76)	1.1124	1.1130	Lp(97)-O(31)-C(30)	118.4012	120.0000	C(34)-C(35)-C(41)-H(67)	-0.0772	O(33)-H(55)	2.4415
C(37)	C Alkene	0	-0.037284	C(48)-H(75)	1.1138	1.1130	C(51)-C(28)-C(29)	121.3685	121.4000	C(37)-C(35)-C(41)-C(40)	0.0933	C(11)-C(14)	2.7434
C(38)	C Alkene	0	0.124564	C(48)-H(74)	1.1140	1.1130	C(51)-C(28)-C(27)	117.2622	120.0000	C(37)-C(35)-C(41)-H(67)	179.8797	C(44)-C(34)	2.8209
N(39)	N Pyridine	0	-0.182561	C(46)-N(47)	1.1585	1.1580	C(29)-C(28)-C(27)	121.3694	117.6000	C(38)-C(37)-C(35)-C(34)	179.9551	C(4)-C(9)	2.7295
C(40)	C Alkene	0	0.125022	C(45)-H(73)	1.1133	1.1130	O(52)-C(29)-C(30)	117.8559	120.0000	C(38)-C(37)-C(35)-C(41)	-0.0012	Lp(107)-H(68)	1.8690
C(41)	C Alkene	0	-0.0395701	C(45)-H(72)	1.1141	1.1130	O(52)-C(29)-C(28)	121.8246	124.3000	H(64)-C(37)-C(35)-C(34)	-0.1514	O(49)-H(68)	2.4053
H(42)	H	0	0.0236254	C(45)-H(71)	1.1135	1.1130	C(30)-C(29)-C(28)	119.9209	117.6000	H(64)-C(37)-C(35)-C(41)	179.8923	C(7)-C(10)	2.7981
H(43)	H	0	0.0155556	C(44)-H(70)	1.1123	1.1130	O(32)-C(27)-C(28)	121.1945	123.0000	O(15)-C(34)-O(36)-Lp(103)	179.3177	C(3)-Lp(106)	2.2535
C(44)	C Alkane	0	-0.141806	C(44)-H(69)	1.1114	1.1130	O(32)-C(27)-C(26)	120.5132	123.0000	O(15)-C(34)-O(36)-Lp(104)	-1.5393	C(2)-C(5)	2.6687
C(45)	C Alkane	0	-0.129619	C(44)-H(68)	1.1094	1.1130	C(28)-C(27)-C(26)	118.2913	115.0000	C(35)-C(34)-O(36)-Lp(103)	1.4988	C(3)-C(6)	2.7225

Atom	Atom Type (MM2)	Charge (MM2)	Charge (Huckel)	Bond lengths			Bond Angles			Dihedral angles		Close contacts	
				Atoms	Actual (° / Å)	Optimal (° / Å)	Atoms	Actual (° / Å)	Optimal (° / Å)	Atoms	Actual (° / Å)	Atoms	Actual (° / Å)
C(46)	C Alkane	0	-0.135871	C(41)-H(67)	1.0968	1.1000	C(46)-C(21)-H(22)	113.6026	118.2000	C(35)-C(34)-O(36)-Lp(104)	-179.3582	C(1)-C(4)	2.7037
C(47)	C Alkyne	0	0.36012	C(40)-H(66)	1.1037	1.1000	C(46)-C(21)-C(19)	129.0800	122.0000	O(15)-C(34)-C(35)-C(37)	-179.679		
N(48)	N Nitrile	0	-0.441919	C(41)-C(40)	1.3425	1.4200	H(22)-C(21)-C(19)	117.3173	120.0000	O(15)-C(34)-C(35)-C(41)	0.2749		
O(49)	O Enol	0	-0.283353	C(40)-N(39)	1.2648	1.3580	Lp(96)-O(20)-Lp(95)	123.5611	131.0000	O(36)-C(34)-C(35)-C(37)	-1.9662		
C(50)	C Alkane	0	0.0896958	C(38)-H(65)	1.1036	1.1000	Lp(96)-O(20)-C(18)	117.3617	120.0000	O(36)-C(34)-C(35)-C(41)	177.9878		
C(51)	C Alkane	0	-0.136662	N(39)-C(38)	1.2652	1.3580	Lp(95)-O(20)-C(18)	119.0760	120.0000	C(24)-C(30)-O(31)-Lp(97)	-178.7315		
O(52)	O Enol	0	-0.277353	C(37)-H(64)	1.1019	1.1000	H(88)-C(54)-H(87)	108.5548	109.0000	C(24)-C(30)-O(31)-Lp(98)	0.19		
C(53)	C Alkane	0	0.0879094	C(37)-C(38)	1.3437	1.4200	H(88)-C(54)-H(86)	107.2818	109.0000	C(29)-C(30)-O(31)-Lp(97)	0.7099		
C(54)	C Alkane	0	-0.0284995	C(35)-C(41)	1.3449	1.4200	H(88)-C(54)-N(13)	113.3756		C(29)-C(30)-O(31)-Lp(98)	179.6314		
H(55)	H	0	0.0381096	C(37)-C(35)	1.3473	1.4200	H(87)-C(54)-H(86)	106.4505	109.0000	C(28)-C(29)-O(52)-C(53)	99.8236		
H(56)	H	0	0.038633	C(34)-O(36)	1.2190	1.2080	H(87)-C(54)-N(13)	110.6841		C(28)-C(29)-O(52)-Lp(108)	-145.4502		
H(57)	H	0	0.0268922	C(34)-C(35)	1.3691	1.5170	H(86)-C(54)-N(13)	110.2031		C(28)-C(29)-O(52)-Lp(109)	-13.4056		
H(58)	H	0	0.0479723	O(33)-H(63)	0.9693	0.9720	H(66)-C(40)-C(41)	119.9229	120.0000	C(30)-C(29)-O(52)-C(53)	-87.4331		
H(59)	H	0	0.0138719	C(30)-O(31)	1.2165	1.2080	H(66)-C(40)-N(39)	116.2227	116.5000	C(30)-C(29)-O(52)-Lp(108)	27.2931		
H(60)	H	0	0.01352	C(29)-O(51)	1.3763	1.3550	C(41)-C(40)-N(39)	123.8543	123.5000	C(30)-C(29)-O(52)-Lp(109)	159.3377		
H(61)	H	0	0.0335808	C(29)-C(30)	1.3646	1.5170	Lp(105)-N(39)-C(40)	121.3108	122.5000	C(28)-C(29)-C(30)-C(24)	-2.4505		
H(62)	H	0	0.0393661	C(28)-C(49)	1.5184	1.4970	Lp(105)-N(39)-C(38)	121.4950	122.5000	C(28)-C(29)-C(30)-O(31)	178.1001		
H(63)	H Enol	0	0.210837	C(28)-C(29)	1.3488	1.3370	C(40)-N(39)-C(38)	117.1942	115.0000	O(52)-C(29)-C(30)-C(24)	-175.3373		
H(64)	H	0	0.0187534	C(27)-O(32)	1.2157	1.2080	O(31)-C(30)-C(29)	120.8784	123.0000	O(52)-C(29)-C(30)-O(31)	5.2133		
H(65)	H	0	-0.00433347	C(27)-C(28)	1.3606	1.5170	O(31)-C(30)-C(24)	120.2926	123.0000	C(27)-C(28)-C(51)-H(80)	-60.321		
H(66)	H	0	-0.00460296	C(26)-C(27)	1.3601	1.5170	C(29)-C(30)-C(24)	118.8267	115.0000	C(27)-C(28)-C(51)-H(81)	60.0126		
H(67)	H	0	0.023311	C(25)-H(62)	1.1156	1.1130	H(62)-C(25)-H(61)	106.3367	109.4000	C(27)-C(28)-C(51)-H(82)	179.6512		
H(68)	H	0	0.0419097	C(25)-H(61)	1.1152	1.1130	H(62)-C(25)-C(26)	108.4254	109.4100	C(29)-C(28)-C(51)-H(80)	119.7177		
H(69)	H	0	0.0488858	C(26)-C(25)	1.5185	1.4970	H(62)-C(25)-C(12)	110.1046	109.4100	C(29)-C(28)-C(51)-H(81)	-119.9487		
H(70)	H	0	0.0527083	C(30)-C(24)	1.3619	1.5170	H(61)-C(25)-C(26)	111.0137	109.4100	C(29)-C(28)-C(51)-H(82)	-0.3101		
H(71)	H	0	0.0493752	C(24)-C(26)	1.3458	1.3370	H(61)-C(25)-C(12)	108.4988	109.4100	C(27)-C(28)-C(29)-C(30)	2.6962		
H(72)	H	0	0.0492713	C(21)-C(45)	1.5069	1.4970	C(26)-C(25)-C(12)	112.2937	109.5000	C(27)-C(28)-C(29)-O(52)	175.2929		
H(73)	H	0	0.036735	C(21)-H(22)	1.1049	1.1000	C(45)-C(19)-C(21)	118.8202	121.4000	C(51)-C(28)-C(29)-C(30)	-177.344		
H(74)	H	0	0.0589382	C(19)-C(48)	1.5176	1.4970	C(45)-C(19)-C(18)	117.0340	120.0000	C(51)-C(28)-C(29)-O(52)	-4.7473		
H(75)	H	0	0.0425854	C(19)-C(21)	1.3483	1.3370	C(21)-C(19)-C(18)	124.1456	117.6000	C(26)-C(27)-O(32)-Lp(99)	-179.8722		

Atom	Atom Type (MM2)	Charge (MM2)	Charge (Huckel)	Bond lengths			Bond Angles			Dihedral angles		Close contacts	
				Atoms	Actual (° / Å)	Optimal (° / Å)	Atoms	Actual (° / Å)	Optimal (° / Å)	Atoms	Actual (° / Å)	Atoms	Actual (° / Å)
H(76)	H	0	0.0576163	C(18)-O(20)	1.2141	1.2080	H(67)-C(41)-C(40)	118.2165	120.0000	C(26)-C(27)-O(32)-Lp(100)	0.3249		
H(77)	H	0	0.0283359	C(18)-C(19)	1.3668	1.5170	H(67)-C(41)-C(35)	122.1635	120.0000	C(28)-C(27)-O(32)-Lp(99)	-0.2405		
H(78)	H	0	0.0229532	O(17)-C(18)	1.3765	1.3380	C(40)-C(41)-C(35)	119.6197		C(28)-C(27)-O(32)-Lp(100)	179.9566		
H(79)	H	0	0.0229268	C(16)-H(60)	1.1146	1.1110	H(65)-C(38)-N(39)	115.8685	116.5000	C(26)-C(27)-C(28)-C(29)	-1.6251		
H(80)	H	0	0.0458539	C(16)-H(59)	1.1146	1.1110	H(65)-C(38)-C(37)	119.9121	120.0000	C(26)-C(27)-C(28)-C(51)	178.4136		
H(81)	H	0	0.0463508	C(16)-O(17)	1.4030	1.3890	N(39)-C(38)-C(37)	124.2194	123.5000	O(32)-C(27)-C(28)-C(29)	178.7353		
H(82)	H	0	0.0380449	O(15)-C(34)	1.3755	1.3380	C(27)-C(26)-C(25)	118.4647	120.0000	O(32)-C(27)-C(28)-C(51)	-1.2261		
H(83)	H	0	0.0284207	C(14)-H(42)	1.1122	1.1130	C(27)-C(26)-C(24)	120.8140	117.6000	C(24)-C(26)-C(27)-C(28)	0.3617		
H(84)	H	0	0.0230242	C(14)-C(24)	1.5272	1.4970	C(25)-C(26)-C(24)	120.7189	121.4000	C(24)-C(26)-C(27)-O(32)	-179.9961		
H(85)	H	0	0.0233932	N(13)-C(50)	1.4536	1.4380	O(20)-C(18)-C(19)	122.4555	123.0000	C(25)-C(26)-C(27)-C(28)	179.8171		
H(86)	H	0	0.0381677	N(13)-C(14)	1.4521	1.4380	O(20)-C(18)-O(17)	118.5593	122.0000	C(25)-C(26)-C(27)-O(32)	-0.5407		
H(87)	H	0	0.0345729	C(12)-H(43)	1.1185	1.1130	C(19)-C(18)-O(17)	118.9821	124.3000	C(24)-C(26)-C(25)-C(12)	-9.7656		
H(88)	H	0	0.0443887	C(12)-C(25)	1.5375	1.5230	Lp(90)-N(13)-C(54)	106.5115	109.2000	C(24)-C(26)-C(25)-H(61)	-131.4235		
				C(12)-N(13)	1.4506	1.4380	Lp(90)-N(13)-C(14)	107.0368	109.2000	C(24)-C(26)-C(25)-H(62)	112.1163		
				C(11)-H(58)	1.1189	1.1130	Lp(90)-N(13)-C(12)	106.7567	109.2000	C(27)-C(26)-C(25)-C(12)	170.7784		
				C(11)-C(46)	1.4804	1.4700	C(54)-N(13)-C(14)	113.4248	107.7000	C(27)-C(26)-C(25)-H(61)	49.1206		
				C(11)-C(12)	1.5511	1.5230	C(54)-N(13)-C(12)	114.7038	107.7000	C(27)-C(26)-C(25)-H(62)	-67.3397		
				C(10)-H(57)	1.1128	1.1130	C(14)-N(13)-C(12)	107.9309	107.7000	C(29)-C(30)-C(24)-C(14)	-178.9026		
				C(10)-H(56)	1.1146	1.1130	H(43)-C(12)-C(25)	109.3471	109.3900	C(29)-C(30)-C(24)-C(26)	1.2241		
				C(9)-H(23)	1.1210	1.1130	H(43)-C(12)-N(13)	108.4808	108.8000	O(31)-C(30)-C(24)-C(14)	0.5501		
				C(14)-C(9)	1.5526	1.5230	H(43)-C(12)-C(11)	108.1542	109.3900	O(31)-C(30)-C(24)-C(26)	-179.3231		
				C(9)-C(10)	1.5371	1.5230	C(25)-C(12)-N(13)	110.1992	108.8000	C(14)-C(24)-C(26)-C(25)	0.4965		
				N(8)-C(11)	1.4617	1.4380	C(25)-C(12)-C(11)	110.7442	109.5100	C(14)-C(24)-C(26)-C(27)	179.9396		
				N(8)-C(9)	1.4537	1.4380	N(13)-C(12)-C(11)	109.8578	108.8000	C(30)-C(24)-C(26)-C(25)	-179.6333		
				C(7)-H(55)	1.1158	1.1130	N(48)-C(47)-C(11)	178.1155	180.0000	C(30)-C(24)-C(26)-C(27)	-0.1902		
				C(7)-C(16)	1.5403	1.5140	H(64)-C(37)-C(38)	117.9898	120.0000	C(19)-C(21)-C(46)-H(74)	63.8802		
				C(7)-N(8)	1.4683	1.4380	H(64)-C(37)-C(35)	122.8753	120.0000	C(19)-C(21)-C(46)-H(75)	-177.8189		
				C(6)-O(15)	1.3787	1.3550	C(38)-C(37)-C(35)	119.1348		C(19)-C(21)-C(46)-H(76)	-59.7371		
				C(10)-C(5)	1.5104	1.4970	Lp(104)-O(36)-Lp(103)	123.7894	131.0000	H(22)-C(21)-C(46)-H(74)	-116.1076		
				C(5)-C(6)	1.3527	1.4200	Lp(104)-O(36)-C(34)	117.5487	120.0000	H(22)-C(21)-C(46)-H(75)	2.1933		

Atom	Atom Type (MM2)	Charge (MM2)	Charge (Huckel)	Bond lengths			Bond Angles			Dihedral angles		Close contacts	
				Atoms	Actual (° / Å)	Optimal (° / Å)	Atoms	Actual (° / Å)	Optimal (° / Å)	Atoms	Actual (° / Å)	Atoms	Actual (° / Å)
				C(4)-C(7)	1.5333	1.4970	Lp(103)-O(36)-C(34)	118.6559	120.0000	H(22)-C(21)-C(46)-H(76)	120.2751		
				C(4)-C(5)	1.3433	1.4200	C(30)-C(24)-C(26)	120.7267	117.6000	C(18)-C(19)-C(45)-H(71)	-60.9331		
				C(3)-O(33)	1.3636	1.3550	C(30)-C(24)-C(14)	118.4333	120.0000	C(18)-C(19)-C(45)-H(72)	59.8528		
				C(3)-C(4)	1.3451	1.4200	C(26)-C(24)-C(14)	120.8398	121.4000	C(18)-C(19)-C(45)-H(73)	179.4855		
				C(2)-O(53)	1.3760	1.3550	Lp(94)-O(17)-Lp(93)	127.2983	131.0000	C(21)-C(19)-C(45)-H(71)	118.9232		
				C(2)-C(3)	1.3481	1.4200	Lp(94)-O(17)-C(18)	101.4449	105.1600	C(21)-C(19)-C(45)-H(72)	-120.2909		
				C(1)-C(44)	1.5200	1.4970	Lp(94)-O(17)-C(16)	105.0324	105.3600	C(21)-C(19)-C(45)-H(73)	-0.6582		
				C(6)-C(1)	1.3555	1.4200	Lp(93)-O(17)-C(18)	100.4284	105.1600	C(18)-C(19)-C(21)-H(22)	179.6167		
				C(1)-C(2)	1.3542	1.4200	Lp(93)-O(17)-C(16)	104.7021	105.3600	C(18)-C(19)-C(21)-C(46)	-0.3707		
							C(18)-O(17)-C(16)	119.3773	109.9000	C(45)-C(19)-C(21)-H(22)	-0.2287		
							C(41)-C(35)-C(37)	115.9775	120.0000	C(45)-C(19)-C(21)-C(46)	179.7839		
							C(41)-C(35)-C(34)	121.3891	117.6000	O(17)-C(18)-O(20)-Lp(95)	179.4551		
							C(37)-C(35)-C(34)	122.6334	117.6000	O(17)-C(18)-O(20)-Lp(96)	-0.1607		
							H(60)-C(16)-H(59)	109.3496	109.4000	C(19)-C(18)-O(20)-Lp(95)	0.1086		
							H(60)-C(16)-O(17)	109.0893	106.7000	C(19)-C(18)-O(20)-Lp(96)	-179.5073		
							H(60)-C(16)-C(7)	109.7169	109.4100	O(17)-C(18)-C(19)-C(21)	179.7813		
							H(59)-C(16)-O(17)	108.7833	106.7000	O(17)-C(18)-C(19)-C(45)	-0.3708		
							H(59)-C(16)-C(7)	110.1011	109.4100	O(20)-C(18)-C(19)-C(21)	-0.8748		
							O(17)-C(16)-C(7)	109.7780	107.4000	O(20)-C(18)-C(19)-C(45)	178.9731		
							H(42)-C(14)-C(24)	108.9196	109.3900	C(16)-O(17)-C(18)-C(19)	178.0293		
							H(42)-C(14)-N(13)	105.7623	108.8000	C(16)-O(17)-C(18)-O(20)	-1.3404		
							H(42)-C(14)-C(9)	108.8444	109.3900	Lp(93)-O(17)-C(18)-C(19)	64.467		
							C(24)-C(14)-N(13)	111.5714		Lp(93)-O(17)-C(18)-O(20)	-114.9027		
							C(24)-C(14)-C(9)	111.7397	109.5100	Lp(94)-O(17)-C(18)-C(19)	-67.2846		
							N(13)-C(14)-C(9)	109.7896	108.8000	Lp(94)-O(17)-C(18)-O(20)	113.3457		
							H(58)-C(11)-C(47)	102.9669	109.3900	C(7)-C(16)-O(17)-C(18)	-179.1467		
							H(58)-C(11)-C(12)	109.4016	109.3900	C(7)-C(16)-O(17)-Lp(93)	-67.8954		
							H(58)-C(11)-N(8)	107.0304	108.8000	C(7)-C(16)-O(17)-Lp(94)	68.088		
							C(47)-C(11)-C(12)	113.6814	112.4000	H(59)-C(16)-O(17)-C(18)	60.3184		
							C(47)-C(11)-N(8)	110.8003		H(59)-C(16)-O(17)-Lp(93)	171.5696		

Atom	Atom Type (MM2)	Charge (MM2)	Charge (Huckel)	Bond lengths			Bond Angles			Dihedral angles		Close contacts	
				Atoms	Actual (° / Å)	Optimal (° / Å)	Atoms	Actual (° / Å)	Optimal (° / Å)	Atoms	Actual (° / Å)	Atoms	Actual (° / Å)
							C(12)-C(11)-N(8)	112.3287	108.8000	H(59)-C(16)-O(17)-Lp(94)	-52.4469		
							O(36)-C(34)-C(35)	119.6266	123.0000	H(60)-C(16)-O(17)-C(18)	-58.8931		
							O(36)-C(34)-O(15)	117.8316	122.0000	H(60)-C(16)-O(17)-Lp(93)	52.3581		
							C(35)-C(34)-O(15)	122.5040	124.3000	H(60)-C(16)-O(17)-Lp(94)	-171.6584		
							H(23)-C(9)-C(14)	108.0865	109.3900	C(6)-O(15)-C(34)-C(35)	-73.941		
							H(23)-C(9)-C(10)	105.7636	109.3900	C(6)-O(15)-C(34)-O(36)	108.3072		
							H(23)-C(9)-N(8)	107.3092	108.8000	Lp(91)-O(15)-C(34)-C(35)	171.5894		
							C(14)-C(9)-C(10)	114.2759	109.5100	Lp(91)-O(15)-C(34)-O(36)	-6.1624		
							C(14)-C(9)-N(8)	111.9680	108.8000	Lp(92)-O(15)-C(34)-C(35)	38.1815		
							C(10)-C(9)-N(8)	109.0236	108.8000	Lp(92)-O(15)-C(34)-O(36)	-139.5703		
							Lp(89)-N(8)-C(11)	105.6448	109.2000	C(9)-C(14)-C(24)-C(26)	98.7861		
							Lp(89)-N(8)-C(9)	106.9324	109.2000	C(9)-C(14)-C(24)-C(30)	-81.087		
							Lp(89)-N(8)-C(7)	106.4109	109.2000	N(13)-C(14)-C(24)-C(26)	-24.5527		
							C(11)-N(8)-C(9)	108.2697	107.7000	N(13)-C(14)-C(24)-C(30)	155.5741		
							C(11)-N(8)-C(7)	114.0919	107.7000	H(42)-C(14)-C(24)-C(26)	-140.9313		
							C(9)-N(8)-C(7)	114.8360	107.7000	H(42)-C(14)-C(24)-C(30)	39.1956		
							Lp(92)-O(15)-Lp(91)	129.2738	131.0000	C(12)-N(13)-C(54)-H(86)	170.6067		
							Lp(92)-O(15)-C(34)	98.4347	105.1600	C(12)-N(13)-C(54)-H(87)	53.1241		
							Lp(92)-O(15)-C(6)	103.4755	103.2600	C(12)-N(13)-C(54)-H(88)	-69.1344		
							Lp(91)-O(15)-C(34)	102.2757	105.1600	C(14)-N(13)-C(54)-H(86)	-64.7385		
							Lp(91)-O(15)-C(6)	102.9592	103.2600	C(14)-N(13)-C(54)-H(87)	177.7789		
							C(34)-O(15)-C(6)	122.9118	112.0000	C(14)-N(13)-C(54)-H(88)	55.5205		
							H(57)-C(10)-H(56)	106.8490	109.4000	Lp(90)-N(13)-C(54)-H(86)	52.7431		
							H(57)-C(10)-C(9)	107.8930	109.4100	Lp(90)-N(13)-C(54)-H(87)	-64.7394		
							H(57)-C(10)-C(5)	113.5442	109.4100	Lp(90)-N(13)-C(54)-H(88)	173.0021		
							H(56)-C(10)-C(9)	111.5287	109.4100	C(12)-N(13)-C(14)-C(9)	-66.4203		
							H(56)-C(10)-C(5)	109.5317	109.4100	C(12)-N(13)-C(14)-C(24)	58.0193		
							C(9)-C(10)-C(5)	107.5430	109.5000	C(12)-N(13)-C(14)-H(42)	176.3056		
							H(55)-C(7)-C(16)	107.6119	109.3900	C(54)-N(13)-C(14)-C(9)	165.3429		
							H(55)-C(7)-N(8)	108.5449	108.8000	C(54)-N(13)-C(14)-C(24)	-70.2174		

Atom	Atom Type (MM2)	Charge (MM2)	Charge (Huckel)	Bond lengths			Bond Angles			Dihedral angles		Close contacts	
				Atoms	Actual (° / Å)	Optimal (° / Å)	Atoms	Actual (° / Å)	Optimal (° / Å)	Atoms	Actual (° / Å)	Atoms	Actual (° / Å)
							H(55)-C(7)-C(4)	108.3537	109.3900	C(54)-N(13)-C(14)-H(42)	48.0689		
							C(16)-C(7)-N(8)	107.9726	108.8000	Lp(90)-N(13)-C(14)-C(9)	48.1677		
							C(16)-C(7)-C(4)	109.0499	109.5100	Lp(90)-N(13)-C(14)-C(24)	172.6074		
							N(8)-C(7)-C(4)	115.0709		Lp(90)-N(13)-C(14)-H(42)	-69.1063		
							H(79)-C(50)-H(78)	110.0859	109.0000	C(11)-C(12)-C(25)-C(26)	-78.2481		
							H(79)-C(50)-H(77)	109.1173	109.0000	C(11)-C(12)-C(25)-H(61)	44.8348		
							H(79)-C(50)-O(49)	110.2486	106.7000	C(11)-C(12)-C(25)-H(62)	160.8314		
							H(78)-C(50)-H(77)	109.1627	109.0000	N(13)-C(12)-C(25)-C(26)	43.5134		
							H(78)-C(50)-O(49)	110.0106	106.7000	N(13)-C(12)-C(25)-H(61)	166.5963		
							H(77)-C(50)-O(49)	108.1769	106.7000	N(13)-C(12)-C(25)-H(62)	-77.407		
							H(70)-C(44)-H(69)	111.1933	109.0000	H(43)-C(12)-C(25)-C(26)	162.6672		
							H(70)-C(44)-H(68)	105.4055	109.0000	H(43)-C(12)-C(25)-H(61)	-74.2499		
							H(70)-C(44)-C(1)	111.1351	110.0000	H(43)-C(12)-C(25)-H(62)	41.7468		
							H(69)-C(44)-H(68)	102.7009	109.0000	C(11)-C(12)-N(13)-C(14)	53.6538		
							H(69)-C(44)-C(1)	110.6619	110.0000	C(11)-C(12)-N(13)-C(54)	-178.844		
							H(68)-C(44)-C(1)	115.3769	110.0000	C(11)-C(12)-N(13)-Lp(90)	-61.1194		
							Lp(102)-O(33)-Lp(101)	113.3897		C(25)-C(12)-N(13)-C(14)	-68.6321		
							Lp(102)-O(33)-H(63)	103.5395	101.1000	C(25)-C(12)-N(13)-C(54)	58.8701		
							Lp(102)-O(33)-C(3)	111.9647		C(25)-C(12)-N(13)-Lp(90)	176.5947		
							Lp(101)-O(33)-H(63)	103.3889	101.1000	H(43)-C(12)-N(13)-C(14)	171.6877		
							Lp(101)-O(33)-C(3)	113.5660		H(43)-C(12)-N(13)-C(54)	-60.8101		
							H(63)-O(33)-C(3)	110.1014	108.0000	H(43)-C(12)-N(13)-Lp(90)	56.9144		
							C(10)-C(5)-C(6)	121.3008	121.4000	N(8)-C(11)-C(47)-N(48)	76.2967		
							C(10)-C(5)-C(4)	115.7968	121.4000	C(12)-C(11)-C(47)-N(48)	-51.3204		
							C(6)-C(5)-C(4)	122.8873	120.0000	H(58)-C(11)-C(47)-N(48)	-169.5634		
							Lp(107)-O(49)-Lp(106)	126.2385	131.0000	N(8)-C(11)-C(12)-N(13)	9.0828		
							Lp(107)-O(49)-C(50)	105.0307	105.3600	N(8)-C(11)-C(12)-C(25)	131.0446		
							Lp(107)-O(49)-C(2)	103.4342	103.2600	N(8)-C(11)-C(12)-H(43)	-109.1533		
							Lp(106)-O(49)-C(50)	105.4621	105.3600	C(47)-C(11)-C(12)-N(13)	135.9046		
							Lp(106)-O(49)-C(2)	103.4056	103.2600	C(47)-C(11)-C(12)-C(25)	-102.1336		

Atom	Atom Type (MM2)	Charge (MM2)	Charge (Huckel)	Bond lengths			Bond Angles			Dihedral angles		Close contacts	
				Atoms	Actual (° / Å)	Optimal (° / Å)	Atoms	Actual (° / Å)	Optimal (° / Å)	Atoms	Actual (° / Å)	Atoms	Actual (° / Å)
							C(50)-O(49)-C(2)	113.5506	110.8000	C(47)-C(11)-C(12)-H(43)	17.6685		
							O(15)-C(6)-C(5)	116.4474	124.3000	H(58)-C(11)-C(12)-N(13)	-109.6242		
							O(15)-C(6)-C(1)	126.2836	124.3000	H(58)-C(11)-C(12)-C(25)	12.3376		
							C(5)-C(6)-C(1)	117.2381	120.0000	H(58)-C(11)-C(12)-H(43)	132.1397		
							C(7)-C(4)-C(5)	121.1592	121.4000	N(13)-C(14)-C(9)-N(8)	13.6166		
							C(7)-C(4)-C(3)	120.2558	121.4000	N(13)-C(14)-C(9)-C(10)	-110.9594		
							C(5)-C(4)-C(3)	118.5718	120.0000	N(13)-C(14)-C(9)-H(23)	131.6013		
							O(33)-C(3)-C(4)	120.6080	124.3000	C(24)-C(14)-C(9)-N(8)	-110.7255		
							O(33)-C(3)-C(2)	119.0365	124.3000	C(24)-C(14)-C(9)-C(10)	124.6986		
							C(4)-C(3)-C(2)	120.3364	120.0000	C(24)-C(14)-C(9)-H(23)	7.2593		
							O(49)-C(2)-C(3)	117.8435	124.3000	H(42)-C(14)-C(9)-N(8)	128.9478		
							O(49)-C(2)-C(1)	121.8185	124.3000	H(42)-C(14)-C(9)-C(10)	4.3719		
							C(3)-C(2)-C(1)	119.9088	120.0000	H(42)-C(14)-C(9)-H(23)	-113.0674		
							C(44)-C(1)-C(6)	121.3702	121.4000	N(8)-C(9)-C(10)-C(5)	-67.6031		
							C(44)-C(1)-C(2)	117.8885	121.4000	N(8)-C(9)-C(10)-H(56)	52.5054		
							C(6)-C(1)-C(2)	120.7408	120.0000	N(8)-C(9)-C(10)-H(57)	169.5567		
										C(14)-C(9)-C(10)-C(5)	58.5245		
										C(14)-C(9)-C(10)-H(56)	178.6331		
										C(14)-C(9)-C(10)-H(57)	-64.3156		
										H(23)-C(9)-C(10)-C(5)	177.2877		
										H(23)-C(9)-C(10)-H(56)	-62.6038		
										H(23)-C(9)-C(10)-H(57)	54.4475		
										C(7)-N(8)-C(11)-C(12)	68.602		
										C(7)-N(8)-C(11)-C(47)	-59.7506		
										C(7)-N(8)-C(11)-H(58)	-171.3059		
										C(9)-N(8)-C(11)-C(12)	-60.6065		
										C(9)-N(8)-C(11)-C(47)	171.0409		
										C(9)-N(8)-C(11)-H(58)	59.4856		
										Lp(89)-N(8)-C(11)-C(12)	-174.8714		
										Lp(89)-N(8)-C(11)-C(47)	56.776		

Atom	Atom Type (MM2)	Charge (MM2)	Charge (Huckel)	Bond lengths			Bond Angles			Dihedral angles		Close contacts	
				Atoms	Actual (° / Å)	Optimal (° / Å)	Atoms	Actual (° / Å)	Optimal (° / Å)	Atoms	Actual (° / Å)	Atoms	Actual (° / Å)
										Lp(89)-N(8)-C(11)-H(58)	-54.7793		
										C(7)-N(8)-C(9)-C(10)	46.4097		
										C(7)-N(8)-C(9)-C(14)	-81.036		
										C(7)-N(8)-C(9)-H(23)	160.5169		
										C(11)-N(8)-C(9)-C(10)	175.2001		
										C(11)-N(8)-C(9)-C(14)	47.7544		
										C(11)-N(8)-C(9)-H(23)	-70.6927		
										Lp(89)-N(8)-C(9)-C(10)	-71.3865		
										Lp(89)-N(8)-C(9)-C(14)	161.1678		
										Lp(89)-N(8)-C(9)-H(23)	42.7207		
										C(4)-C(7)-C(16)-O(17)	58.5583		
										C(4)-C(7)-C(16)-H(59)	178.2919		
										C(4)-C(7)-C(16)-H(60)	-61.3134		
										N(8)-C(7)-C(16)-O(17)	-175.7589		
										N(8)-C(7)-C(16)-H(59)	-56.0252		
										N(8)-C(7)-C(16)-H(60)	64.3695		
										H(55)-C(7)-C(16)-O(17)	-58.7723		
										H(55)-C(7)-C(16)-H(59)	60.9614		
										H(55)-C(7)-C(16)-H(60)	-178.6439		
										C(4)-C(7)-N(8)-C(9)	-0.5118		
										C(4)-C(7)-N(8)-C(11)	-126.3398		
										C(4)-C(7)-N(8)-Lp(89)	117.5788		
										C(16)-C(7)-N(8)-C(9)	-122.5564		
										C(16)-C(7)-N(8)-C(11)	111.6156		
										C(16)-C(7)-N(8)-Lp(89)	-4.4658		
										H(55)-C(7)-N(8)-C(9)	121.063		
										H(55)-C(7)-N(8)-C(11)	-4.7651		
										H(55)-C(7)-N(8)-Lp(89)	-120.8464		
										C(1)-C(6)-O(15)-C(34)	-25.5397		
										C(1)-C(6)-O(15)-Lp(91)	88.5909		

Atom	Atom Type (MM2)	Charge (MM2)	Charge (Huckel)	Bond lengths			Bond Angles			Dihedral angles		Close contacts	
				Atoms	Actual (° / Å)	Optimal (° / Å)	Atoms	Actual (° / Å)	Optimal (° / Å)	Atoms	Actual (° / Å)	Atoms	Actual (° / Å)
										C(1)-C(6)-O(15)-Lp(92)	-135.0978		
										C(5)-C(6)-O(15)-C(34)	156.5472		
										C(5)-C(6)-O(15)-Lp(91)	-89.3222		
										C(5)-C(6)-O(15)-Lp(92)	46.9891		
										C(9)-C(10)-C(5)-C(4)	40.268		
										C(9)-C(10)-C(5)-C(6)	-138.3588		
										H(56)-C(10)-C(5)-C(4)	-81.0983		
										H(56)-C(10)-C(5)-C(6)	100.2749		
										H(57)-C(10)-C(5)-C(4)	159.5582		
										H(57)-C(10)-C(5)-C(6)	-19.0687		
										C(4)-C(5)-C(6)-C(1)	-0.629		
										C(4)-C(5)-C(6)-O(15)	177.4791		
										C(10)-C(5)-C(6)-C(1)	177.8988		
										C(10)-C(5)-C(6)-O(15)	-3.9932		
										C(3)-C(4)-C(7)-N(8)	152.5327		
										C(3)-C(4)-C(7)-C(16)	-86.0055		
										C(3)-C(4)-C(7)-H(55)	30.8544		
										C(5)-C(4)-C(7)-N(8)	-28.8081		
										C(5)-C(4)-C(7)-C(16)	92.6537		
										C(5)-C(4)-C(7)-H(55)	-150.4864		
										C(3)-C(4)-C(5)-C(6)	3.5057		
										C(3)-C(4)-C(5)-C(10)	-175.097		
										C(7)-C(4)-C(5)-C(6)	-175.1755		
										C(7)-C(4)-C(5)-C(10)	6.2217		
										C(2)-C(3)-O(33)-H(63)	-161.3684		
										C(2)-C(3)-O(33)-Lp(101)	-45.9867		
										C(2)-C(3)-O(33)-Lp(102)	84.0231		
										C(4)-C(3)-O(33)-H(63)	17.044		
										C(4)-C(3)-O(33)-Lp(101)	132.4257		
										C(4)-C(3)-O(33)-Lp(102)	-97.5645		

Atom	Atom Type (MM2)	Charge (MM2)	Charge (Huckel)	Bond lengths			Bond Angles			Dihedral angles		Close contacts	
				Atoms	Actual (° / Å)	Optimal (° / Å)	Atoms	Actual (° / Å)	Optimal (° / Å)	Atoms	Actual (° / Å)	Atoms	Actual (° / Å)
										C(2)-C(3)-C(4)-C(5)	-1.3698		
										C(2)-C(3)-C(4)-C(7)	177.3237		
										O(33)-C(3)-C(4)-C(5)	-179.7615		
										O(33)-C(3)-C(4)-C(7)	-1.068		
										C(1)-C(2)-O(49)-C(50)	96.5443		
										C(1)-C(2)-O(49)-Lp(106)	-149.6991		
										C(1)-C(2)-O(49)-Lp(107)	-16.7199		
										C(3)-C(2)-O(49)-C(50)	-90.985		
										C(3)-C(2)-O(49)-Lp(106)	22.7716		
										C(3)-C(2)-O(49)-Lp(107)	155.7508		
										C(1)-C(2)-C(3)-C(4)	-3.5292		
										C(1)-C(2)-C(3)-O(33)	174.8877		
										O(49)-C(2)-C(3)-C(4)	-176.1492		
										O(49)-C(2)-C(3)-O(33)	2.2676		
										C(2)-C(1)-C(44)-H(68)	13.833		
										C(2)-C(1)-C(44)-H(69)	129.8697		
										C(2)-C(1)-C(44)-H(70)	-106.0737		
										C(6)-C(1)-C(44)-H(68)	-165.9045		
										C(6)-C(1)-C(44)-H(69)	-49.8678		
										C(6)-C(1)-C(44)-H(70)	74.1888		
										C(5)-C(6)-C(1)-C(2)	-4.3976		
										C(5)-C(6)-C(1)-C(44)	175.3325		
										O(15)-C(6)-C(1)-C(2)	177.7039		
										O(15)-C(6)-C(1)-C(44)	-2.566		
										C(6)-C(1)-C(2)-C(3)	6.513		
										C(6)-C(1)-C(2)-O(49)	178.8317		
										C(44)-C(1)-C(2)-C(3)	-173.2262		
										C(44)-C(1)-C(2)-O(49)	-0.9076		

Table S2. Theoretical level of minimization of the 3D structures of **11**

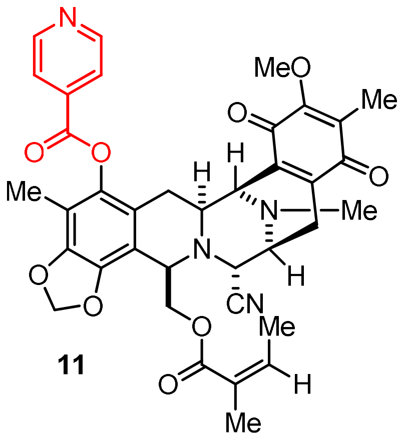

11

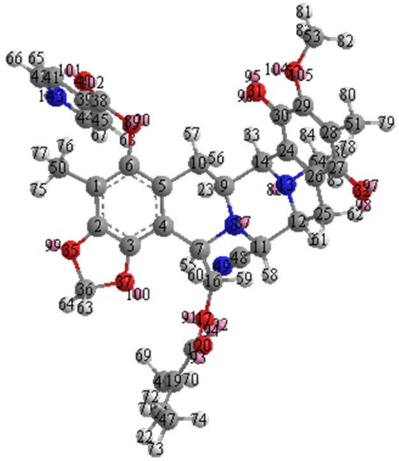

Atom	Atom Type (MM2)	Charge (MM2)	Charge (Huckel)	Bond lengths			Bond Angles			Dihedral angles		Close contacts	
				Atoms	Actual (° / Å)	Optimal (° / Å)	Atoms	Actual (° / Å)	Optimal (° / Å)	Atoms	Actual (° / Å)	Atoms	Actual (° / Å)
C(1)	C Alkene	0	-0.141775	O(52)-Lp(105)	0.5971	0.6000	H(83)-C(53)-H(82)	110.0482	109.0000	C(29)-O(52)-C(53)-H(81)	-178.6821	Lp(105)-H(80)	1.9599
C(2)	C Alkene	0	0.165611	O(52)-Lp(104)	0.5987	0.6000	H(83)-C(53)-H(81)	109.0447	109.0000	C(29)-O(52)-C(53)-H(82)	-59.5872	C(28)-Lp(105)	2.2855
C(3)	C Alkene	0	0.154807	N(43)-Lp(103)	0.6017	0.6000	H(83)-C(53)-O(52)	110.4806	106.7000	C(29)-O(52)-C(53)-H(83)	62.0800	C(30)-Lp(104)	2.2619
C(4)	C Alkene	0	-0.0953676	O(40)-Lp(102)	0.6002	0.6000	H(82)-C(53)-H(81)	109.1751	109.0000	Lp(104)-O(52)-C(53)-H(81)	69.2809	C(26)-Lp(98)	2.2829
C(5)	C Alkene	0	-0.102308	O(40)-Lp(101)	0.5975	0.6000	H(82)-C(53)-O(52)	109.9609	106.7000	Lp(104)-O(52)-C(53)-H(82)	-171.6242	H(22)-H(71)	2.2694
C(6)	C Alkene	0	0.145271	O(37)-Lp(100)	0.6002	0.6000	H(81)-C(53)-O(52)	108.0895	106.7000	Lp(104)-O(52)-C(53)-H(83)	-49.9570	C(24)-Lp(96)	2.2863
C(7)	C Alkane	0	0.0701674	O(35)-Lp(99)	0.6021	0.6000	Lp(105)-O(52)-Lp(104)	126.6872	131.0000	Lp(105)-O(52)-C(53)-H(81)	-67.6968	C(29)-Lp(95)	2.2840
N(8)	N Amine	0	0.347871	O(32)-Lp(98)	0.5991	0.6000	Lp(105)-O(52)-C(53)	105.4498	105.3600	Lp(105)-O(52)-C(53)-H(82)	51.3981	O(31)-O(52)	2.6312
C(9)	C Alkane	0	0.126878	O(32)-Lp(97)	0.5993	0.6000	Lp(105)-O(52)-C(29)	101.8062	103.2600	Lp(105)-O(52)-C(53)-H(83)	173.0653	O(32)-C(51)	2.7030
C(10)	C Alkane	0	-0.0810699	O(31)-Lp(96)	0.5973	0.6000	Lp(104)-O(52)-C(53)	106.8189	105.3600	C(39)-C(45)-C(44)-N(43)	-0.0016	C(25)-O(32)	2.7096
C(11)	C Alkane	0	0.111517	O(31)-Lp(95)	0.5991	0.6000	Lp(104)-O(52)-C(29)	101.9971	103.2600	C(39)-C(45)-C(44)-H(67)	179.9507	O(17)-C(46)	2.7244
C(12)	C Alkane	0	0.15499	O(20)-Lp(94)	0.5981	0.6000	C(53)-O(52)-C(29)	114.3562	110.8000	H(68)-C(45)-C(44)-N(43)	-179.7173	H(33)-O(31)	2.4572
N(13)	N Amine	0	0.450068	O(20)-Lp(93)	0.5956	0.6000	H(80)-C(51)-H(79)	106.0292	109.0000	H(68)-C(45)-C(44)-H(67)	0.2350	C(14)-O(31)	2.7711
C(14)	C Alkane	0	0.130466	O(17)-Lp(92)	0.5993	0.6000	H(80)-C(51)-H(78)	106.2458	109.0000	C(45)-C(44)-N(43)-C(42)	0.1380	C(24)-C(28)	2.6999
O(15)	O Carboxyl	0	-0.135328	O(17)-Lp(91)	0.5958	0.6000	H(80)-C(51)-C(28)	114.6760	110.0000	C(45)-C(44)-N(43)-Lp(103)	-179.9445	C(6)-H(68)	2.5919

Atom	Atom Type (MM2)	Charge (MM2)	Charge (Huckel)	Bond lengths			Bond Angles			Dihedral angles		Close contacts	
				Atoms	Actual (° / Å)	Optimal (° / Å)	Atoms	Actual (° / Å)	Optimal (° / Å)	Atoms	Actual (° / Å)	Atoms	Actual (° / Å)
C(16)	C Alkane	0	0.156664	O(15)-Lp(90)	0.5933	0.6000	H(79)-C(51)-H(78)	108.7737	109.0000	H(67)-C(44)-N(43)-C(42)	-179.8160	C(26)-C(29)	2.7036
O(17)	O Carboxyl	0	-0.147843	O(15)-Lp(89)	0.5973	0.6000	H(79)-C(51)-C(28)	110.4247	110.0000	H(67)-C(44)-N(43)-Lp(103)	0.1015	C(30)-C(27)	2.7360
C(18)	C Carbonyl	0	0.551039	N(13)-Lp(88)	0.6014	0.6000	H(78)-C(51)-C(28)	110.4192	110.0000	C(44)-N(43)-C(42)-C(41)	-0.0927	O(20)-C(21)	2.7780
C(19)	C Alkene	0	-0.0109144	N(8)-Lp(87)	0.6007	0.6000	Lp(98)-O(32)-Lp(97)	123.6652	131.0000	C(44)-N(43)-C(42)-H(66)	179.9612	H(60)-O(20)	2.4376
O(20)	O Carbonyl	0	-0.66274	C(54)-H(86)	1.1118	1.1130	Lp(98)-O(32)-C(27)	118.0839	120.0000	Lp(103)-N(43)-C(42)-C(41)	179.9898	C(16)-O(20)	2.6838
C(21)	C Alkene	0	0.045386	C(54)-H(85)	1.1141	1.1130	Lp(97)-O(32)-C(27)	118.2507	120.0000	Lp(103)-N(43)-C(42)-H(66)	0.0438	O(40)-H(65)	2.4759
H(22)	H	0	0.0217088	C(54)-H(84)	1.1141	1.1130	H(74)-C(47)-H(73)	106.2398	109.0000	C(39)-C(41)-C(42)-N(43)	-0.0876	C(41)-C(44)	2.5956
H(23)	H	0	0.0406023	C(53)-H(83)	1.1122	1.1110	H(74)-C(47)-H(72)	110.4003	109.0000	C(39)-C(41)-C(42)-H(66)	179.8563	C(39)-N(43)	2.7151
C(24)	C Alkene	0	-0.0367738	C(53)-H(82)	1.1129	1.1110	H(74)-C(47)-C(21)	111.5457	110.0000	H(65)-C(41)-C(42)-N(43)	-179.8807	C(39)-Lp(101)	2.2848
C(25)	C Alkane	0	-0.0240526	C(53)-H(81)	1.1127	1.1110	H(73)-C(47)-H(72)	106.8006	109.0000	H(65)-C(41)-C(42)-H(66)	0.0633	C(18)-H(60)	2.5498
C(26)	C Alkene	0	-0.0925668	O(52)-C(53)	1.4100	1.3960	H(73)-C(47)-C(21)	110.5940	110.0000	C(38)-C(39)-C(45)-C(44)	179.9042	C(4)-H(60)	2.5202
C(27)	C Carbonyl	0	0.181202	C(51)-H(80)	1.1104	1.1130	H(72)-C(47)-C(21)	111.0473	110.0000	C(38)-C(39)-C(45)-H(68)	-0.3960	N(8)-H(59)	2.4892
C(28)	C Alkene	0	-0.126983	C(51)-H(79)	1.1140	1.1130	H(71)-C(46)-H(70)	106.6175	109.0000	C(41)-C(39)-C(45)-C(44)	-0.1742	C(42)-C(45)	2.5955
C(29)	C Alkene	0	0.132764	C(51)-H(78)	1.1140	1.1130	H(71)-C(46)-H(69)	106.6803	109.0000	C(41)-C(39)-C(45)-H(68)	179.5255	N(13)-C(26)	2.8156
C(30)	C Carbonyl	0	0.13943	C(50)-H(77)	1.1129	1.1130	H(71)-C(46)-C(19)	113.3386	110.0000	C(42)-C(41)-C(39)-C(38)	-179.8638	C(24)-C(12)	2.8631
O(31)	O Carbonyl	0	-0.925735	C(50)-H(76)	1.1122	1.1130	H(70)-C(46)-H(69)	108.7513	109.0000	C(42)-C(41)-C(39)-C(45)	0.2148	C(9)-C(12)	2.8435
O(32)	O Carbonyl	0	-0.910527	C(50)-H(75)	1.1119	1.1130	H(70)-C(46)-C(19)	110.6851	110.0000	H(65)-C(41)-C(39)-C(38)	-0.0820	H(55)-C(48)	2.4005
H(33)	H	0	0.0177218	C(48)-N(49)	1.1586	1.1580	H(69)-C(46)-C(19)	110.5509	110.0000	H(65)-C(41)-C(39)-C(45)	179.9966	O(40)-C(41)	2.6925
H(34)	H	0	0.0149847	C(47)-H(74)	1.1132	1.1130	Lp(96)-O(31)-Lp(95)	123.4984	131.0000	O(15)-C(38)-O(40)-Lp(101)	179.6392	Lp(89)-O(40)	2.1339
O(35)	O Furan	0	-0.171518	C(47)-H(73)	1.1141	1.1130	Lp(96)-O(31)-C(30)	118.1325	120.0000	O(15)-C(38)-O(40)-Lp(102)	-1.2077	H(55)-O(17)	2.4037
C(36)	C Alkane	0	0.400965	C(47)-H(72)	1.1135	1.1130	Lp(95)-O(31)-C(30)	118.3662	120.0000	C(39)-C(38)-O(40)-Lp(101)	1.1667	Lp(90)-H(57)	1.9268
O(37)	O Furan	0	-0.161504	C(46)-H(71)	1.1124	1.1130	C(51)-C(28)-C(29)	121.4437	121.4000	C(39)-C(38)-O(40)-Lp(102)	-179.6802	O(15)-H(57)	2.4317
C(38)	C Carbonyl	0	0.608853	C(46)-H(70)	1.1140	1.1130	C(51)-C(28)-C(27)	117.3218	120.0000	O(15)-C(38)-C(39)-C(41)	179.5660	C(11)-H(55)	2.5306
C(39)	C Alkene	0	0.0330278	C(46)-H(69)	1.1141	1.1130	C(29)-C(28)-C(27)	121.2338	117.6000	O(15)-C(38)-C(39)-C(45)	-0.5173	C(11)-C(14)	2.8255
O(40)	O Carbonyl	0	-0.641866	C(45)-H(68)	1.0999	1.1000	O(52)-C(29)-C(30)	117.9438	120.0000	O(40)-C(38)-C(39)-C(41)	-2.0422	C(50)-C(38)	2.8722
C(41)	C Alkene	0	-0.0354614	C(44)-H(67)	1.1037	1.1000	O(52)-C(29)-C(28)	121.6500	124.3000	O(40)-C(38)-C(39)-C(45)	177.8745	C(4)-C(9)	2.7952
C(42)	C Alkene	0	0.124994	C(45)-C(44)	1.3432	1.4200	C(30)-C(29)-C(28)	119.9514	117.6000	O(35)-C(36)-O(37)-C(3)	0.1169	C(2)-C(5)	2.6401
N(43)	N Pyridine	0	-0.178624	C(44)-N(43)	1.2648	1.3580	O(32)-C(27)-C(28)	121.1152	123.0000	O(35)-C(36)-O(37)-Lp(100)	166.7924	C(3)-C(6)	2.6857
C(44)	C Alkene	0	0.125458	C(42)-H(66)	1.1036	1.1000	O(32)-C(27)-C(26)	120.6364	123.0000	H(63)-C(36)-O(37)-C(3)	-118.0686	C(1)-C(4)	2.7515
C(45)	C Alkene	0	-0.0385508	N(43)-C(42)	1.2648	1.3580	C(28)-C(27)-C(26)	118.2476	115.0000	H(63)-C(36)-O(37)-Lp(100)	48.6069		

Atom	Atom Type (MM2)	Charge (MM2)	Charge (Huckel)	Bond lengths			Bond Angles			Dihedral angles		Close contacts	
				Atoms	Actual (° / Å)	Optimal (° / Å)	Atoms	Actual (° / Å)	Optimal (° / Å)	Atoms	Actual (° / Å)	Atoms	Actual (° / Å)
C(46)	C Alkane	0	-0.129009	C(41)-H(65)	1.1020	1.1000	C(47)-C(21)-H(22)	113.6394	118.2000	H(64)-C(36)-O(37)-C(3)	118.5426		
C(47)	C Alkane	0	-0.135835	C(41)-C(42)	1.3434	1.4200	C(47)-C(21)-C(19)	129.0427	122.0000	H(64)-C(36)-O(37)-Lp(100)	-74.7820		
C(48)	C Alkyne	0	0.371016	C(39)-C(45)	1.3466	1.4200	H(22)-C(21)-C(19)	117.3173	120.0000	C(2)-O(35)-C(36)-O(37)	0.0602		
N(49)	N Nitrile	0	-0.421866	C(41)-C(39)	1.3477	1.4200	Lp(94)-O(20)-Lp(93)	123.4273	131.0000	C(2)-O(35)-C(36)-H(63)	118.2115		
C(50)	C Alkane	0	-0.140105	C(38)-O(40)	1.2192	1.2080	Lp(94)-O(20)-C(18)	117.4215	120.0000	C(2)-O(35)-C(36)-H(64)	-118.0947		
C(51)	C Alkane	0	-0.134824	C(38)-C(39)	1.3695	1.5170	Lp(93)-O(20)-C(18)	119.1509	120.0000	Lp(99)-O(35)-C(36)-O(37)	158.3840		
O(52)	O Enol	0	-0.272413	C(36)-H(64)	1.1146	1.1130	H(86)-C(54)-H(85)	107.8125	109.0000	Lp(99)-O(35)-C(36)-H(63)	-83.4647		
C(53)	C Alkane	0	0.0878443	C(36)-H(63)	1.1147	1.1130	H(86)-C(54)-H(84)	107.7237	109.0000	Lp(99)-O(35)-C(36)-H(64)	40.2290		
C(54)	C Alkane	0	-0.03178	C(36)-O(37)	1.4240	1.4140	H(86)-C(54)-N(13)	113.3826		C(24)-C(30)-O(31)-Lp(95)	-179.2556		
H(55)	H	0	0.0499938	O(35)-C(36)	1.4261	1.4140	H(85)-C(54)-H(84)	106.5166	109.0000	C(24)-C(30)-O(31)-Lp(96)	0.1446		
H(56)	H	0	0.0413223	C(30)-O(31)	1.2162	1.2080	H(85)-C(54)-N(13)	110.5095		C(29)-C(30)-O(31)-Lp(95)	0.5425		
H(57)	H	0	0.0308042	C(29)-O(52)	1.3765	1.3550	H(84)-C(54)-N(13)	110.6061		C(29)-C(30)-O(31)-Lp(96)	179.9427		
H(58)	H	0	0.0430694	C(29)-C(30)	1.3651	1.5170	H(67)-C(44)-C(45)	119.9545	120.0000	C(28)-C(29)-O(52)-C(53)	99.4168		
H(59)	H	0	0.0173619	C(28)-C(51)	1.5184	1.4970	H(67)-C(44)-N(43)	115.9556	116.5000	C(28)-C(29)-O(52)-Lp(104)	-145.6913		
H(60)	H	0	0.0174401	C(28)-C(29)	1.3484	1.3370	C(45)-C(44)-N(43)	124.0898	123.5000	C(28)-C(29)-O(52)-Lp(105)	-13.7472		
H(61)	H	0	0.0366083	C(27)-O(32)	1.2158	1.2080	Lp(103)-N(43)-C(44)	121.3735	122.5000	C(30)-C(29)-O(52)-C(53)	-88.3296		
H(62)	H	0	0.0407877	C(27)-C(28)	1.3600	1.5170	Lp(103)-N(43)-C(42)	121.4465	122.5000	C(30)-C(29)-O(52)-Lp(104)	26.5623		
H(63)	H	0	0.00574024	C(26)-C(27)	1.3600	1.5170	C(44)-N(43)-C(42)	117.1799	115.0000	C(30)-C(29)-O(52)-Lp(105)	158.5064		
H(64)	H	0	0.00576146	C(25)-H(62)	1.1176	1.1130	O(31)-C(30)-C(29)	120.2758	123.0000	C(28)-C(29)-C(30)-C(24)	-1.8217		
H(65)	H	0	0.0187835	C(25)-H(61)	1.1149	1.1130	O(31)-C(30)-C(24)	120.6973	123.0000	C(28)-C(29)-C(30)-O(31)	178.3769		
H(66)	H	0	-0.00404144	C(26)-C(25)	1.5150	1.4970	C(29)-C(30)-C(24)	119.0266	115.0000	O(52)-C(29)-C(30)-C(24)	-174.2118		
H(67)	H	0	-0.00450932	C(30)-C(24)	1.3627	1.5170	H(62)-C(25)-H(61)	106.9471	109.4000	O(52)-C(29)-C(30)-O(31)	5.9868		
H(68)	H	0	0.0227564	C(24)-C(26)	1.3444	1.3370	H(62)-C(25)-C(26)	107.0768	109.4100	C(27)-C(28)-C(51)-H(78)	-60.0973		
H(69)	H	0	0.0496646	C(21)-C(47)	1.5068	1.4970	H(62)-C(25)-C(12)	107.8789	109.4100	C(27)-C(28)-C(51)-H(79)	60.2392		
H(70)	H	0	0.0483295	C(21)-H(22)	1.1048	1.1000	H(61)-C(25)-C(26)	109.9175	109.4100	C(27)-C(28)-C(51)-H(80)	179.9322		
H(71)	H	0	0.0367351	C(19)-C(46)	1.5181	1.4970	H(61)-C(25)-C(12)	109.5691	109.4100	C(29)-C(28)-C(51)-H(78)	120.2102		
H(72)	H	0	0.0596888	C(19)-C(21)	1.3481	1.3370	C(26)-C(25)-C(12)	115.0973	109.5000	C(29)-C(28)-C(51)-H(79)	-119.4532		
H(73)	H	0	0.0426911	C(18)-O(20)	1.2140	1.2080	C(46)-C(19)-C(21)	118.9044	121.4000	C(29)-C(28)-C(51)-H(80)	0.2397		
H(74)	H	0	0.0568657	C(18)-C(19)	1.3669	1.5170	C(46)-C(19)-C(18)	116.9057	120.0000	C(27)-C(28)-C(29)-C(30)	3.1959		
H(75)	H	0	0.0399295	O(17)-C(18)	1.3754	1.3380	C(21)-C(19)-C(18)	124.1897	117.6000	C(27)-C(28)-C(29)-O(52)	175.2969		

Atom	Atom Type (MM2)	Charge (MM2)	Charge (Huckel)	Bond lengths			Bond Angles			Dihedral angles		Close contacts	
				Atoms	Actual (° / Å)	Optimal (° / Å)	Atoms	Actual (° / Å)	Optimal (° / Å)	Atoms	Actual (° / Å)	Atoms	Actual (° / Å)
H(76)	H	0	0.0455506	C(16)-H(60)	1.1122	1.1110	H(68)-C(45)-C(44)	117.6517	120.0000	C(51)-C(28)-C(29)-C(30)	-177.1237		
H(77)	H	0	0.0494254	C(16)-H(59)	1.1136	1.1110	H(68)-C(45)-C(39)	122.9969	120.0000	C(51)-C(28)-C(29)-O(52)	-5.0227		
H(78)	H	0	0.0456607	C(16)-O(17)	1.4062	1.3890	C(44)-C(45)-C(39)	119.3508		C(26)-C(27)-O(32)-Lp(97)	-179.9372		
H(79)	H	0	0.0462187	O(15)-C(38)	1.3766	1.3380	H(66)-C(42)-N(43)	115.9194	116.5000	C(26)-C(27)-O(32)-Lp(98)	0.2213		
H(80)	H	0	0.0381496	C(14)-H(33)	1.1177	1.1130	H(66)-C(42)-C(41)	119.9881	120.0000	C(28)-C(27)-O(32)-Lp(97)	-0.2820		
H(81)	H	0	0.0284281	C(14)-C(24)	1.5269	1.4970	N(43)-C(42)-C(41)	124.0925	123.5000	C(28)-C(27)-O(32)-Lp(98)	179.8764		
H(82)	H	0	0.0231376	N(13)-C(54)	1.4548	1.4380	C(27)-C(26)-C(25)	118.1263	120.0000	C(26)-C(27)-C(28)-C(29)	-2.1550		
H(83)	H	0	0.0234917	N(13)-C(14)	1.4557	1.4380	C(27)-C(26)-C(24)	121.1648	117.6000	C(26)-C(27)-C(28)-C(51)	178.1519		
H(84)	H	0	0.0361682	C(12)-H(34)	1.1205	1.1130	C(25)-C(26)-C(24)	120.6916	121.4000	O(32)-C(27)-C(28)-C(29)	178.1819		
H(85)	H	0	0.0361927	C(12)-C(25)	1.5376	1.5230	O(20)-C(18)-C(19)	122.4660	123.0000	O(32)-C(27)-C(28)-C(51)	-1.5113		
H(86)	H	0	0.047826	C(12)-N(13)	1.4501	1.4380	O(20)-C(18)-O(17)	118.4926	122.0000	C(24)-C(26)-C(27)-C(28)	-0.2312		
				C(11)-H(58)	1.1170	1.1130	C(19)-C(18)-O(17)	118.9749	124.3000	C(24)-C(26)-C(27)-O(32)	179.4336		
				C(11)-C(48)	1.4835	1.4700	Lp(88)-N(13)-C(54)	107.5539	109.2000	C(25)-C(26)-C(27)-C(28)	178.2683		
				C(11)-C(12)	1.5409	1.5230	Lp(88)-N(13)-C(14)	107.3438	109.2000	C(25)-C(26)-C(27)-O(32)	-2.0669		
				C(10)-H(57)	1.1131	1.1130	Lp(88)-N(13)-C(12)	106.9561	109.2000	C(24)-C(26)-C(25)-C(12)	-0.1865		
				C(10)-H(56)	1.1147	1.1130	C(54)-N(13)-C(14)	113.0580	107.7000	C(24)-C(26)-C(25)-H(61)	-124.4574		
				C(9)-H(23)	1.1194	1.1130	C(54)-N(13)-C(12)	113.2758	107.7000	C(24)-C(26)-C(25)-H(62)	119.7164		
				C(14)-C(9)	1.5555	1.5230	C(14)-N(13)-C(12)	108.3142	107.7000	C(27)-C(26)-C(25)-C(12)	-178.6934		
				C(9)-C(10)	1.5362	1.5230	H(34)-C(12)-C(25)	108.5458	109.3900	C(27)-C(26)-C(25)-H(61)	57.0357		
				N(8)-C(11)	1.4664	1.4380	H(34)-C(12)-N(13)	109.2541	108.8000	C(27)-C(26)-C(25)-H(62)	-58.7904		
				N(8)-C(9)	1.4501	1.4380	H(34)-C(12)-C(11)	106.0936	109.3900	C(29)-C(30)-C(24)-C(14)	179.1484		
				C(7)-H(55)	1.1177	1.1130	C(25)-C(12)-N(13)	112.3896	108.8000	C(29)-C(30)-C(24)-C(26)	-0.5189		
				C(7)-C(16)	1.5433	1.5140	C(25)-C(12)-C(11)	112.1455	109.5100	O(31)-C(30)-C(24)-C(14)	-1.0511		
				C(7)-N(8)	1.4650	1.4380	N(13)-C(12)-C(11)	108.1942	108.8000	O(31)-C(30)-C(24)-C(26)	179.2816		
				C(6)-O(15)	1.3775	1.3550	N(49)-C(48)-C(11)	178.6194	180.0000	C(14)-C(24)-C(26)-C(25)	3.4199		
				C(10)-C(5)	1.5104	1.4970	H(65)-C(41)-C(42)	117.7427	120.0000	C(14)-C(24)-C(26)-C(27)	-178.1190		
				C(5)-C(6)	1.3601	1.4200	H(65)-C(41)-C(39)	122.9479	120.0000	C(30)-C(24)-C(26)-C(25)	-176.9087		
				C(4)-C(7)	1.5243	1.4970	C(42)-C(41)-C(39)	119.3091		C(30)-C(24)-C(26)-C(27)	1.5524		
				C(4)-C(5)	1.3466	1.4200	Lp(102)-O(40)-Lp(101)	123.8053	131.0000	C(19)-C(21)-C(47)-H(72)	65.8835		
				O(37)-C(3)	1.2283	1.4210	Lp(102)-O(40)-C(38)	117.4936	120.0000	C(19)-C(21)-C(47)-H(73)	-175.7284		

Atom	Atom Type (MM2)	Charge (MM2)	Charge (Huckel)	Bond lengths			Bond Angles			Dihedral angles		Close contacts	
				Atoms	Actual (° / Å)	Optimal (° / Å)	Atoms	Actual (° / Å)	Optimal (° / Å)	Atoms	Actual (° / Å)	Atoms	Actual (° / Å)
				C(3)-C(4)	1.3402	1.4200	Lp(101)-O(40)-C(38)	118.6952	120.0000	C(19)-C(21)-C(47)-H(74)	-57.7232		
				C(2)-O(35)	1.2297	1.4210	C(30)-C(24)-C(26)	120.3026	117.6000	H(22)-C(21)-C(47)-H(72)	-113.8276		
				C(2)-C(3)	1.3347	1.4200	C(30)-C(24)-C(14)	120.4708	120.0000	H(22)-C(21)-C(47)-H(73)	4.5606		
				C(1)-C(50)	1.5137	1.4970	C(26)-C(24)-C(14)	119.2258	121.4000	H(22)-C(21)-C(47)-H(74)	122.5658		
				C(6)-C(1)	1.3567	1.4200	Lp(92)-O(17)-Lp(91)	127.2767	131.0000	C(18)-C(19)-C(46)-H(69)	-62.7313		
				C(1)-C(2)	1.3418	1.4200	Lp(92)-O(17)-C(18)	100.2689	105.1600	C(18)-C(19)-C(46)-H(70)	57.8337		
							Lp(92)-O(17)-C(16)	105.5038	105.3600	C(18)-C(19)-C(46)-H(71)	177.5587		
							Lp(91)-O(17)-C(18)	100.9181	105.1600	C(21)-C(19)-C(46)-H(69)	117.1127		
							Lp(91)-O(17)-C(16)	104.6792	105.3600	C(21)-C(19)-C(46)-H(70)	-122.3223		
							C(18)-O(17)-C(16)	119.6452	109.9000	C(21)-C(19)-C(46)-H(71)	-2.5974		
							C(45)-C(39)-C(41)	115.9774	120.0000	C(18)-C(19)-C(21)-H(22)	179.2450		
							C(45)-C(39)-C(38)	121.9226	117.6000	C(18)-C(19)-C(21)-C(47)	-0.4570		
							C(41)-C(39)-C(38)	122.0999	117.6000	C(46)-C(19)-C(21)-H(22)	-0.5868		
							H(60)-C(16)-H(59)	106.1945	109.4000	C(46)-C(19)-C(21)-C(47)	179.7112		
							H(60)-C(16)-O(17)	111.3524	106.7000	O(17)-C(18)-O(20)-Lp(93)	178.2352		
							H(60)-C(16)-C(7)	109.4805	109.4100	O(17)-C(18)-O(20)-Lp(94)	-1.9763		
							H(59)-C(16)-O(17)	107.9909	106.7000	C(19)-C(18)-O(20)-Lp(93)	1.2346		
							H(59)-C(16)-C(7)	112.4596	109.4100	C(19)-C(18)-O(20)-Lp(94)	-178.9770		
							O(17)-C(16)-C(7)	109.3473	107.4000	O(17)-C(18)-C(19)-C(21)	-179.2567		
							H(33)-C(14)-C(24)	110.3189	109.3900	O(17)-C(18)-C(19)-C(46)	0.5782		
							H(33)-C(14)-N(13)	105.8861	108.8000	O(20)-C(18)-C(19)-C(21)	-2.2700		
							H(33)-C(14)-C(9)	107.1066	109.3900	O(20)-C(18)-C(19)-C(46)	177.5649		
							C(24)-C(14)-N(13)	110.9634		C(16)-O(17)-C(18)-C(19)	157.2021		
							C(24)-C(14)-C(9)	112.3930	109.5100	C(16)-O(17)-C(18)-O(20)	-19.9053		
							N(13)-C(14)-C(9)	109.8997	108.8000	Lp(91)-O(17)-C(18)-C(19)	43.2026		
							H(58)-C(11)-C(48)	105.1334	109.3900	Lp(91)-O(17)-C(18)-O(20)	-133.9049		
							H(58)-C(11)-C(12)	108.1793	109.3900	Lp(92)-O(17)-C(18)-C(19)	-88.2361		
							H(58)-C(11)-N(8)	109.7685	108.8000	Lp(92)-O(17)-C(18)-O(20)	94.6564		
							C(48)-C(11)-C(12)	110.5548	112.4000	C(7)-C(16)-O(17)-C(18)	-148.0337		
							C(48)-C(11)-N(8)	112.1376		C(7)-C(16)-O(17)-Lp(91)	-36.0483		

Atom	Atom Type (MM2)	Charge (MM2)	Charge (Huckel)	Bond lengths			Bond Angles			Dihedral angles		Close contacts	
				Atoms	Actual (° / Å)	Optimal (° / Å)	Atoms	Actual (° / Å)	Optimal (° / Å)	Atoms	Actual (° / Å)	Atoms	Actual (° / Å)
							C(12)-C(11)-N(8)	110.8419	108.8000	C(7)-C(16)-O(17)-Lp(92)	100.2054		
							O(40)-C(38)-C(39)	119.7654	123.0000	H(59)-C(16)-O(17)-C(18)	89.3005		
							O(40)-C(38)-O(15)	117.5659	122.0000	H(59)-C(16)-O(17)-Lp(91)	-158.7140		
							C(39)-C(38)-O(15)	122.6501	124.3000	H(59)-C(16)-O(17)-Lp(92)	-22.4603		
							H(23)-C(9)-C(14)	108.2334	109.3900	H(60)-C(16)-O(17)-C(18)	-26.9184		
							H(23)-C(9)-C(10)	107.9187	109.3900	H(60)-C(16)-O(17)-Lp(91)	85.0670		
							H(23)-C(9)-N(8)	109.0791	108.8000	H(60)-C(16)-O(17)-Lp(92)	-138.6793		
							C(14)-C(9)-C(10)	111.3273	109.5100	C(6)-O(15)-C(38)-C(39)	-66.6096		
							C(14)-C(9)-N(8)	111.3344	108.8000	C(6)-O(15)-C(38)-O(40)	114.9652		
							C(10)-C(9)-N(8)	108.8627	108.8000	Lp(89)-O(15)-C(38)-C(39)	-179.7986		
							Lp(87)-N(8)-C(11)	106.7900	109.2000	Lp(89)-O(15)-C(38)-O(40)	1.7762		
							Lp(87)-N(8)-C(9)	106.5303	109.2000	Lp(90)-O(15)-C(38)-C(39)	45.5699		
							Lp(87)-N(8)-C(7)	106.9021	109.2000	Lp(90)-O(15)-C(38)-O(40)	-132.8553		
							C(11)-N(8)-C(9)	110.3686	107.7000	C(9)-C(14)-C(24)-C(26)	88.4056		
							C(11)-N(8)-C(7)	111.4039	107.7000	C(9)-C(14)-C(24)-C(30)	-91.2652		
							C(9)-N(8)-C(7)	114.3809	107.7000	N(13)-C(14)-C(24)-C(26)	-35.1049		
							Lp(90)-O(15)-Lp(89)	129.9789	131.0000	N(13)-C(14)-C(24)-C(30)	145.2243		
							Lp(90)-O(15)-C(38)	98.7698	105.1600	H(33)-C(14)-C(24)-C(26)	-152.1343		
							Lp(90)-O(15)-C(6)	104.2265	103.2600	H(33)-C(14)-C(24)-C(30)	28.1949		
							Lp(89)-O(15)-C(38)	102.9499	105.1600	C(12)-N(13)-C(54)-H(84)	176.3329		
							Lp(89)-O(15)-C(6)	102.6460	103.2600	C(12)-N(13)-C(54)-H(85)	58.6273		
							C(38)-O(15)-C(6)	120.2017	112.0000	C(12)-N(13)-C(54)-H(86)	-62.5420		
							H(57)-C(10)-H(56)	108.7035	109.4000	C(14)-N(13)-C(54)-H(84)	-59.9649		
							H(57)-C(10)-C(9)	106.6970	109.4100	C(14)-N(13)-C(54)-H(85)	-177.6705		
							H(57)-C(10)-C(5)	111.5444	109.4100	C(14)-N(13)-C(54)-H(86)	61.1602		
							H(56)-C(10)-C(9)	111.0462	109.4100	Lp(88)-N(13)-C(54)-H(84)	58.3597		
							H(56)-C(10)-C(5)	105.9281	109.4100	Lp(88)-N(13)-C(54)-H(85)	-59.3460		
							C(9)-C(10)-C(5)	112.9126	109.5000	Lp(88)-N(13)-C(54)-H(86)	179.4848		
							H(55)-C(7)-C(16)	109.7920	109.3900	C(12)-N(13)-C(14)-C(9)	-61.7401		
							H(55)-C(7)-N(8)	108.5365	108.8000	C(12)-N(13)-C(14)-C(24)	63.1886		

Atom	Atom Type (MM2)	Charge (MM2)	Charge (Huckel)	Bond lengths			Bond Angles			Dihedral angles		Close contacts	
				Atoms	Actual (° / Å)	Optimal (° / Å)	Atoms	Actual (° / Å)	Optimal (° / Å)	Atoms	Actual (° / Å)	Atoms	Actual (° / Å)
							H(55)-C(7)-C(4)	101.4946	109.3900	C(12)-N(13)-C(14)-H(33)	-177.0980		
							C(16)-C(7)-N(8)	109.7789	108.8000	C(54)-N(13)-C(14)-C(9)	171.8702		
							C(16)-C(7)-C(4)	111.7862	109.5100	C(54)-N(13)-C(14)-C(24)	-63.2012		
							N(8)-C(7)-C(4)	115.0519		C(54)-N(13)-C(14)-H(33)	56.5122		
							H(77)-C(50)-H(76)	110.5864	109.0000	Lp(88)-N(13)-C(14)-C(9)	53.4234		
							H(77)-C(50)-H(75)	106.3973	109.0000	Lp(88)-N(13)-C(14)-C(24)	178.3521		
							H(77)-C(50)-C(1)	111.1264	110.0000	Lp(88)-N(13)-C(14)-H(33)	-61.9345		
							H(76)-C(50)-H(75)	104.4716	109.0000	C(11)-C(12)-C(25)-C(26)	-93.2006		
							H(76)-C(50)-C(1)	110.9266	110.0000	C(11)-C(12)-C(25)-H(61)	31.2531		
							H(75)-C(50)-C(1)	113.0625	110.0000	C(11)-C(12)-C(25)-H(62)	147.3395		
							Lp(100)-O(37)-C(36)	126.0944	124.2000	N(13)-C(12)-C(25)-C(26)	28.9571		
							Lp(100)-O(37)-C(3)	124.5807	122.2000	N(13)-C(12)-C(25)-H(61)	153.4109		
							C(36)-O(37)-C(3)	107.8714	113.6000	N(13)-C(12)-C(25)-H(62)	-90.5027		
							H(64)-C(36)-H(63)	110.7770	109.4000	H(34)-C(12)-C(25)-C(26)	149.9135		
							H(64)-C(36)-O(37)	110.7598		H(34)-C(12)-C(25)-H(61)	-85.6328		
							H(64)-C(36)-O(35)	111.1404		H(34)-C(12)-C(25)-H(62)	30.4536		
							H(63)-C(36)-O(37)	110.8504		C(11)-C(12)-N(13)-C(14)	63.9838		
							H(63)-C(36)-O(35)	110.8982		C(11)-C(12)-N(13)-C(54)	-169.7532		
							O(37)-C(36)-O(35)	102.1352		C(11)-C(12)-N(13)-Lp(88)	-51.4329		
							C(10)-C(5)-C(6)	120.0810	121.4000	C(25)-C(12)-N(13)-C(14)	-60.3871		
							C(10)-C(5)-C(4)	116.6915	121.4000	C(25)-C(12)-N(13)-C(54)	65.8759		
							C(6)-C(5)-C(4)	123.2274	120.0000	C(25)-C(12)-N(13)-Lp(88)	-175.8038		
							Lp(99)-O(35)-C(36)	124.8404	124.2000	H(34)-C(12)-N(13)-C(14)	179.0642		
							Lp(99)-O(35)-C(2)	123.3632	122.2000	H(34)-C(12)-N(13)-C(54)	-54.6728		
							C(36)-O(35)-C(2)	107.9187	113.6000	H(34)-C(12)-N(13)-Lp(88)	63.6475		
							O(15)-C(6)-C(5)	117.5280	124.3000	N(8)-C(11)-C(48)-N(49)	-16.4505		
							O(15)-C(6)-C(1)	123.6939	124.3000	C(12)-C(11)-C(48)-N(49)	-140.6982		
							C(5)-C(6)-C(1)	118.7574	120.0000	H(58)-C(11)-C(48)-N(49)	102.7770		
							C(7)-C(4)-C(5)	124.1118	121.4000	N(8)-C(11)-C(12)-N(13)	-61.6886		
							C(7)-C(4)-C(3)	119.6271	121.4000	N(8)-C(11)-C(12)-C(25)	62.8281		

Atom	Atom Type (MM2)	Charge (MM2)	Charge (Huckel)	Bond lengths			Bond Angles			Dihedral angles		Close contacts	
				Atoms	Actual (° / Å)	Optimal (° / Å)	Atoms	Actual (° / Å)	Optimal (° / Å)	Atoms	Actual (° / Å)	Atoms	Actual (° / Å)
							C(5)-C(4)-C(3)	116.2599	120.0000	N(8)-C(11)-C(12)-H(34)	-178.8242		
							O(37)-C(3)-C(4)	127.0254	120.0000	C(48)-C(11)-C(12)-N(13)	63.2981		
							O(37)-C(3)-C(2)	111.1905	120.0000	C(48)-C(11)-C(12)-C(25)	-172.1853		
							C(4)-C(3)-C(2)	121.7814	120.0000	C(48)-C(11)-C(12)-H(34)	-53.8375		
							O(35)-C(2)-C(3)	110.8835	120.0000	H(58)-C(11)-C(12)-N(13)	177.9183		
							O(35)-C(2)-C(1)	127.1152	120.0000	H(58)-C(11)-C(12)-C(25)	-57.5650		
							C(3)-C(2)-C(1)	121.9942	120.0000	H(58)-C(11)-C(12)-H(34)	60.7827		
							C(50)-C(1)-C(6)	123.8729	121.4000	N(13)-C(14)-C(9)-N(8)	56.6112		
							C(50)-C(1)-C(2)	118.1696	121.4000	N(13)-C(14)-C(9)-C(10)	178.2859		
							C(6)-C(1)-C(2)	117.9395	120.0000	N(13)-C(14)-C(9)-H(23)	-63.2658		
										C(24)-C(14)-C(9)-N(8)	-67.4910		
										C(24)-C(14)-C(9)-C(10)	54.1836		
										C(24)-C(14)-C(9)-H(23)	172.6319		
										H(33)-C(14)-C(9)-N(8)	171.1930		
										H(33)-C(14)-C(9)-C(10)	-67.1324		
										H(33)-C(14)-C(9)-H(23)	51.3160		
										N(8)-C(9)-C(10)-C(5)	-56.8449		
										N(8)-C(9)-C(10)-H(56)	61.9630		
										N(8)-C(9)-C(10)-H(57)	-179.7338		
										C(14)-C(9)-C(10)-C(5)	-179.9455		
										C(14)-C(9)-C(10)-H(56)	-61.1376		
										C(14)-C(9)-C(10)-H(57)	57.1655		
										H(23)-C(9)-C(10)-C(5)	61.4173		
										H(23)-C(9)-C(10)-H(56)	-179.7748		
										H(23)-C(9)-C(10)-H(57)	-61.4717		
										C(7)-N(8)-C(11)-C(12)	-175.6932		
										C(7)-N(8)-C(11)-C(48)	60.2190		
										C(7)-N(8)-C(11)-H(58)	-56.2497		
										C(9)-N(8)-C(11)-C(12)	56.1013		
										C(9)-N(8)-C(11)-C(48)	-67.9865		

Atom	Atom Type (MM2)	Charge (MM2)	Charge (Huckel)	Bond lengths			Bond Angles			Dihedral angles		Close contacts	
				Atoms	Actual (° / Å)	Optimal (° / Å)	Atoms	Actual (° / Å)	Optimal (° / Å)	Atoms	Actual (° / Å)	Atoms	Actual (° / Å)
										C(9)-N(8)-C(11)-H(58)	175.5449		
										Lp(87)-N(8)-C(11)-C(12)	-59.3052		
										Lp(87)-N(8)-C(11)-C(48)	176.6070		
										Lp(87)-N(8)-C(11)-H(58)	60.1384		
										C(7)-N(8)-C(9)-C(10)	57.1833		
										C(7)-N(8)-C(9)-C(14)	-179.7203		
										C(7)-N(8)-C(9)-H(23)	-60.3454		
										C(11)-N(8)-C(9)-C(10)	-176.2566		
										C(11)-N(8)-C(9)-C(14)	-53.1603		
										C(11)-N(8)-C(9)-H(23)	66.2146		
										Lp(87)-N(8)-C(9)-C(10)	-60.6872		
										Lp(87)-N(8)-C(9)-C(14)	62.4092		
										Lp(87)-N(8)-C(9)-H(23)	-178.2159		
										C(4)-C(7)-C(16)-O(17)	110.1094		
										C(4)-C(7)-C(16)-H(59)	-129.9323		
										C(4)-C(7)-C(16)-H(60)	-12.1350		
										N(8)-C(7)-C(16)-O(17)	-120.9636		
										N(8)-C(7)-C(16)-H(59)	-1.0053		
										N(8)-C(7)-C(16)-H(60)	116.7920		
										H(55)-C(7)-C(16)-O(17)	-1.7242		
										H(55)-C(7)-C(16)-H(59)	118.2341		
										H(55)-C(7)-C(16)-H(60)	-123.9686		
										C(4)-C(7)-N(8)-C(9)	-29.4248		
										C(4)-C(7)-N(8)-C(11)	-155.4468		
										C(4)-C(7)-N(8)-Lp(87)	88.2336		
										C(16)-C(7)-N(8)-C(9)	-156.5425		
										C(16)-C(7)-N(8)-C(11)	77.4355		
										C(16)-C(7)-N(8)-Lp(87)	-38.8841		
										H(55)-C(7)-N(8)-C(9)	83.4508		
										H(55)-C(7)-N(8)-C(11)	-42.5712		

Atom	Atom Type (MM2)	Charge (MM2)	Charge (Huckel)	Bond lengths			Bond Angles			Dihedral angles		Close contacts	
				Atoms	Actual (° / Å)	Optimal (° / Å)	Atoms	Actual (° / Å)	Optimal (° / Å)	Atoms	Actual (° / Å)	Atoms	Actual (° / Å)
										H(55)-C(7)-N(8)-Lp(87)	-158.8908		
										C(1)-C(6)-O(15)-C(38)	-39.1733		
										C(1)-C(6)-O(15)-Lp(89)	74.1762		
										C(1)-C(6)-O(15)-Lp(90)	-148.4154		
										C(5)-C(6)-O(15)-C(38)	142.5086		
										C(5)-C(6)-O(15)-Lp(89)	-104.1419		
										C(5)-C(6)-O(15)-Lp(90)	33.2665		
										C(9)-C(10)-C(5)-C(4)	28.2588		
										C(9)-C(10)-C(5)-C(6)	-151.7713		
										H(56)-C(10)-C(5)-C(4)	-93.4818		
										H(56)-C(10)-C(5)-C(6)	86.4881		
										H(57)-C(10)-C(5)-C(4)	148.4062		
										H(57)-C(10)-C(5)-C(6)	-31.6238		
										C(4)-C(5)-C(6)-C(1)	0.7012		
										C(4)-C(5)-C(6)-O(15)	179.1049		
										C(10)-C(5)-C(6)-C(1)	-179.2668		
										C(10)-C(5)-C(6)-O(15)	-0.8630		
										C(3)-C(4)-C(7)-N(8)	178.4917		
										C(3)-C(4)-C(7)-C(16)	-55.4162		
										C(3)-C(4)-C(7)-H(55)	61.5454		
										C(5)-C(4)-C(7)-N(8)	-1.0901		
										C(5)-C(4)-C(7)-C(16)	125.0019		
										C(5)-C(4)-C(7)-H(55)	-118.0364		
										C(3)-C(4)-C(5)-C(6)	0.9919		
										C(3)-C(4)-C(5)-C(10)	-179.0392		
										C(7)-C(4)-C(5)-C(6)	-179.4134		
										C(7)-C(4)-C(5)-C(10)	0.5555		
										C(36)-O(37)-C(3)-C(2)	-0.2454		
										C(36)-O(37)-C(3)-C(4)	179.1628		
										Lp(100)-O(37)-C(3)-C(2)	-167.1726		

Atom	Atom Type (MM2)	Charge (MM2)	Charge (Huckel)	Bond lengths			Bond Angles			Dihedral angles		Close contacts	
				Atoms	Actual (° / Å)	Optimal (° / Å)	Atoms	Actual (° / Å)	Optimal (° / Å)	Atoms	Actual (° / Å)	Atoms	Actual (° / Å)
										Lp(100)-O(37)-C(3)-C(4)	12.2356		
										C(2)-C(3)-C(4)-C(5)	-1.3229		
										C(2)-C(3)-C(4)-C(7)	179.0631		
										O(37)-C(3)-C(4)-C(5)	179.3262		
										O(37)-C(3)-C(4)-C(7)	-0.2877		
										C(1)-C(2)-O(35)-C(36)	-179.2429		
										C(1)-C(2)-O(35)-Lp(99)	22.0397		
										C(3)-C(2)-O(35)-C(36)	-0.2064		
										C(3)-C(2)-O(35)-Lp(99)	-158.9238		
										C(1)-C(2)-C(3)-C(4)	-0.0502		
										C(1)-C(2)-C(3)-O(37)	179.3940		
										O(35)-C(2)-C(3)-C(4)	-179.1442		
										O(35)-C(2)-C(3)-O(37)	0.3000		
										C(2)-C(1)-C(50)-H(75)	14.3688		
										C(2)-C(1)-C(50)-H(76)	131.3435		
										C(2)-C(1)-C(50)-H(77)	-105.1980		
										C(6)-C(1)-C(50)-H(75)	-164.0538		
										C(6)-C(1)-C(50)-H(76)	-47.0792		
										C(6)-C(1)-C(50)-H(77)	76.3793		
										C(5)-C(6)-C(1)-C(2)	-2.0543		
										C(5)-C(6)-C(1)-C(50)	176.3718		
										O(15)-C(6)-C(1)-C(2)	179.6471		
										O(15)-C(6)-C(1)-C(50)	-1.9269		
										C(6)-C(1)-C(2)-C(3)	1.7817		
										C(6)-C(1)-C(2)-O(35)	-179.2798		
										C(50)-C(1)-C(2)-C(3)	-176.7359		
										C(50)-C(1)-C(2)-O(35)	2.2026		

A

Target	Binding energy (Kcal/mol)
MAPK1	–8.5
MAPK3	–9.2

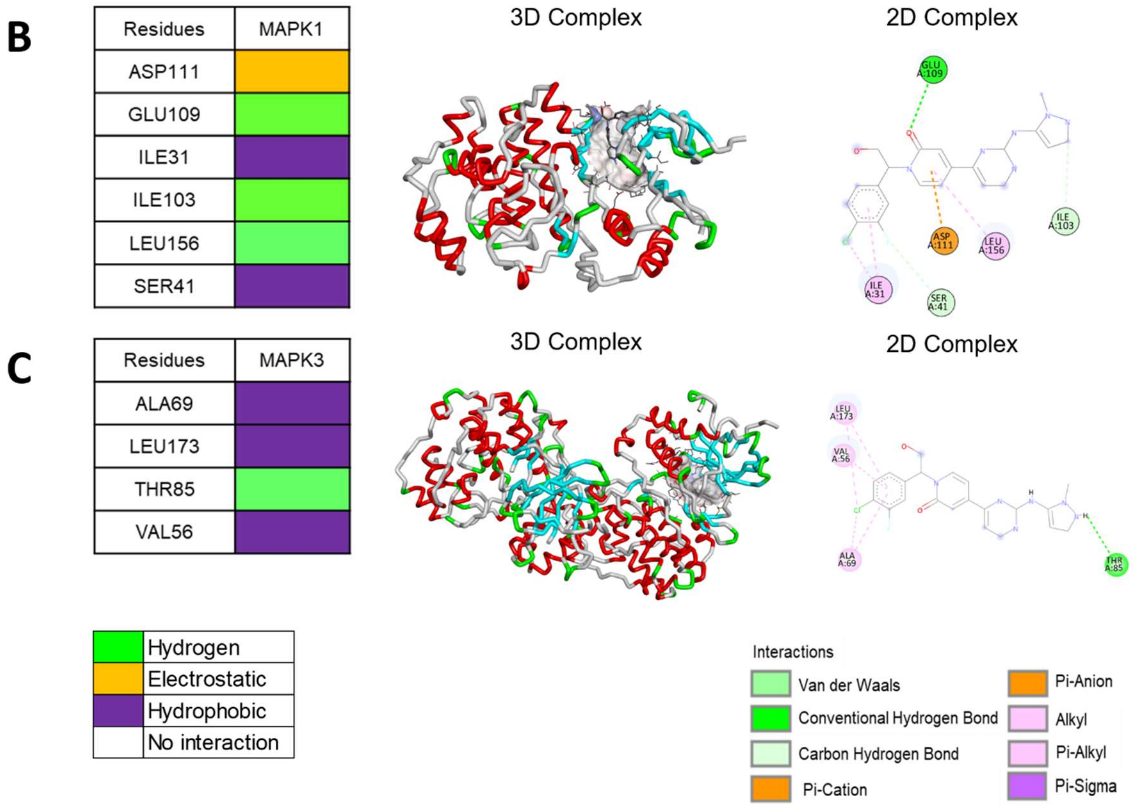

Figure S17. Molecular docking of Ravoxertinib, a known ligand of both MAPK1 (ERK2) and MAPK3 (ERK1). (A) Binding energies indicated that the interactions between Ravoxertinib with both MAPK1 and MAPK3. (B) Interactions between the amino acid residues of MAPK1 and Ravoxertinib. (C) Interactions between the amino acid residues of MAPK3 with Ravoxertinib.

A

Target	Binding energy (Kcal/mol)	
	Compounds	
	Protonated 10	Protonated 11
MAPK1	−9.8	−10.1
MAPK3	−9.7	−9.8

B

C

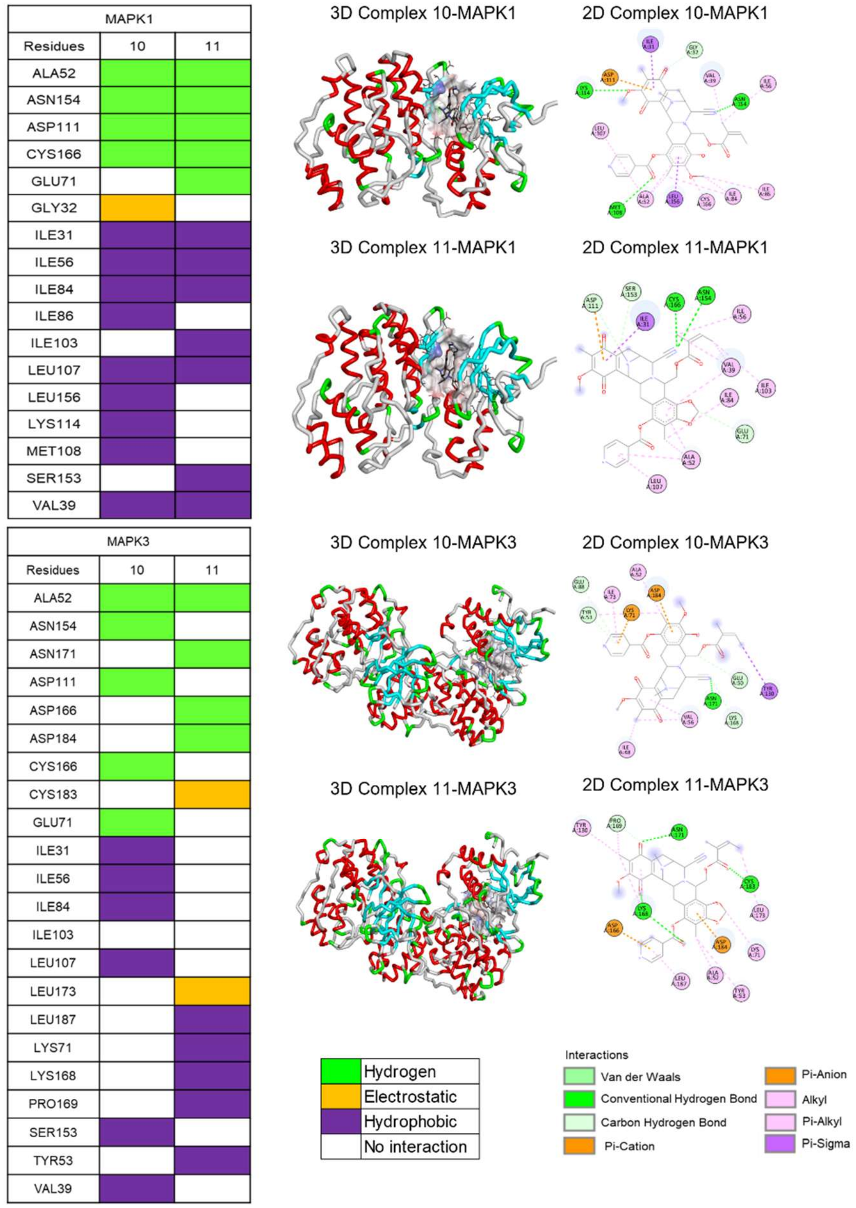

Figure S17. Molecular docking of the protonation state of **10** and **11** with both MAPK1 (ERK2) and MAPK3 (ERK1). (A) Binding energies indicated the interactions of the protonation state of **10** and **11** with both MAPK1 and MAPK3. (B) Interactions between the amino acid residues of MAPK1 and both protonation state of **10** and **11**. (C) Interactions between the amino acid residues of MAPK3 with protonation state of **10** and **11**.

REFERENCES

1. Daikuhara N, Tada Y, Yamaki S, Charupant K, Amnuoypol S, Suwanborirux K, et al. Chemistry of renieramycins. Part 7: Renieramycins T and U, novel renieramycin–ecteinascidin hybrid marine natural products from Thai sponge Xestospongia sp. Tetrahedron Letters. 2009;50(29):4276-8.
